# Prenatal exome sequencing analysis in fetal structural anomalies detected by ultrasonography (PAGE): a cohort study

**DOI:** 10.1016/S0140-6736(18)31940-8

**Published:** 2019-02-23

**Authors:** Jenny Lord, Dominic J McMullan, Ruth Y Eberhardt, Gabriele Rinck, Susan J Hamilton, Elizabeth Quinlan-Jones, Elena Prigmore, Rebecca Keelagher, Sunayna K Best, Georgina K Carey, Rhiannon Mellis, Sarah Robart, Ian R Berry, Kate E Chandler, Deirdre Cilliers, Lara Cresswell, Sandra L Edwards, Carol Gardiner, Alex Henderson, Simon T Holden, Tessa Homfray, Tracy Lester, Rebecca A Lewis, Ruth Newbury-Ecob, Katrina Prescott, Oliver W Quarrell, Simon C Ramsden, Eileen Roberts, Dagmar Tapon, Madeleine J Tooley, Pradeep C Vasudevan, Astrid P Weber, Diana G Wellesley, Paul Westwood, Helen White, Michael Parker, Denise Williams, Lucy Jenkins, Richard H Scott, Mark D Kilby, Lyn S Chitty, Matthew E Hurles, Eamonn R Maher, Prenatal Assessment of Genomes and Exomes Consortium, Mark Bateman, Ian R Berry, Sunayna K Best, Carolyn Campbell, Jenni Campbell, Georgina Carey, Kate E Chandler, Lyn S Chitty, Deirdre Cilliers, Kelly Cohen, Emma Collingwood, Panayiotis Constantinou, Lara Cresswell, Catherine Delmege, Ruth Y Eberhardt, Sandra L Edwards, Richard Ellis, Jerry Evans, Thomas Everett, Clare F Pinto, Natalie Forrester, Emma Fowler, Carol Gardiner, Susan Hamilton, Karen Healey, Alex Henderson, Simon T Holden, Tessa Homfray, Rebecca Hudson, Matthew E Hurles, Lucy Jenkins, Rebecca Keelagher, Mark D Kilby, Tracey Lester, Rebecca Lewis, Jenny Lord, Eamonn R Maher, Tamas Marton, Dominic J McMullan, Sarju Mehta, Rhiannon Mellis, Ruth Newbury-Ecob, Soo-Mi Park, Michael Parker, Katrina Prescott, Elena Prigmore, Oliver W Quarrell, Elizabeth Quinlan-Jones, Simon C Ramsden, Gabriele Rinck, Sarah Robart, Eileen Roberts, Jayne Rowland, Richard H Scott, James Steer, Dagmar Tapon, Emma J Taylor, Madeleine J Tooley, Pradeep C Vasudevan, Astrid P Weber, Diana G Wellesley, Paul Westwood, Helen White, Denise Williams, Elizabeth Wilson

**Affiliations:** a Wellcome Sanger Institute, Hinxton, UK; b West Midlands Regional Genetics Service, Birmingham Women's and Children's National Health Service (NHS) Foundation Trust, Birmingham, UK; c West Midlands Fetal Medicine Centre, Birmingham Women's and Children's National Health Service (NHS) Foundation Trust, Birmingham, UK; d North East Thames Regional Genetics Service, UCL Great Ormond Street Institute of Child Health, Great Ormond Street NHS Foundation Trust, London UK; e The Leeds Genetics Laboratory, St James's University Hospital, Yorkshire Regional Genetics Service, Leeds Teaching Hospitals NHS Trust, Leeds, UK; f Chapel Allerton Hospital, Yorkshire Regional Genetics Service, Leeds Teaching Hospitals NHS Trust, Leeds, UK; g Manchester Centre for Genomic Medicine, Manchester University Hospitals NHS Foundation Trust, Manchester Academic Health Science Centre, Manchester, UK; h Oxford Genomic Medicine Centre, Nuffield Orthopaedic Centre, Oxford, UK; i Department of Cytogenetics, Leicester Royal Infirmary, University Hospitals of Leicester NHS Trust, Leicester, UK; j Department of Clinical Genetics, Leicester Royal Infirmary, University Hospitals of Leicester NHS Trust, Leicester, UK; k Cytogenetics Service, Norfolk and Norwich University Hospital Foundation Trust, Norwich, UK; l West of Scotland Genetics Services, Queen Elizabeth University Hospital, Glasgow, UK; m Northern Genetics Service, Newcastle upon Tyne Hospitals NHS Foundation Trust, Newcastle upon Tyne, UK; n Department of Clinical Genetics, Cambridge University Hospitals NHS Foundation Trust, Cambridge, UK; o South West Thames Regional Genetics Centre, St George's University Hospitals NHS Foundation Trust, London, UK; p Oxford Regional Genetics Services, The Churchill Hospital, Oxford University Hospitals NHS Foundation Trust, Oxford, UK; q Bristol Genetics Laboratory, Southmead Hospital, North Bristol NHS Trust, Bristol, UK; r Department of Clinical Genetics, St Michael's Hospital, University Hospitals Bristol, Bristol, UK; s Department of Clinical Genetics, Sheffield Children's NHS Foundation Trust, Sheffield, UK; t Centre for Fetal Care, Queen Charlotte's and Chelsea Hospital, Imperial College Healthcare NHS Trust, London, UK; u Department of Clinical Genetics, Liverpool Women's NHS Foundation Trust, Liverpool, UK; v Faculty of Medicine, University of Southampton, Southampton, UK; w Wessex Regional Clinical Genetics Service, University Hospital Southampton NHS Foundation Trust, Southampton, UK; x The Ethox Centre, Nuffield Department of Population Health and Wellcome Centre for Ethics and Humanities, University of Oxford, Oxford, UK; y Centre for Women's and Newborn Health, Institute of Metabolism and Systems Research, University of Birmingham, Birmingham, UK; z Department of Medical Genetics, University of Cambridge, Cambridge, UK; aa Cambridge Biomedical Research Centre, National Institute for Health Research, Cambridge, UK

## Abstract

**Background:**

Fetal structural anomalies, which are detected by ultrasonography, have a range of genetic causes, including chromosomal aneuploidy, copy number variations (CNVs; which are detectable by chromosomal microarrays), and pathogenic sequence variants in developmental genes. Testing for aneuploidy and CNVs is routine during the investigation of fetal structural anomalies, but there is little information on the clinical usefulness of genome-wide next-generation sequencing in the prenatal setting. We therefore aimed to evaluate the proportion of fetuses with structural abnormalities that had identifiable variants in genes associated with developmental disorders when assessed with whole-exome sequencing (WES).

**Methods:**

In this prospective cohort study, two groups in Birmingham and London recruited patients from 34 fetal medicine units in England and Scotland. We used whole-exome sequencing (WES) to evaluate the presence of genetic variants in developmental disorder genes (diagnostic genetic variants) in a cohort of fetuses with structural anomalies and samples from their parents, after exclusion of aneuploidy and large CNVs. Women were eligible for inclusion if they were undergoing invasive testing for identified nuchal translucency or structural anomalies in their fetus, as detected by ultrasound after 11 weeks of gestation. The partners of these women also had to consent to participate. Sequencing results were interpreted with a targeted virtual gene panel for developmental disorders that comprised 1628 genes. Genetic results related to fetal structural anomaly phenotypes were then validated and reported postnatally. The primary endpoint, which was assessed in all fetuses, was the detection of diagnostic genetic variants considered to have caused the fetal developmental anomaly.

**Findings:**

The cohort was recruited between Oct 22, 2014, and June 29, 2017, and clinical data were collected until March 31, 2018. After exclusion of fetuses with aneuploidy and CNVs, 610 fetuses with structural anomalies and 1202 matched parental samples (analysed as 596 fetus-parental trios, including two sets of twins, and 14 fetus-parent dyads) were analysed by WES. After bioinformatic filtering and prioritisation according to allele frequency and effect on protein and inheritance pattern, 321 genetic variants (representing 255 potential diagnoses) were selected as potentially pathogenic genetic variants (diagnostic genetic variants), and these variants were reviewed by a multidisciplinary clinical review panel. A diagnostic genetic variant was identified in 52 (8·5%; 95% CI 6·4–11·0) of 610 fetuses assessed and an additional 24 (3·9%) fetuses had a variant of uncertain significance that had potential clinical usefulness. Detection of diagnostic genetic variants enabled us to distinguish between syndromic and non-syndromic fetal anomalies (eg, congenital heart disease only *vs* a syndrome with congenital heart disease and learning disability). Diagnostic genetic variants were present in 22 (15·4%) of 143 fetuses with multisystem anomalies (ie, more than one fetal structural anomaly), nine (11·1%) of 81 fetuses with cardiac anomalies, and ten (15·4%) of 65 fetuses with skeletal anomalies; these phenotypes were most commonly associated with diagnostic variants. However, diagnostic genetic variants were least common in fetuses with isolated increased nuchal translucency (≥4·0 mm) in the first trimester (in three [3·2%] of 93 fetuses).

**Interpretation:**

WES facilitates genetic diagnosis of fetal structural anomalies, which enables more accurate predictions of fetal prognosis and risk of recurrence in future pregnancies. However, the overall detection of diagnostic genetic variants in a prospectively ascertained cohort with a broad range of fetal structural anomalies is lower than that suggested by previous smaller-scale studies of fewer phenotypes. WES improved the identification of genetic disorders in fetuses with structural abnormalities; however, before clinical implementation, careful consideration should be given to case selection to maximise clinical usefulness.

**Funding:**

UK Department of Health and Social Care and The Wellcome Trust.

## Introduction

Approximately 3% of pregnancies will show a fetal structural anomaly in a sonogram, which can range from a single minor defect to severe multisystem anomalies that are fatal.^1^ Genetic investigations are important in the evaluation and clinical triage of fetal structural anomalies. For more than 30 years, conventional prenatal cytogenetic analysis was the first-line method to investigate these anomalies but, within the last 10 years, chromosomal microarray analysis has been increasingly adopted to detect submicroscopic pathogenic copy number variations (CNVs) in prenatal diagnoses.^2^, ^3^ The addition of chromosomal microarray testing to karyotyping increases the frequency of detection of chromosomal abnormalities by 3–5%.^2^, ^3^, ^4^ Fetal structural anomalies can be associated with all types of genetic variation including aneuploidy, uniparental disomy, CNVs, and intragenic mutations. There is increasing interest in genome-wide sequencing strategies to investigate prenatally detected congenital abnormalities. Prenatal whole-genome sequencing (WGS) has previously been described,^5^ but whole-exome sequencing (WES) and targeted gene panels have received more interest because of their lower cost, the lower amounts of fetal DNA required, the possibility of comparatively more rapid turnaround, and greater sequencing depth.^6^, ^7^, ^8^, ^9^, ^10^, ^11^, ^12^, ^13^ We previously used WES in 29 fetal-parental trios with a fetal structural anomaly, and we identified an underlying genetic cause in 10% of cases.^7^ In the investigation of a range of fetal structural anomalies, WES has shown variants in genes associated with developmental disorders in more than 50% of fetuses in investigated cohorts, but most previous studies^14^, ^15^ have used small sample sizes (of 30 fetuses or fewer), are confined to highly selected subgroups (eg, those that were attained at autopsy), or both (appendix).

**Research in context**

**Evidence before this study**

Approximately 3% of pregnancies will show a fetal structural anomaly in a routine prenatal ultrasound, and genetic investigations are important in assessment of these cases. Chromosomal microarray analysis, which increases the frequency of diagnoses of chromosomal abnormalities by 3–5%, has increasingly superseded conventional cytogenetic testing. There is increasing interest in genome-wide sequencing strategies, such as whole-exome sequencing (WES), in identification of genetic causes of congenital abnormalities and the addition of WES to chromosomal microarray has greatly increased the frequency with which genetic causes are detected through genetic testing in children with developmental disorders. However, evidence regarding the usefulness of WES for fetal structural anomalies is inadequate. We searched PubMed for studies of whole-exome or genome sequencing that had been published in English on or before Nov 1, 2017. We used the search terms “genome”, “anomaly”, and “malformation” to identify relevant studies. Most reports included a small number (<30 cases) of highly selected subgroups of fetuses with a narrow range of structural anomalies and provided inadequate information regarding the likely frequency with which genetic causes are detected from the application of WES for a broad range of fetal structural anomalies in clinical practice.

**Added value of this study**

To our knowledge, this is the first large-scale prospectively ascertained cohort of fetal structural anomalies that has been detected with prenatal ultrasound (in which aneuploidy and large copy number variants had been excluded). After bioinformatic filtering of the WES data according to allele frequency and effect on protein and inheritance pattern and assessment by a multidisciplinary clinical review panel as to whether a WES finding was pathogenic and causative, we detected diagnostic abnormalities in 8·5% of fetuses with structural anomalies. These data suggest that the addition of prenatal WES to chromosomal microarray would increase the detection of genetic causes of fetal structural anomalies and would provide important information on prognosis and future recurrence risks.

**Implications of all the available evidence**

Although WES increases the frequency of identification of genetic causes of structural anomalies in fetuses more than cytogenetics or chromosomal microarray alone, the overall frequency of diagnostic genetic findings in a series comprising a broad range of prenatally detected fetal structural anomalies is lower than was previously reported in smaller, more biased series of fetus structural anomalies and in children with developmental disorders who had been selected after genetic evaluation. Given the practical, diagnostic, and ethical challenges of prenatal diagnosis, genome-wide sequencing for fetal structural anomalies is best applied to selected subgroups (eg, those with multiple congenital anomalies) or after genetic review.

To determine the usefulness of genome-wide sequencing strategies in prenatal diagnosis of fetal structural anomalies a large-scale sequencing project, the Prenatal Assessment of Genomes and Exomes (PAGE) study, was established. In our study, as part of this larger project, we aimed to report the WES results from 610 fetuses (and parental samples) with a wide range of fetal structural anomalies, and to highlight the ethical and practical issues that we encountered that have implications for the translation of prenatal WES into clinical practice.

## Methods

### Study design and participants

In this prospective cohort study, two groups in Birmingham and London recruited patients from 34 fetal medicine units in England and Scotland (appendix). If fetal structural anomalies were detected during a routine detailed ultrasound scan at any of these units, parents who opted for invasive testing were offered participation in the PAGE study.

Women were eligible for inclusion if they were undergoing invasive testing for identified nuchal translucency or structural anomalies in their fetus, as detected by ultrasound after 11 weeks of gestation. The partners of these women also had to consent to participate. Women were excluded if abnormal aneuploidy considered to have caused the structural abnormality was detected, if one or both parents were younger than 16 years, or if one or both parents did not or could not provide informed consent. All participants gave written informed consent, and the study was approved by Research and Development offices at each participating institution and by relevant Research Ethics Committees (including those at South Birmingham and Harrow).

### Procedures

Parental blood samples were collected for DNA extraction and fetal DNA was obtained from chorionic villi, amniotic fluid, or fetal blood that remained after routine investigations at the two coordinating centres. This DNA was assessed for aneuploidy and CNVs at these centres. Parents and fetuses were excluded from subsequent analyses if tests revealed aneuploidy or CNVs that explained the anomalous structural phenotype of the fetus. DNA of parents and fetuses that had not been excluded was then shipped to the Wellcome Sanger Institute for WES (appendix). Participants were informed that the PAGE genetic analyses results would not be available during the current pregnancy, and only results relevant to the ultrasound scan-detected fetal structural anomalies would be reported back to parents. To ensure a range of phenotypes, it was agreed before the study that the number of fetuses with any specific phenotype would be capped at about 20% of the ongoing total.

After WES, we assessed sequence data for candidate pathogenic variants from a modified list of genes that are likely associated with developmental disorders (appendix),^16^ and we selected rare, protein altering variants in which the inheritance pattern of the variant matched that of the gene being assessed for clinical review (bioinformatic filtering). These variants were deemed candidate pathogenic variants and were collated for assessment by a clinical review panel (CRP). The sequence data that we used to determine likely genes and variants associated with developmental disorders are available from the European Genome-phenome Archive.

Candidate pathogenic variants were reviewed and classified by a CRP that comprised at least six participants (a clinical geneticist, fetal medicine specialist, two clinical scientists, and a bioinformatician) from the study team and, usually, clinical geneticist and laboratory scientist from the recruitment centre. Initially, CRP meetings were face-to-face but, subsequently, distant participants joined by Webex or teleconferencing to review anonymised variant annotation data and clinical findings through the Sapientia version 1.7.5 (Congenica; Cambridge, UK) software. The CRP reached a consensus decision regarding variant classification (ie, pathogenic, likely pathogenic, variant of uncertain significance, likely benign, or benign), and to the likelihood that they caused the structural abnormality phenotype detected in the fetus, based on standard criteria in the UK National Health Service laboratories at the time of the relevant CRP meeting (these guidelines by the Association of Clinical Genetic Science, which are based on American College of Medical Genetics and Genomics guidelines, are available online). Variants in genes associated with developmental disorders that were determined by the CRP to be pathogenic or likely pathogenic and causative of the fetal phenotype were referred to as diagnostic genetic variants. The CRP also agreed on the contribution of a genetic variant to fetal structural anomalies (ie, none, uncertain, partial, or full) based on the ultrasound findings. In accordance with the ethical approval restrictions, pathogenic or likely pathogenic variants were only reported to relevant clinicians to report to the parents if they were considered causative (partly or fully) of the fetal structural anomalies.

During the study, it became apparent that a pathogenic *RIT1* variant had not been reviewed by the CRP after the variant had been removed from the dataset during bioinformatic filtering of variants because it had been inherited from a parent who was determined to be unaffected by developmental disorders. Reanalysis of the data for other known pathogenic variants (from the ClinVar database of genetic variation and human health) in developmental genes then revealed two further similar fetuses with an inherited pathogenic variant in *PTPN11*, who had hydrops or large nuchal translucency. Sanger sequencing was used to confirm the presence of all identified diagnostic genetic variants and research reports were issued to the relevant clinician. Benign and likely benign variants were not validated or reported, but variants of unknown significance (VUS) that the CRP considered to have potential clinical usefulness were validated. The CRP comprised both core and rotating participants, facilitating consistency and mitigating possible bias in interpretation.

### Outcomes

The primary endpoint, which was assessed in all fetuses, was the detection of diagnostic genetic variants considered to have caused the fetal developmental anomaly. We also assessed the prespecified exploratory endpoint of the frequency of genetic variants in predefined subgroups of fetuses with specific structural anomalies.

### Statistical analysis

Variants were annotated with the probability of the relevant gene being loss-of-function-intolerant.^17^ These probabilities were compared with the Mann-Whitney U test. The number of diagnostic genetic variants in the phenotypic classes were compared with Fisher's exact test, and Bonferroni correction for multiple testing was done with the p.adjust R package. All statistical analyses were done with R (version 3.1.3).

### Role of the funding source

The funder of the study had no role in study design, data collection, data analysis, data interpretation, or writing of the report. The corresponding author had full access to all the data in the study and had final responsibility for the decision to submit for publication.

## Results

The cohort was recruited between Oct 22, 2014, and June 29, 2017, and clinical data were collected until March 31, 2018. To estimate the number of eligible cases that were excluded from WES, a retrospective review of 564 eligible fetuses (ie, fetuses with a structural abnormality) revealed that 134 (23·8%) samples were not sent for WES because of an abnormal quantitative fluorescence-PCR analysis (n=97) or chromosomal microarray (n=37) finding.

The 610 fetuses (257 female, 353 male) that were eligible for WES were categorised into 11 phenotypic classes based on the site of the anomalies that were detected by ultrasound (appendix). These phenotypic anomaly groups ranged from spinal (n=10) to complex or multisystem anomalies (ie, in which two or more fetal structural anomalies were detected; n=143; appendix). These 610 fetuses and 1202 matched parental samples (596 fetus-parental trios, including two sets of twins, and 14 fetus-parent dyads) were included in the cohort. 321 genetic variants (representing 255 potential diagnoses) were identified as candidate diagnostic findings from the WES data and were reviewed by the CRP (appendix). A mean of 0·42 (SD 0·676) potential diagnoses per fetus was reviewed; this value included a mean of 0·40 (0·636) for complete trios (n=596) and 1·36 (1·393) for dyads (n=14). However, phenotypes did not significantly differ between trios and dyads.

**Figure 1:**
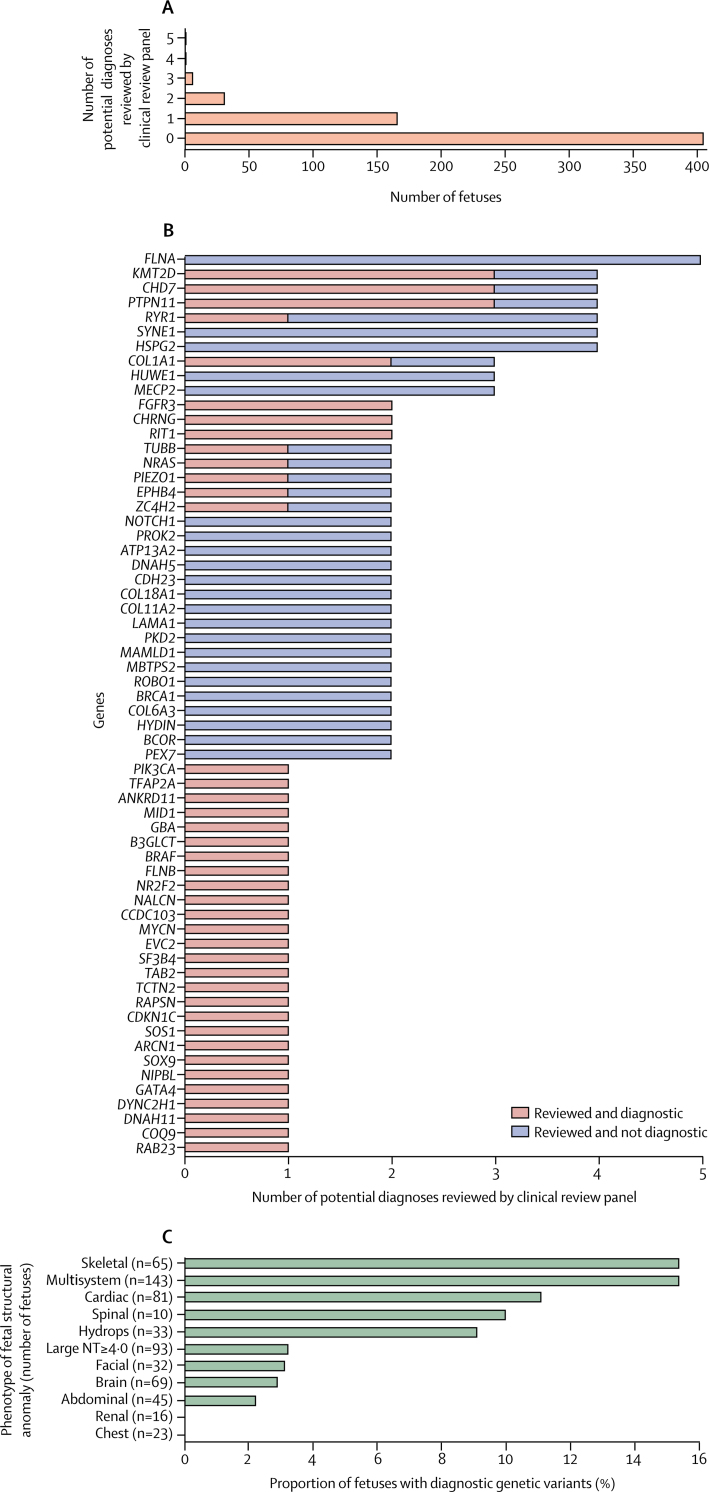
Features of the potential diagnoses in fetuses with structural abnormalities (A) Number of potential diagnoses per fetus that were reviewed by the clinical review panel. (B) Number of potential diagnoses reviewed by the clinical review panel by gene, for all genes with a pathogenic or likely pathogenic variant and for all genes considered in more than one fetus (regardless of diagnostic status, single nucleotide variants and indels only). (C) Proportion of diagnostic genetic variants identified in fetuses with each phenotypic abnormality. NT=nuchal translucency.

The CRP assessed variants in 184 different developmental disorder genes, at a median of one gene in each fetus. However, 35 genes were assessed in several fetuses (*FLNA* in five fetuses; *HSPG2, RYR1, SYNE1, KMT2D, CHD7*, and *PTPN11* in four fetuses each; *MECP2, COL1A1*, and *HUWE1* in three fetuses each; and *ATP13A2, DNAH5, CDH23, COL18A1, COL11A2, LAMA1, MBTPS2, MAMLD1, PKD2, NOTCH1, PROK2, FGFR3, BRCA1, CHRNG, COL6A3, ROBO1, TUBB, PIEZO1, NRAS, HYDIN, ZC4H2, BCOR, PEX7, EPHB4*, and *RIT1* in two fetuses each; figure 1).

Pregnancy outcomes were available for 474 (78%) of 610 fetuses. Of these, in 142 (30%) pregnancies, the parents opted for termination, 14 (3%) pregnancies ended in miscarriage, there were 22 (5%) stillbirths, 14 (3%) neonatal deaths, and 282 (59%) were livebirths.

A detailed study of the ethical issues in the PAGE study is in progress.^18^ Possible ethical issues that we noted included the identification of potentially pathogenic variants that might confer a risk of recurrence of an inherited developmental disorder but were unrelated to the detected fetal structural anomalies and the identification of a VUS in a relevant candidate gene; in accordance with the ethical approval, these findings were not reported to the parents. Postnatally, this issue might be handled by more detailed phenotyping or periodic reviews but, in the prenatal setting, phenotypic information is generally less detailed and delaying a diagnostic decision is usually not an option. This decision is particularly difficult if the fetal structural anomalies might have a benign prognosis, such as talipes equinovarus. Other ethical issues regarding not reporting outcomes to parents arose with detection of heterozygous pathogenic variants in developmental genes associated with autosomal recessive disease and detection of pathogenic variants that predict late-onset adult disease (eg, increased risk of breast cancer in a mother found to be a carrier of a Fanconi anaemia gene variant) that was not relevant to the fetal abnormality being studied.

The 321 genetic variants (255 potential diagnoses) included 301 single nucleotide variants (SNVs) and indels, 18 CNVs, and two uniparental disomies in 205 (33·6%) of 610 fetuses that underwent WES. 52 (8·5%, 95% CI 6·4–11·0) fetuses were diagnosed with likely pathogenic or pathogenic variants relevant to the phenotypic fetal structural anomalies (table 1). 32 (61·5%) of 52 fetuses with a diagnostic genetic finding (ie, a genetic result considered to be causative of the structural abnormality observed) had a de novo mutation (15 truncating mutations, 15 missense mutations, one in-frame insertion, and one 41·2 kb deletion), 19 (36·5%) fetuses had inherited the relevant mutations (14 mutations were autosomal recessively inherited, and five mutations were dominantly inherited disorders), and one (1·9%) fetus had a chromosome 15 uniparental disomy.

**Table 1:** Diagnostic variants that were identified and classified by the Prenatal Assessment of Genomes and Exomes clinical review panel

	**Phenotype**	**Gene**	**Consequence**	**Inheritance**	**Zygosity**
PP0087	Skeletal	*DYNC2H1*	Two stop gained mutations	Inherited	Compound heterozygous
PP0174	Multisystem	*NRAS*	Missense variant	De novo	Heterozygous
PP0184	Cardiac	*NR2F2*	Missense variant	De novo	Heterozygous
PP0204	Skeletal	*ZC4H2*	Frameshift variant	De novo	Heterozygous
PP0258	Abdominal	*MYCN*	Missense variant	Inherited from affected parent	Heterozygous
PP0318	Skeletal	*CHRNG*	Frameshift variant	Inherited	Homozygous
PP0333	Cardiac	*GATA4*	Frameshift variant	De novo (presumed)	Heterozygous
PP0342	Multisystem	*CHRNG*	Frameshift variant	Inherited	Compound heterozygous
PP0384	Brain	*B3GLCT*	Splice donor variant	Inherited	Homozygous
PP0390	Cardiac	*CCDC103*	Missense variant	Inherited	Homozygous
PP0513	Cardiac	*DNAH11*	Stop gained	Inherited	Homozygous
PP0555	Multisystem	*EVC2*	Frameshift variant	Inherited	Homozygous
PP0602	Large NT ≥4·0	NA	Uniparental disomy on chromosome 15	Uniparental disomy	NA
PP0656	Multisystem	*PKD1/TSC2*	41·2kb deletion	De novo (copy number variation)	Heterozygous
PP0659	Multisystem	*RAPSN*	Splice donor variant	Inherited	Homozygous
PP0792	Skeletal	*COL1A1*	Missense variant	De novo	Heterozygous
PP0981	Multisystem	*GBA*	Frameshift variant	Inherited	Homozygous
PP1408	Multisystem	*SOX9*	Missense variant	De novo	Heterozygous
PP1462	Multisystem	*BRAF*	Missense variant	De novo	Heterozygous
PP1561	Skeletal	*PIK3CA*	Missense variant	De novo	Heterozygous
PP1573	Hydrops	*KMT2D*	Frameshift variant	De novo	Heterozygous
PP1579	Brain	*TUBB*	Missense variant	De novo	Heterozygous
PP1627	Multisystem	*PIEZO1*	Three missense variants	Inherited	Compound heterozygous
PP1711	Facial or cleft lip and palate	*SF3B4*	Frameshift variant	De novo	Heterozygous
PP1726	Cardiac	*TAB2*	Frameshift variant	De novo	Heterozygous
PP1750	Cardiac	*ANKRD11*	Frameshift variant	De novo	Heterozygous
PP1753	Multisystem	*CDKN1C*	Frameshift variant	Inherited	Heterozygous
PP1780	Multisystem	*TCTN2*	Splice acceptor	Inherited	Homozygous
PP1795	Multisystem	*COQ9*	Stop gained	Inherited	Homozygous
PP1807	Large NT ≥4·0	*MID1*	Stop gained	De novo	Hemizygous
PP1843	Multisystem	*KMT2D*	Stop gained	De novo	Heterozygous
PP1864	Cardiac	*KMT2D*	Splice donor variant	De novo	Heterozygous
PP1892	Cardiac	*SOS1*	Protein altering variant	De novo	Heterozygous
PP1934	Skeletal	*COL1A1*	Missense variant	De novo	Heterozygous
PP1967	Multisystem	*PTPN11*	Missense variant	De novo	Heterozygous
PP2000	Multisystem	*RYR1*	Stop gained and frameshift variant	Inherited	Compound heterozygous
PP2009	Skeletal	*ARCN1*	Frameshift variant	De novo	Heterozygous
PP2015	Multisystem	*FLNB*	Missense variant	De novo	Heterozygous
PP2033	Cardiac	*CHD7*	Frameshift variant	De novo	Heterozygous
PP2039	Hydrops	*NIPBL*	Stop gained	De novo	Heterozygous
PP2141	Skeletal	*FGFR3*	Missense variant	De novo	Heterozygous
PP2645	Multisystem	*TFAP2A*	Missense variant	De novo	Heterozygous
PP2718	Multisystem	*CHD7*	Stop gained	De novo	Heterozygous
PP2904	Spinal	*EPHB4*	Frameshift variant	De novo	Heterozygous
PP2979	Multisystem	*CHD7*	Frameshift variant	De novo	Heterozygous
PP3168	Multisystem	*RIT1*	Missense variant	De novo	Heterozygous
PP3246	Skeletal	*NALCN*	Missense variant	De novo (presumed)	Heterozygous
PP3387	Multisystem	*RAB23*	Stop gained	Inherited	Homozygous
PP3540	Skeletal	*FGFR3*	Missense variant	De novo	Heterozygous
PP0626	Multisystem	*RIT1*	Missense variant	Inherited	Heterozygous
PP2567	Hydrops	*PTPN11*	Missense variant	Inherited	Heterozygous
PP0503	Large NT ≥4·0	*PTPN11*	Missense variant	Inherited	Heterozygous

Data are listed by identification numbers given by the clinical review panel. NA=not applicable. NT=nuchal translucency.

We found that genetic variants were diagnostic (ie the variant was considered pathogenic or likely pathogenic and causative of the fetal phenotype) in 15 (75·0%) of 20 fetuses with de novo protein truncating variants in monoallelic genes, 15 (25·0%) of 60 fetuses with de-novo missense variants in monoallelic genes, and 31 (37·8%) of 82 fetuses with de-novo variants in a monoallelic disease gene. Genes with diagnostic SNVs and indels had a higher median probability of being loss-of-function intolerant than non-diagnostic variants (diagnostic 0·81 *vs* non-diagnostic 0·24; p=0·0276).

Of the 35 genes with variants reviewed by the CRP in more than one fetus, six (17%) genes had variants that were diagnostic in more than one fetus (figure 1), and diagnostic *KMT2D* and *CHD7* mutations (all de novo truncating) were present in fetuses with several phenotypic anomalies (*KMT2D*: one fetus with each of multisystem anomalies, cardiac anomalies, and hydrops; *CHD7*: two fetuses with multisystem anomalies and one fetus with cardiac anomalies). *PTPN11* variants (all missense, one de novo, two inherited) were found in fetuses with multisystem anomalies, hydrops, and increased nuchal translucency phenotypes. To our knowledge, this PAGE cohort includes the first instances of the prenatal identification of mutations in several genes (table 2; appendix).

**Table 2:** Genes identified by the PAGE clinical review panel that had diagnostic variants without previous prenatal phenotype descriptions

	**Postnatal fetal phenotypes**	**References**	**Number of postnatal cases in cited references**	**Prenatal ultrasound scan findings (PAGE identifier)**
*ANKRD11*	KBG syndrome, Coffin-Siris-like syndrome, intellectual disability, macrodontia, facial dysmorphisms, skeletal anomalies, short stature, hearing loss, and recurrent middle palatal abnormalities	19, 20, 21	89	Atrioventricular canal defect (PP1750)
*ARCN1*	Severe micrognathia, microcephaly, short stature with rhizomelic shortening, joint laxity, and mild developmental delay and, in some cases, cardiac defects, and cleft palate (each in one case)	22	4	Absent or hypoplastic radius, ulnar hypoplasia, fibular hypoplasia, and short tibia, femur, and humerus (PP2009)
*CCDC103*	Primary ciliary dyskinesia (including upper and lower airway infections, sinusitis, bronchiectasis, dextrocardia or situs inversus, atrioventricular septal defects, and immotile sperm)	23, 24, 25, 26	14	Complex univentricular heart, double outlet right ventricle, transposition great arteries, pulmonary stenosis, and likely right atrial isomerism (PP0390)
*COQ9*	Neonatal encephalopathy with lactic acidosis, seizures, global developmental delay, hypertrophic cardiomyopathy, and renal tubular dysfunction	27, 28	2	Dilated loops of bowel, cardiomegaly, pericardial effusion, fetal growth restriction, anhydramnios (PP1795)
*MYCN*	Feingold syndrome (including oesophogeal and duodenal atresias, microcephaly, learning disabilities, and digital anomalies such as brachymesophalang or syndactyly), cardiac defects, and renal anomalies	29	77	Duodenal atresia (PP0258)
*NR2F2*	Atrioventricular septal defects, atrial septal defects, hypoplastic left heart syndrome, coarctation of the aorta, tetralogy of Fallot, and congenital diaphragmatic hernia	30, 31	11	Abnormal four-chamber view of the heart (PP0184)
*TAB2*	Frontometaphyseal dysplasia, hypertelorism, wide nasal bridge, micrognathia, hearing loss, congenital heart defects (variable), scoliosis, and upper limb contractures	32, 33, 34, 35	15	Increased nuchal translucency of 8·0 mm (PP1726)
*TUBB*	Microcephaly, structural brain anomalies (including dysmorphic basal ganglia, abnormalities of the corpus callosum, and brainstem hypoplasia), learning disability, circumferential skin creases, cleft palate, and short stature	36, 37	6	Dysgenesis of the corpus callosum and lissencephaly (PP1579)
*ZC4H2*	Arthrogryposis multiplex congenita, kyphosis or scoliosis, and severe learning disabilities	38	5	Fixed extended knees, rocker bottom feet, and a flat forehead (PP204)

PAGE=Prenatal Assessment of Genomes and Exomes.

The proportion of fetuses with diagnostic genetic variants varied between the phenotypic groups (figure 1; appendix). The greatest proportion of diagnostic genetic variants were found in fetuses with skeletal abnormalities (ten [15·4%] of 65 fetuses with skeletal abnormalities), multisystem anomalies (22 [15·4%] of 143 fetuses with multisystem anomalies), and cardiac abnormalities (nine [11·1%] of 81 fetuses with cardiac abnormalities). We found diagnostic genetic variants in three (9·0%) of 33 fetuses with hydrops and one (10·0%) of ten fetuses with spinal abnormalities. Diagnostic genetic variants were found in less than 4% of fetuses in all other groups of phenotypic anomalies. After correction for multiple testing, the detection of pathogenic or likely pathogenic genetic variants in fetuses with multisystem anomalies was significantly more frequent than in fetuses with all other phenotypes (p=0·01893).

The consequences of a molecular diagnosis might relate to both the affected pregnancy and potential future pregnancies. None of the diagnostic genetic variants would have led to in-utero fetal treatment. However, diagnoses during pregnancy could have affected decisions about whether to proceed with the pregnancy; for instance, in the fetuses with cardiac anomalies, pathogenic or likely pathogenic variants were found in genes associated with postnatal extracardiac manifestations (such as in *KMT2D, ANKDR11, SOS1, CCDC103,* and *CHD7*), including learning disabilities. Overall 34 (65·4%) of 52 diagnostic genetic variants were associated with learning disabilities and, of these, 16 (47·1%) genetic variants were in a fetus without brain or multisystem anomalies (table 1). During pregnancies, diagnoses might have enabled better postnatal management (such as monitoring for neonatal hypoglycaemia in a fetus with exomphalos and a *CDKN1C* mutation), and it has been suggested that CoQ10 treatment might be helpful in COQ9-deficient children.^27^ Future consequences of a positive diagnosis were a low recurrence risk in 33 fetuses (5% of all fetuses assessed; 31 fetuses with de novo mutations in monoallelic disease genes, one fetus with a de-novo CNV, and one fetus with a uniparental disomy) and a high recurrence risk in 19 fetuses with inherited variants (3% of all fetuses assessed; 14 autosomal recessive mutations and five dominant disorders).

**Figure 2:**
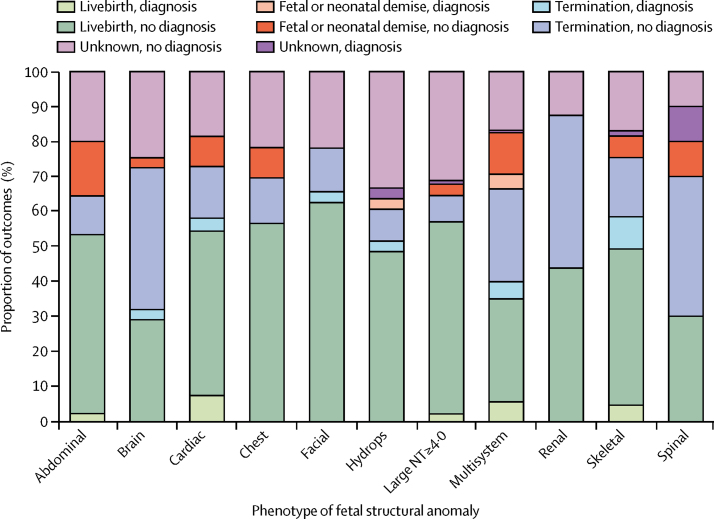
Pregnancy outcomes associated with different fetal structural anomalies 45 fetuses had abdominal anomalies, 69 fetuses had brain anomalies, 81 fetuses had cardiac anomalies, 23 fetuses had thoracic anomalies, 32 fetuses had facial or cleft lip and palate anomalies, 33 fetuses had hydrops, 93 fetuses had increased nuchal translucency (more than 4·0 mm), 16 fetuses had renal anomalies, 65 fetuses had skeletal anomalies, ten fetuses had spinal anomalies, and 143 fetuses had complex or multisystem anomalies. NT=nuchal translucency.

Of the 52 fetuses with a WES diagnosis of likely pathogenic or pathogenic genetic variants, post-mortem or postnatal follow up was available in 47 (90·4%) fetuses and was consistent with the molecular diagnosis. To our knowledge, a postnatal genetic diagnosis has not been made in any of the fetuses that were reviewed by the CRP and designated as having a variant without clinical relevance. Of the 474 fetuses with available pregnancy outcome data, pathogenic or likely pathogenic genetic variants were detected in 27 (14·1%) of 192 fetuses that did not survive beyond birth, including in two (14%) of 14 miscarriages, 20 (14·1%) of 142 terminations of pregnancy, three (13·6%) of 22 stillbirths, and two (14·3%) of 14 neonatal deaths, which was significantly more common than in the 20 (7·1%) of 282 fetuses that were liveborn (p=0·0181; figure 2).

In addition to 52 WES-diagnosed fetuses, a further 24 fetuses showed genetic variants that were not considered to be classifiable as diagnostic but merited further (post-hoc) clinical and molecular investigations and these were classified as clinically relevant-VUSs (appendix). These findings included a fetus with micrognathia, radial aplasia, ulnar and fibular hypoplasia, tibial and femoral shortening, and an abnormal lumbar spine with compound heterozygous nonsense (2269C→T; Gln757Ter) and missense (1580C→G; Thr527Arg) variants in *RECQL4.* Although the nonsense variant was considered pathogenic, the missense sub_stitution was classified as a VUS. Bi-allelic *RECQL4* mutations are associated with radial aplasia and hypoplasia syndromes, and we considered further follow up to be indicated. In another case (PP0722) a de-novo missense *KMT2D* variant was detected in a fetus with a 6·7 mm nuchal translucency during the first trimester. In another case (PP1720), an apparently pathogenic de-novo nonsense variant in *CHD7* was detected in a fetus with mild lateral ventriculomegaly and no other structural brain abnormalities. Although hydrocephalus has rarely been reported in conjunction with pathogenic *CHD7* variants, it was believed that the isolated ventriculomegaly could not be unequivocally attributed to the *CHD7* variant in the absence of any other features of CHARGE syndrome. This example illustrates the difficulties that can occur in interpreting genotype-phenotype causality during the prenatal period. With the 24 cases that showed potentially clinically relevant variants in addition to the 52 diagnostic cases, we found 76 (12·5%, 95% CI 9·9–15·3) of 610 fetuses in which WES provided a clinically relevant result.

## Discussion

In our large prospective cohort study of 610 fetuses with a broad range of fetal structural anomalies that had been detected by prenatal ultrasound scan, we identified a relevant diagnostic genetic variant in a developmental disorder gene in 52 (8·5%) fetuses. In an additional 24 (3·9%) fetuses, a variant of potential clinical usefulness was identified and reported. Overall, we identified a diagnostic or potentially clinically relevant variant in 76 (12·5%) fetuses. Although some previous studies^14^ of fetal structural anomalies have reported diagnostic genetic variants in more than 50% of fetuses with a structural abnormality, most previous studies^14^ comprise small numbers of selective cases, and the designation of genetic variants as diagnostic was less stringent. The largest previous study^11^ used WES in 84 deceased fetuses and found diagnostic genetic variants in 20% of these fetuses. Our lower number of fetuses with diagnostic genetic variants reflects differences in ascertainment methods: we prospectively recruited all suitable cases and undertook WES without genetic review (after excluding aneuploidy and large CNVs) whereas Yates and colleagues^11^ studied deceased fetuses after termination or spontaneous fetal death. Around 60% of our cohort were liveborn (and a lower number had diagnostic genetic variants than fetuses who did not survive beyond birth), and the number of fetuses that did not survive that showed diagnostic genetic variants in our study was close to that reported by Yates and colleagues.^11^ The results of genome-wide sequencing in unselected idiopathic fetal structural anomalies are especially relevant when considering the potential for translating WES into clinical practice. We note that, in a meeting abstract, Wapner and colleagues^39^ reported a causal pathogenic variant in 7·5% of sequential cases of fetal structural anomalies (and a further 5·5% had a karyotype or chromosomal microarray anomaly).

WES detection of diagnostic genetic variants in fetal structural anomalies is significantly less frequent than that reported in children with developmental disorders (in whom these genetic variants in developmental disorder genes have been reported in up to 43% of cohorts) despite a similar sequencing and interpretation strategy.^16^, ^40^ This disparity reflects differences in ascertainment, since the previous postnatal cohort^16^, ^40^ was selected after assessment by a clinical geneticist (and therefore enriched for likely monogenic disorders), whereas the PAGE cohort includes manifestations such as isolated large nuchal translucency, isolated talipes, and neural tube defects, all of which have a low association with a monogenic basis. Additionally, greater imprecision in prenatal versus postnatal phenotyping could also contribute (eg, postnatally, expert dysmorphology and developmental assessment facilitates variant interpretation, which increases the frequency at which diagnostic genetic variants are detected).

We found diagnostic genetic variants more frequently in association with cardiac, complex or multisystem, and skeletal anomalies and, to a lesser extent, hydrops fetalis and spinal abnormalities; however, diagnostic genetic variants were detected in less than 4% of fetuses with other types of anomalies. Variants in *KMT2D* were one of the most frequent diagnostic findings, and these variants were associated with several phenotypes, including multisystem anomalies (PP1843), isolated complex cardiac defects (PP1864), and fetal hydrops and cystic hygroma (PP1573). *KMT2D* mutations cause Kabuki syndrome, which is characterised postnatally by developmental delay, epilepsy, cardiac, genitourinary and musculoskeletal anomalies, and distinctive facial features.^41^, ^42^, ^43^, ^44^ Although the presentation of *KMT2D* mutations with fetal hydrops has been reported previously,^44^, ^45^ the distinctive facial dysmorphology is less apparent in infancy than older children, which makes diagnosis difficult during the early postnatal period.

To date, prenatal WES studies have implicated many developmental genes in fetal structural abnormalities, but 19 genes (including *KMT2D*) have been reported in several studies (appendix). To our knowledge, we report the first prenatally diagnosed mutations in several genes (table 2), including those associated with isolated (*NR2F2* and *TAB2*) and syndromic congenital heart disease (primary ciliary dyskinesia associated with *CCDC103* and KBG syndrome associated with *ANKRD11*), which indicates that WES can provide important additional information on non-cardiac prognosis. WES can also provide insight into the risks of recurrence in subsequent fetuses to inform future reproductive choices. Although 19 (36·5%) of 52 fetuses with diagnostic genetic variants in our study were determined to have a high recurrence risk, many genetic variants resulted from de-novo mutations that were associated with a small increased recurrence risk from gonadal mosaicism. In instances of genetic variants with a recurrence risk, parents might wish to seek preimplantation diagnosis or prenatal genetic diagnosis through invasive or non-invasive prenatal diagnosis in future pregnancies.^46^

A limitation of our study was that the protocol does not deliver real-time diagnoses during pregnancies. However, decisions on variant classification, validation, and reporting were based on information that would have been available for an ongoing pregnancy and provide insights into translating prenatal WES into clinical practice. To maximise the informativeness of the WES analysis across a range of fetal structural anomalies phenotypes, we capped the number of fetuses with isolated nuchal translucency that we analysed. At the time of the cap, isolated nuchal translucency fetuses accounted for 22% of the total cohort. We estimate that, if the cap had not been applied, an additional 56 fetuses would have been analysed, and we estimate that approximately one additional diagnosis would have been made, giving an overall estimated proportion of fetuses with diagnostic variants of 8%. For rapid and efficient variant prioritisation, fetal-parental trio analysis is preferable to fetus-only WES because assessment of trios enables rapid identification of de-novo variants in monoallelic developmental disorder genes and defines whether heterozygous pathogenic variants in biallelic genes are in cis or in trans. Optimal variant interpretation requires a multidisciplinary approach and detailed clinical information, including the prenatal ultrasound scan and family history, should be available to the CRP; the importance of family history was illustrated by the familial *MYCN* variant. After recognising that limited clinical information on the parental phenotype (or incomplete penetrance, or both) might lead to removal of pathogenic inherited developmental disorder gene variants from the dataset (as exemplified by Noonan syndrome variants in *RIT1* and *PTPN11*), we recommend that the bioinformatics pipeline includes strategies to address this issue, such as a whitelist of annotated pathogenic variants and predicted pathogenic (truncating) variants in developmental disorder genes that are not removed from the dataset, even if inherited from an apparently unaffected parent. To maximise the number of diagnostic variants detected, we analysed variants in the 1511 developmental disorder genes included in the developmental disorders gene to phenotype panel and 117 genes identified to be associated with a prenatal presentation from the literature (appendix). This approach resulted in about a third of trios having at least one potential diagnostic finding. When implementing WES into clinical practice, there is a strong argument for curating the developmental disorders gene to phenotype list (by the European Bioinformatics Institute) to remove genes that are not associated with fetal structural anomalies and for using smaller, phenotype-specific virtual gene panels, to reduce the number of VUS that are irrelevant to the fetal structural anomalies. Careful thought is also required to determine which fetal structural anomalies cases should be investigated by WES or WGS. For non-specific fetal structural anomalies that can be associated with a normal or minimal disability outcome (such as talipes equinovarous, resolving ventriculomegaly, or an isolated small nuchal translucency) the number of diagnoses by WES can be small, and interpreting the clinical significance of a VUS can be problematic with a non-specific phenotype.

WES was done at a central sequencing facility that was remote from the clinical centres at which the parents were recruited but virtual meetings of the CRP enabled all relevant, geographically remote specialists (including those who would communicate the results to the parents) to discuss their findings and reach a consensus. Although PAGE results were communicated to women and their partners after the pregnancy, our experience highlighted some potential ethical issues that are relevant for clinical practice. Although many ethical issues (such as incidental findings or non-paternity) are not unique to prenatal WES or WGS analyses, and can be managed according to standard policies, it is essential that parents receive clear information regarding which findings will be reported (eg, a policy is required that indicates whether pathogenic findings in a developmental disorder gene that is not known to be associated with the observed phenotype will be reported to parents, and such a policy should be described when parents give their consent to participate).^18^ These practical ethical issues within the PAGE study illustrate the value of embedded ethics research, and they also highlight the importance of ethics support, training for health professionals, and guidelines for clinical implementation (as were published in 2018 by the International Society of Prenatal Diagnosis).^47^

As prenatal WES becomes more widely adopted, it is crucial that the clinical and molecular data generated is added to a confidential database and is shared widely in an anonymous manner (eg, in DECIPHER or ClinVar), to improve variant interpretation and recognition of novel prenatal genotype-phenotype associations. Such databases should be international to facilitate rapid accumulation of data. Developments in bioinformatic processing could also facilitate better interpretation of variants across different centres.^48^

In conclusion, we report the largest study to date of WES use in a broad range of fetal structural anomalies. Although the proportion of fetuses in which we found diagnostic genetic variants is lower than that suggested by smaller retrospective studies on selected groups, we have found that, in subgroups of fetuses with structural anomalies, adding WES to chromosomal microarray substantially increased the number of fetuses that could be diagnosed with genetic variants that are associated with developmental disorder genes and improved the prognostic information that could be provided for the current and future pregnancies (such as recurrence risks). It seems inevitable that WES will increasingly be applied for investigating fetal structural anomalies, but the PAGE study suggests that this method is best performed by targeting those groups in which it is most likely to be diagnostic (and avoiding situations in which the likely number of diagnoses by this method is low and the detection of VUSs would lead to uncertainty and dilemmas with regard to clinical management). Although WGS might be an alternative to combined chromosomal microarray and WES analysis (and provide added information on non-coding variants) in theory, we expect that chromosomal microarray and WES will be used until there is clear evidence that WGS is superior (for instance, if non-coding variants were to prove to be an important cause of fetal structural anomalies). Finally, we would emphasise the importance of sharing large, carefully curated clinical and genomic datasets to address the challenges of incorporating WES and WGS into prenatal diagnostics.
