# Supplementary material for: Prenatal exome sequencing analysis in fetal structural anomalies detected by ultrasonography (PAGE): a cohort study
Source: Lancet. 2019 Feb 23;393(10173):747–57. doi: 10.1016/S0140-6736(18)31940-8 (PMC6386638; doi:10.1016/S0140-6736(18)31940-8)
Supplement: Supplementary appendix [file mmc1.pdf]

THE LANCET

Supplementary appendix

This appendix formed part of the original submission and has been peer reviewed. We post it as supplied by the authors.

Supplement to: Lord J, McMullan DJ, Eberhardt RY, et al. Reversible encephalopathy caused by an inborn error of cobalamin metabolism. *Lancet* 2019; published online Jan 31. http://dx.doi.org/10.1016/S0140-6736(18)31940-8.

Supplementary Information

Jenny Lord* (1), Dominic J. McMullan* (2), Ruth Y Eberhardt* (1), Gabriele Rinck (1), Susan J. Hamilton (2), Elizabeth Quinlan-Jones (20), Elena Prigmore (1), Rebecca Keelagher (2), Sunayna K Best (3), Georgina K. Carey (2), Rhiannon Mellis (3), Sarah Robart (3), Ian R Berry (4), Kate E. Chandler (5), Deirdre Cilliers (23), Lara Cresswell (6), Sandra L Edwards (7), Carol Gardiner (18) , Alex Henderson (8), Simon T. Holden (9), Tessa Homfray (10), Tracy Lester (24), Rebecca A Lewis (14), Ruth Newbury-Ecob (11), Katrina Prescott (12), Oliver Quarrell (13), Simon Ramsden (5), Eileen Roberts (14), Dagmar Tapon (15), Madeleine J Tooley (11), Pradeep C. Vasudevan (6), Astrid P. Weber (16), Diana G. Wellesley (17), Paul Westwood (18), Helen White (17), Prenatal Assessment of Genomes and Exomes Study (1), Michael Parker (19), Denise Williams (2), Lucy Jenkins (3), Richard H. Scott (3), Mark D. Kilby**+(20,21), Lyn S. Chitty**+(3), Matthew E. Hurles** (1), Eamonn R. Maher**+(9, 22)

*joint first authors

** senior co-authors

+Full professors

1. Wellcome Sanger Institute, Hinxton, Cambridge CB10 1SA, United Kingdom
2. West Midlands Regional Genetics Service, Birmingham Women's and Children's NHS Foundation Trust, Birmingham B15 2TG, UK
3. UCL Great Ormond Street Institute of Child Health and North East Thames Regional Genetics Service, Great Ormond Street NHS Foundation Trust, London UK
4. Yorkshire Regional Genetics Service, The Leeds Genetics Laboratory, St. James's University Hospital, Leeds LS9 7TF, UK
5. Manchester Centre for Genomic Medicine, Manchester University Hospitals NHS Foundation Trust, Manchester Academic Health Science Centre, Manchester, UK

6. University Hospitals of Leicester NHS Trust, Leicester Royal Infirmary, Leicester LE1 5WW, UK
7. Cytogenetics Department, Norfolk & Norwich University Hospital Foundation Trust, Norwich NR4 7UY, UK
8. Northern Genetics Service, Newcastle upon Tyne Hospitals NHS Foundation Trust, UK
9. Department of Clinical Genetics, Cambridge University Hospitals NHS Foundation Trust, Cambridge, CB2 0QQ, UK
10. Southwest Thames Regional Genetics Centre, St George's Healthcare NHS Trust, London SW17 0RE, UK
11. Department of Clinical Genetics, St Michael's Hospital, Bristol BS2 8EG
12. Yorkshire Regional Genetics Service, Chapel Allerton Hospital, Leeds, LS7 4SA, UK
13. Department of Clinical Genetics, Sheffield Children's Hospital, Sheffield UK S10 2TH
14. Bristol Genetics Laboratory, North Bristol NHS Trust, Southmead Hospital, Bristol BS10 5NB, UK
15. Queen Charlotte's and Chelsea Hospital, Imperial College Healthcare NHS Trust, London W12 0HS, UK
16. Department of Clinical Genetics, Liverpool Women's NHS Foundation Trust, Liverpool, UK
17. Faculty of Medicine, University of Southampton and Wessex Clinical Genetics Service, Southampton, United Kingdom
18. West of Scotland Genetics Services, Queen Elizabeth University Hospital, Glasgow G51 4TF, UK
19. Wellcome Centre for Ethics and Humanities and The Ethox Centre, University of Oxford, Oxford, UK
20. West Midlands Fetal Medicine Centre, Birmingham Women's and Children's Foundation Trust, Birmingham B15 2TG, UK.

21. Centre for Women's & Newborn Health, Institute of Metabolism & Systems Research, University of Birmingham, B15 2TT.
22. Department of Medical Genetics, University of Cambridge and NIHR Cambridge Biomedical Research Centre, Cambridge CB2 0QQ, UK
23. Oxford Centre for Genomic Medicine, ACE Building, Nuffield Orthopaedic Centre, Oxford, OX3 7LD, UK
24. Oxford Regional Genetics Services, Oxford University Hospitals, The Churchill Hospital, Oxford, OX3 7LE, UK

Correspondence to: erm1000@medschl.cam.ac.uk

Contents

1. Supplementary Methods
    - Exome sequencing, Variant detection, Annotation and Quality control
2. Supplementary results
    - Variant assessments
3. Supplementary Tables
    - Supplementary Table 1: Previous reports of whole exome sequencing for fetal structural anomalies
    - Supplementary Table 2: Details of developmental disorder gene panel
    - Supplementary Table 3: Classification of fetal structural anomaly phenotypes
    - Supplementary Table 4: Single nucleotide variants (SNVs) and indels reviewed by Clinical Review Panel
    - Supplementary Table 5: Copy number variants (CNVs) and uniparental disomies (UPDs) reviewed by Clinical Review Panel
    - Supplementary Table 6: Fetal outcomes and phenotypic classes
    - Supplementary Table 7: Details of cases with non-diagnostic but potentially clinically relevant variants
    - Supplementary Table 8: Percent of protein coding region covered by >=13X for DDG2P gene panel.
4. Supplementary Figures
    - Supplementary Figure 1: Organisational chart illustrating the role of the clinical review panel in the assessment of genetic variants detected in the PAGE study
    - Supplementary Figure 2: Simplified (omitting quality control steps) overview of the bioinformatics pipeline.
5. Supplementary Case Studies
    - Case 1: *TUBB* variant
    - Case 2: *KMT2D* variant
    - Case 3: *MYCN* variant
6. References for supplementary materials

1. Supplementary Methods

<u>*Exome sequencing, variant detection and annotation:*</u> After receipt of DNA samples at the Wellcome Sanger Institute for WES genomic DNA (~125ng) was fragmented to an average size of 150 base pairs (bp) and DNA libraries were prepared and processed using standard Illumina methodologies. A portion of each library was used to create an equimolar pool comprising 8 indexed libraries. For each pool exome capture was undertaken with the Agilent SureSelect XT Human All Exon V5 Plus with custom ELID#0337431 (Agilent Technologies, Santa Clara, CA, USA) and analysed by 75-base paired-end sequencing (with 6 samples per lane on Illumina HiSeq 2500) following the manufacturer's instructions.

Mapping of short-read sequences was performed with the Burrows-Wheeler aligner (BWA; version 0.59) 41 backtrack algorithm with the GRCh37 1000 Genomes Project phase 2 reference (also known as hs37d5). Single nucleotide variants (SNVs) and indels were identified from CRAM data using the Genome Analysis Toolkit (GATK) HaplotypeCaller version 3.5 (Van der Auwera 2013). DeNovoGear (Ramu et al. 2013) was used to identify candidate *de novo* variants. Copy number variations (CNVs) were detected using CoNVex (http://www.uk10k.org/assets/ashg_vijayarangakannan_etal_2012.pdf), and inheritance predicted with CIFER (https://github.com/jeremymcrae/cifer). Outputs from GATK haplotype caller, DeNovoGear and CoNVex were merged into a single variant call format (VCF) file per trio and annotated with allele frequencies from the Exome Aggregation Consortium (ExAC) (Lek et al. 2016), the 1000 Genomes Project (1000 Genomes Project Consortium 2015), UK10K (UK10K Consortium 2015), the Exome Sequencing Project (ESP) (Exome Variant Server, NHLBI GO Exome Sequencing Project (ESP), Seattle, WA (URL: http://evs.gs.washington.edu/EVS/) (accessed Jan 2013), and from unaffected parents from the Deciphering Developmental Disorders (DDD) and PAGE studies. Ensembl's Variant Effect Predictor (VEP version 85) (McLaren et al. 2016) was used to annotate the predicted effect of each variant. UPD was identified using UPDio (King et al. 2014). The bioinformatics pathway is summarised in supplementary Figure 2.

Quality control (QC) was performed on all VCF files to exclude samples where exome sequencing was of poor quality and to exclude potentially contaminated samples. Upon receipt of samples at the Wellcome Sanger Institute, a Fluidigm genotyping panel of 57 SNVs was run, allowing verification of fetal sex and family structure. Once VCF files were generated, these Fluidigm variants were used as a QC measure, with any samples in which a <80% match to Fluidigm genotypes was observed across at least 14 called sites failing QC. Any samples with <80,000 total variants failed QC. QC

failures had exome sequencing repeated. If a fetal sample failed, analysis of the trio was delayed until new sequencing data was available. If a parental sample failed, the family was analysed as a dyad until the additional parent's data became available, when trio analysis could be conducted.

A number of additional QC metrics were checked: total reads in CRAM file, total homozygous and heterozygous calls (SNVs and indels), proportion of variant calls that were common, number of variants falling into different VEP annotated consequence categories, number of extreme heterozygotes ((alternate allele proportion <0.15 or >0.8), as these can be indicative of sample contamination), kinship (King (Manichaikul et al. 2010) and PCrelate (Conomos et al. 2016)) and autozygosity (BCFtools/RoH; Narasimhan et al 2016)), and outliers were flagged but not necessarily repeat sequenced. Autosomal variants with genotype quality (GQ) <50 were validated in house before reporting to clinical review panel/team (CRP). When calling *de novo* mutations (DNMs), samples with excess (>50) high quality DNMs (pp_dnm >0.9 and MAF <0.01) were flagged, and any DNMs with pp_dnm <0.1 were validated in house before reporting to CRP. For CNVs, total CNVs per individual were plotted against the fraction overlapping common CNVs, and quality metrics from CoNVex (mean and MADs) were plotted, allowing the identification of outliers. Any deletions/duplications supported by ≤3 probes (which have high false positive rates) were validated in house before reporting to CRP.

Variants were filtered to identify those of potential clinical significance. Protein-altering variants were selected using VEP annotation and common variants were excluded using minor allele frequencies. A developmental disorder-associated gene list (www.ebi.ac.uk/gene2phenotype; downloaded in February 2018) (Wright et al. 2015) was modified to exclude genes without a prenatal phenotype, and supplemented with 117 genes identified from the literature and deemed to be robustly associated with a prenatal phenotype (Supplementary Table 2). Rare, protein altering variants in genes in this list where the inheritance pattern of the variant matches that of the gene were selected for clinical review.

Filtering by MAF was a two-step process. Firstly variants were filtered using ExAC, 1000 genomes, UK10K, ESP and DDD unaffected parents. All variants with a MAF of > 0.005 were excluded, biallelic variants and those with multiple inheritance types beneath this threshold pass the MAF filter. More stringent filters were used for other variants depending on the availability of parent data: if both parents were present a threshold of 0.0005 was used, and if data for one or both parents was missing a threshold of 0.0001 was used. These thresholds are in line with those used in the DDD study. In addition we filtered on a MAF from PAGE unaffected parents. Because of the size of the PAGE dataset the maximum value of this MAF when a variant was only observed in one unaffected

parent was 0.000463, therefore the above thresholds were too stringent and a maximum allele frequency of 0.01 was used.

During the course of the study, we became aware of a missed diagnosis (a *RIT1* mutation) which failed bioinformatic filtering because it was inherited from an apparently unaffected parent. However, the variant was a known disease causing mutation, and should not have been overlooked. We implemented a “whitelist” strategy, developed by Panayiotis Constantinou for the DDD project. We established a list of ClinVar variants with 2* (criteria provided, multiple submitters with no conflict) or 3* (reviewed by expert panel) status and reviewed these variants within the PAGE data, regardless of inheritance and parental affected status.

2. Supplementary Results

Variant Assessments: The 321 genetic variants, representing 255 potential diagnoses in 205 individuals that satisfied the filtering criteria and were reviewed by the clinical review panel (CRP) are detailed in Supplementary Table 4). In summary these variants consisted 301 SNV/indel variants in 192 probands, 18 CNVs and two instances of UPDs. Of the 301 SNVs/indels, 102 were in autosomal monoallelic disease genes, 151 in autosomal biallelic disease genes (26 homozygous variants, and 125 variants in compound heterozygous state, giving 61 potential diagnoses, including two individuals with a CNV affecting one allele and a missense variant the other, and five instances where three variants meeting filtering criteria were observed in a biallelic gene in a single individual), and 48 were in genes on the X-chromosome.

Of the 301 SNV/indel variants reviewed, 47 were protein truncating variants (15 stop gained, 8 essential splice site and 24 frameshift; 19 DNMs, 14 inherited variants as part of compound heterozygous events, 9 biallelic inherited homozygous variants, 1 inherited variant in an imprinted gene, 1 inherited from a potentially similarly affected parent, 1 X-linked inherited hemizygous variant in a male fetus, and 2 of uncertain inheritance), 12 were inframe insertions or deletions (2 DNMs, 5 inherited variants in compound heterozygous state, 3 X-linked inherited variants in hemizygous state, and 2 of uncertain inheritance), and 242 were missense variants (59 DNMs, 106 inherited variants in compound heterozygous state, 17 biallelic inherited homozygous variants, 6 variants inherited from potentially similarly affected parents, 3 variants inherited from apparently unaffected parents, 36 inherited X-linked variants in hemizygous state, and 15 of uncertain inheritance).

1. Supplementary Tables

Supplementary Table 1: Summary of previously published WES studies for fetal anomalies. Genes in bold were also identified as harbouring diagnostic variants in the current cohort. Genes underlined have been reported to contain diagnostic variants in multiple previous publications but not in the PAGE cohort.

Study	Subjects	Diagnostic rate	Comments	Genes considered to have diagnostic variants
Yates et al (2017)	84	20%	Diagnostic rate for trio analysis 24%. Fetuses selected for USS anomalies that were terminated or resulted in fetal demise.	*AMER1, BBS4, CYP11A1, FANCB, FGFR2, FLNA, FOXP3, L1CAM, HRAS, MRPS22,* ***PIEZO1****,* ***PIK3CA****, PIK3R2,* ***PTPN11****, RIPK4,* ***RIT1****,* ***SOX9***
Vora et al. (2017)	15	47%	Recruitment criteria included “a pattern of anomalies highly suggestive of an underlying genetic disorder” and “diagnostic rate” includes possible diagnoses	***COL1A1****,* ***DYNC2H1****, KCTD1, MUSK,* ***PIEZO1****, RTTN, TMEM67*
Pangalos et al. (2016)	14	43%	In 3 cases diagnosis was aided by testing of other affected relatives/fetuses	*ASS1, COL3A1,* ***EVC2****, NEB,* ***PTPN11****, PROKR2*
Alamillo et al. (2015)	7	57%	3 cases categorised as ‘positive’ results and one as ‘likely positive’.	*COL1A2, GBE1, OFD1,* ***RAPSN***
Westerfield et al. (2015)	10	30%		***NIPBL****, TMEM67, WDR19*
Drury et al. (2015)	24	21%	14 cases WES performed on fetal DNA only, 10 cases trio sequenced.	*ACTB, ALPL, COL2A1, FLT4, MYH3*
Carss et al. (2014)	30	10%	A further 5 (17%) cases had potentially pathogenic variants requiring further investigation	*COL2A1, FGFR3, OFD1*
Yang et al. (2014)	11	55%		*ALG12, DOK7,* ***KMT2D****, PEX1 PEX12, TMEM67*
Yang et al. (2013)	4	25%		***NIPBL***

Supplementary Table 2: Variants were analysed in the 1511 developmental disorder genes included in the DDG2P panel (www.ebi.ac.uk/gene2phenotype) (downloaded February 2018), plus 117 genes identified as being associated with a prenatal presentation from the literature (see below)

*pLI = probability of loss of function intolerance

**No Ensembl protein coding transcript in GRCh37

Gene name	Gene MIM	Expected mode	Expected mechanism	pLI*	Percent of protein coding region covered by >=13X
ABCA12	607800	Biallelic	Loss of function	0.0000	98.37
ABCC8	600509	Monoallelic	uncertain	0.0000	99.98
ACE	106180	Biallelic	Loss of function	0.0000	100.00
ACTG2	102545	Monoallelic	Uncertain	0.6920	97.79
ADAMTS10	608990	Biallelic	Loss of function	0.9803	100.00
ADAMTS17	607511	Biallelic	Loss of function	0.0000	100.00
ADAMTSL2	612277	Biallelic	Loss of function	0.0711	92.70
AGPAT2	603100	Biallelic	Loss of function	0.0002	94.50
AGRN	103320	Biallelic	Loss of function	0.1734	92.38
ANOS1	300836	Hemizygous	Loss of function	0.9386	87.20
AP3B1	603401	Biallelic	Loss of function	0.9954	95.83
ARL13B	608922	Biallelic	Loss of function	0.0000	100.00

ASNS	108370	Biallelic	All missense/in frame	0.0017	89.79
ATP6V0A2	611716	Biallelic	Loss of function	0.0000	100.00
B3GAT3	606374	Biallelic	Loss of function	0.0003	97.72
B3GLCT	606374	Biallelic	Loss of function	0.0000	99.40
CAVIN1	603198	Biallelic	Loss of function	0.0209	100.00
CDAN1	607465	Biallelic	Loss of function	0.9281	100.00
CEP164	614848	Biallelic	Loss of function	0.0000	97.38
CFTR	602421	Biallelic	Loss of function	0.0000	97.28
CHAT	118490	Biallelic	Loss of function	0.0140	100.00
CHKB	612395	Biallelic	Loss of function	0.0006	100.00
CHRND	100720	Biallelic	Loss of function	0.0000	100.00
COL1A2	120160	Biallelic/Monoallelic	Loss of function	0.9998	100.00
COL5A1	120215	Monoallelic	Loss of function	1.0000	99.23
COL5A2	120190	Monoallelic	Loss of function	1.0000	100.00
COL6A2	120240	Biallelic/Monoallelic	Loss of function	0.0021	100.00
CPT2	600650	Biallelic	Loss of function	0.0000	98.79

CRLF1	604237	Biallelic	Loss of function	0.0269	91.77
CRTAP	605497	Biallelic	Loss of function	0.0001	99.32
CYP11A1	118485	Biallelic	Loss of function	0.0000	92.90
CYP11B1	610613	Biallelic/Monoallelic	Loss of function	0.0014	100.00
CYP17A1	609300	Biallelic	Loss of function	0.0350	100.00
CYP21A2	613815	Biallelic	Loss of function	0.6723	87.30
DNAAF1	613190	Biallelic	Loss of function	0.0000	99.27
DNAH11	603339	Biallelic	Loss of function	NA	100.00
DNAH5	603335	Biallelic	Loss of function	0.0000	100.00
DNAI1	604366	Biallelic	Loss of function	0.0001	100.00
DSP	125647	Biallelic/Monoallelic	uncertain	0.9999	99.82
EMD	300384	Hemizygous	Loss of function	0.8444	100.00
EMG1	611531	Biallelic	All missense/in frame	0.0106	100.00
EPHB4	600011	Monoallelic	Loss of function	0.9899	100.00
FBLN5	604580	Biallelic	All missense/in frame	0.9958	99.63
FGF8	600483	Monoallelic	uncertain	0.9272	89.58

G6PC3	611045	Biallelic	Loss of function	0.0000	100.00
GBE1	607839	Biallelic	Loss of function	0.0000	100.00
GPI	172400	Biallelic	All missense/in frame	0.0016	100.00
GRIP1	604597	Biallelic	Loss of function	0.9169	100.00
H19	103280	Monoallelic	uncertain	NA	No protein**
HES7	608059	Biallelic	Loss of function	0.7829	81.39
HSD17B3	605573	Biallelic	Loss of function	0.0000	100.00
IER3IP1	609382	Biallelic	Loss of function	0.0184	77.51
INSR	147670	Biallelic	Loss of function	0.1865	100.00
INVS	243305	Biallelic	Loss of function	0.0000	100.00
IQCB1	609237	Biallelic	Loss of function	0.0000	76.67
ITGA6	147556	Biallelic	Loss of function	0.0099	100.00
ITGB4	147557	Biallelic	Loss of function	0.0000	94.32
KCNJ1	600359	Biallelic	Loss of function	0.0017	100.00
KCNJ2	600681	Monoallelic	Loss of function	0.8202	100.00
KISS1R	604161	Biallelic	Loss of function	0.0040	100.00

KLHL41	607701	Biallelic	Loss of function	0.0188	100.00
LIFR	151443	Biallelic	Loss of function	0.0010	99.12
LMBR1	605522	Biallelic/Monoallelic	Loss of function	0.0007	100.00
LMOD3	616112	Biallelic	Loss of function	0.0081	99.47
LTBP4	604710	Biallelic	Loss of function	0.9904	100.00
LZTFL1	606568	Biallelic	Loss of function	0.1941	100.00
MBTPS2	300294	Hemizygous	Loss of function	0.9402	98.40
MLH1	120436	Biallelic/Monoallelic	Loss of function	0.7396	99.96
MSH2	609309	Biallelic/Monoallelic	Loss of function	0.8677	97.29
MSH6	600678	Biallelic/Monoallelic	Loss of function	NA	100.00
MUSK	601296	Biallelic	Loss of function	0.0001	100.00
MYBPC1	160794	Biallelic/Monoallelic	Loss of function	0.6579	100.00
NHEJ1	611290	Biallelic	Loss of function	0.0272	91.56
NR0B1	300473	X-linked dominant	Loss of function	0.7920	100.00
OCLN	602876	Biallelic	Loss of function	0.4910	100.00
OSTM1	607649	Biallelic	Loss of function	0.0605	90.25

PIEZO1	611184	Biallelic/Monoallelic	Loss of function	0.5360	97.71
PKD1	601313	Monoallelic	Loss of function	0.9999	31.19
PKD2	173910	Monoallelic	Loss of function	0.9953	96.61
PKLR	609712	Biallelic	Loss of function	0.0000	98.96
POMK	615247	Biallelic	Loss of function	0.0005	100.00
POR	124015	Biallelic	Loss of function	0.0000	98.83
PPIB	123841	Biallelic	Loss of function	0.0107	100.00
PRG4	604283	Biallelic	Loss of function	0.4693	100.00
PROK2	607002	Biallelic/Monoallelic	Loss of function	0.2705	100.00
PROKR2	607123	Monoallelic	uncertain	0.0007	100.00
REN	179820	Biallelic	Loss of function	0.0066	100.00
ROBO1	602430	Monoallelic	Loss of function	0.0000	100.00
RPL11	604175	Monoallelic	Loss of function	0.7258	95.53
RPL5	603634	Monoallelic	Loss of function	0.9948	57.05
RPS10	603632	Monoallelic	Loss of function	0.9465	88.96
RPS17	180472	Monoallelic	Loss of function	NA	55.88

RPS26	603701	Monoallelic	Loss of function	0.7533	76.72
SELENON	606210	Biallelic	Loss of function	0.0000	83.70
SGCA	600119	Biallelic	Loss of function	0.1944	100.00
SLC12A1	600839	Biallelic	Loss of function	0.0000	100.00
SLC26A3	126650	Biallelic	Loss of function	0.0000	100.00
SMN1	600354	Biallelic	Loss of function	0.1010	93.33
SOST	605740	Biallelic	Loss of function	0.3796	98.91
SP110	604457	Biallelic	Loss of function	0.0000	100.00
SRD5A2	607306	Biallelic	Loss of function	NA	No protein**
SUFU	607035	Monoallelic	Loss of function	0.9992	98.79
TBC1D20	611663	Biallelic	Loss of function	0.9126	99.91
TBX6	602427	Monoallelic	Loss of function	0.0291	97.33
TCIRG1	604592	Biallelic	Loss of function	0.0000	91.18
TCTN1	609863	Biallelic	Loss of function	0.0000	97.04
TGIF1	602630	Monoallelic	Loss of function	0.0908	100.00
TGM1	190195	Biallelic	Loss of function	0.0000	100.00

TMEM138	614459	Biallelic	Loss of function	0.5311	100.00
TMEM231	614949	Biallelic	Loss of function	0.0001	97.69
TNNI2	191043	Monoallelic	Loss of function	0.0862	100.00
TNNT1	191041	Biallelic	Loss of function	0.0000	98.91
TNXB	600985	Biallelic/Monoallelic	Loss of function	0.7742	90.01
TPM3	191030	Biallelic/Monoallelic	Loss of function	0.1920	98.25
TTC21B	612014	Biallelic	Loss of function	0.0000	95.06
UBA1	314370	Hemizygous	Loss of function	1.0000	100.00
ZNF423	604557	Biallelic/Monoallelic	Loss of function	0.9905	100.00

Supplementary Table 3: Classification of fetal structural anomaly phenotypes

**Abdominal**	**Omphalocoele, gastroschisis, echogenic bowel, bowel obstruction**
**Brain**	**Ventriculomegaly (mild persistent and resolving), posterior fossa cysts, other major CNS anomalies**
**Cardiac**	**Any cardiac malformation or variant**
**Chest**	**Cystic lung lesions, diaphragmatic hernia**
**Facial**	**Cleft lip +/- palate**
**Hydrops**	**Pleural effusions, ascites, generalised skin oedema**
**Isolated NT >4mm**	**Increased nuchal translucency (NT)**
**Multisystem**	**All cases with more than one structural anomaly**
**Renal**	**Echogenic or absent kidneys, hydronephrosis, outflow obstruction**
**Skeletal**	**Talipes, transverse limb defects, radial ray defects, contractures, suspected skeletal dysplasias**
**Spinal**	**Hemivertebrae, spinabifida**

Supplementary Table 4: Single nucleotide variants (SNVs) and indels reviewed by Clinical Review Panel (hg19 coordinates)

EGA_ID	PP_ID	Phenotypic Class	Fetal sex	Chr	Pos	Ref	Alt	Gene	Variant Type	Inheritance	Zygosity	Review Outcome
EGAN00001366413	PP0087	Skeletal	F	11	103057046	C	T	DYNC2H1	stop_gained	Inherited	Compound heterozygous	Diagnostic
EGAN00001366413	PP0087	Skeletal	F	11	103060543	C	T	DYNC2H1	stop_gained	Inherited	Compound heterozygous	Diagnostic
EGAN00001366649	PP0099	Spinal	M	10	73567140	C	T	CDH23	missense_variant	Inherited	Compound heterozygous	Not relevant
EGAN00001366649	PP0099	Spinal	M	10	73468926	C	T	CDH23	missense_variant	Inherited	Compound heterozygous	Not relevant
EGAN00001366670	PP0120	Skeletal	M	1	5964799	C	A	NPHP4	missense_variant	Inherited	Compound heterozygous	Not relevant
EGAN00001366670	PP0120	Skeletal	M	1	5937203	G	A	NPHP4	missense_variant	Inherited	Compound heterozygous	Not relevant
EGAN00001366679	PP0129	Skeletal	M	X	32456380	C	T	DMD	missense_variant	Inherited from unaffected mother (X)	Hemizygous	Not relevant
EGAN00001366679	PP0129	Skeletal	M	X	153589917	T	C	FLNA	missense_variant	Inherited from unaffected mother (X)	Hemizygous	Potentially clinically useful
EGAN00001366705	PP0156	Hydrops	M	19	50831536	C	A	KCNC3	missense_variant	Uncertain (dyad)	Heterozygous	Not relevant
EGAN00001366705	PP0156	Hydrops	M	2	50758415	G	A	NRXN1	missense_variant	Uncertain (dyad)	Heterozygous	Not relevant
EGAN00001366705	PP0156	Hydrops	M	6	137193340	C	T	PEX7	missense_variant	Uncertain (dyad)	Heterozygous	Not relevant
EGAN00001366705	PP0156	Hydrops	M	6	137193333	TAG	T	PEX7	splice_acceptor_variant	Uncertain (dyad)	Heterozygous	Not relevant

EGAN00001366705	PP0156	Hydrops	M	3	114070407	A	G	ZBTB20	missense_variant	Uncertain	Heterozygous	Not relevant
EGAN00001366711	PP0162	Hydrops	F	6	51798959	G	A	PKHD1	missense_variant	Inherited	Compound heterozygous	Not relevant
EGAN00001366711	PP0162	Hydrops	F	6	51524453	T	G	PKHD1	missense_variant	Inherited	Compound heterozygous	Not relevant
EGAN00001366717	PP0168	Cardiac	M	15	57544664	A	G	TCF12	missense_variant	de novo	Heterozygous	Not relevant
EGAN00001366723	PP0174	Multisystem	F	1	115258748	C	G	NRAS	missense_variant	de novo	Heterozygous	Diagnostic
EGAN00001366733	PP0184	Cardiac	F	15	96877607	T	C	NR2F2	missense_variant	de novo	Heterozygous	Diagnostic
EGAN00001366736	PP0187	Brain	M	X	12720120	A	G	FRMPD4	missense_variant	Inherited from unaffected mother (X)	Hemizygous	Not relevant
EGAN00001366736	PP0187	Brain	M	X	153295988	T	C	MECP2	missense_variant	Inherited from unaffected mother (X)	Hemizygous	Not relevant
EGAN00001366768	PP0204	Skeletal	F	X	64139032	T	TG	ZC4H2	frameshift_variant	de novo	Heterozygous	Diagnostic
EGAN00001366769	PP0205	Large NT>4.0	F	4	79399020	G	A	FRAS1	missense_variant	Inherited	Compound heterozygous	Not relevant
EGAN00001366769	PP0205	Large NT>4.0	F	4	79443878	T	C	FRAS1	missense_variant	Inherited	Compound heterozygous	Not relevant
EGAN00001366774	PP0207	Renal	M	6	143095041	T	C	HIVEP2	missense_variant	de novo	Heterozygous	Not relevant
EGAN00001366776	PP0208	Skeletal	F	15	48713770	C	T	FBN1	missense_variant	de novo	Heterozygous	Not relevant
EGAN00001366757	PP0234	Large NT>4.0	F	X	41333998	C	A	NYX	missense_variant	de novo	Heterozygous	Not relevant

EGAN00001366802	PP0258	Abdominal	F	2	16082533	T	C	MYCN	missense_variant	Inherited from affected parent	Heterozygous	Diagnostic
EGAN00001366802	PP0258	Abdominal	F	4	88957380	G	A	PKD2	missense_variant	Inherited from affected parent	Heterozygous	Not relevant
EGAN00001366808	PP0261	Large NT>4.0	M	1	154246309	C	T	HAX1	missense_variant	Inherited	Compound heterozygous (with CNV)	Not relevant
EGAN00001366828	PP0279	Chest	M	X	85213969	C	T	CHM	missense_variant	Inherited from unaffected mother (X)	Hemizygous	Not relevant
EGAN00001366834	PP0285	Large NT>4.0	M	17	41243509	T	C	BRCA1	missense_variant	Inherited	Compound heterozygous	Not relevant
EGAN00001366834	PP0285	Large NT>4.0	M	17	41245233	A	G	BRCA1	missense_variant	Inherited	Compound heterozygous	Not relevant
EGAN00001366834	PP0285	Large NT>4.0	M	2	238274569	G	T	COL6A3	missense_variant	Inherited	Compound heterozygous	Not relevant
EGAN00001366834	PP0285	Large NT>4.0	M	2	238275519	C	T	COL6A3	missense_variant	Inherited	Compound heterozygous	Not relevant
EGAN00001366849	PP0300	Facial/cleft lip±palate	M	X	53276312	C	T	IQSEC2	missense_variant	Inherited from unaffected mother (X)	Hemizygous	Not relevant
EGAN00001366852	PP0303	Cardiac	M	2	175614789	T	C	CHRNA1	missense_variant	Inherited	Compound heterozygous	Not relevant
EGAN00001366852	PP0303	Cardiac	M	2	175619069	G	T	CHRNA1	missense_variant	Inherited	Compound heterozygous	Not relevant
EGAN00001366852	PP0303	Cardiac	M	X	64137700	C	T	ZC4H2	missense_variant	Inherited from unaffected mother (X)	Hemizygous	Not relevant
EGAN00001366861	PP0312	Large NT>4.0	F	21	46895431	G	C	COL18A1	missense_variant	Inherited	Homozygous	Not relevant
EGAN0000136668	PP0312	Large NT>4.0	F	14	58923476	A	T	KIAA0586	missense_variant	Inherited	Homozygous	Potentially clinically

61												useful
EGAN00001366861	PP0312	Large NT>4.0	F	8	6302487	A	C	MCPH1	missense_variant	Inherited	Homozygous	Not relevant
EGAN00001366861	PP0312	Large NT>4.0	F	17	78187976	C	T	SGSH	missense_variant	Inherited	Homozygous	Not relevant
EGAN00001366864	PP0315	Multisystem	F	1	103548462	G	A	COL11A1	missense_variant	Inherited	Compound heterozygous (with CNV)	Not relevant
EGAN00001366864	PP0315	Multisystem	F	14	74711975	C	T	VSX2	missense_variant	Inherited	Homozygous	Not relevant
EGAN00001366867	PP0318	Skeletal	F	2	233406191	C	CA	CHRNG	frameshift_variant	Inherited	Homozygous	Diagnostic
EGAN00001366892	PP0330	Cardiac	M	3	11059010	C	T	SLC6A1	missense_variant	de novo	Heterozygous	Not relevant
EGAN00001366934	PP0333	Cardiac	M	8	11566037	TG	T	GATA4	frameshift_variant	de novo (presumed - (dyad)	Heterozygous	Diagnostic
EGAN00001366953	PP0342	Multisystem	F	2	233408374	CCA	C	CHRNG	frameshift_variant	Inherited	Compound heterozygous	Diagnostic
EGAN00001366953	PP0342	Multisystem	F	2	233406191	C	CA	CHRNG	frameshift_variant	Inherited	Compound heterozygous	Diagnostic
EGAN00001366985	PP0360	Skeletal	M	X	73960674	C	T	KIAA2022	missense_variant	Inherited from unaffected mother (X)	Hemizygous	Not relevant
EGAN00001366985	PP0360	Skeletal	M	18	7032153	C	T	LAMA1	missense_variant	Inherited	Compound heterozygous	Not relevant
EGAN00001366985	PP0360	Skeletal	M	18	7008517	A	T	LAMA1	stop_gained	Inherited	Compound heterozygous	Not relevant
EGAN00001366985	PP0360	Skeletal	M	17	62026805	C	A	SCN4A	missense_variant	de novo	Heterozygous	Not relevant
EGAN00001366996	PP0366	Chest	M	14	92442496	C	A	TRIP11	missense_variant	Inherited	Compound heterozygous	Not relevant

EGAN00001366996	PP0366	Chest	M	14	92477364	A	G	TRIP11	missense_variant	Inherited	Compound heterozygous	Not relevant
EGAN00001367002	PP0369	Renal	M	X	153581143	G	A	FLNA	missense_variant	Inherited from unaffected mother (X)	Hemizygous	Potentially clinically useful
EGAN00001367002	PP0369	Renal	M	2	44190789	T	C	LRPPRC	missense_variant	Inherited	Compound heterozygous	Not relevant
EGAN00001367002	PP0369	Renal	M	2	44223023	G	C	LRPPRC	missense_variant	Inherited	Compound heterozygous	Not relevant
EGAN00001367008	PP0372	Large NT>4.0	F	3	53783158	G	A	CACNA1D	missense_variant	Inherited	Compound heterozygous	Not relevant
EGAN00001367008	PP0372	Large NT>4.0	F	3	53842691	CCTT	C	CACNA1D	inframe_deletion	Inherited	Compound heterozygous	Not relevant
EGAN00001367033	PP0384	Brain	F	13	31843415	G	A	B3GLCT	splice_donor_variant	Inherited	Homozygous	Diagnostic
EGAN00001367033	PP0384	Brain	F	1	22170712	C	A	HSPG2	missense_variant	Inherited	Compound heterozygous	Not relevant
EGAN00001367033	PP0384	Brain	F	1	22174345	C	T	HSPG2	missense_variant	Inherited	Compound heterozygous	Not relevant
EGAN00001367043	PP0390	Cardiac	M	17	42979917	A	C	CCDC103	missense_variant	Inherited	Homozygous	Diagnostic
EGAN00001366872	PP0413	Chest	F	17	79477837	T	C	ACTG1	missense_variant	Inherited from affected parent	Heterozygous	Not relevant
EGAN00001366872	PP0413	Chest	F	7	94038703	C	T	COL1A2	missense_variant	Inherited from affected parent	Heterozygous	Not relevant
EGAN00001366883	PP0419	Skeletal	F	16	51171424	G	A	SALL1	stop_gained	de novo	Heterozygous	Not relevant
EGAN00001366931	PP0425	Cardiac	F	1	22165358	C	T	HSPG2	missense_variant	Inherited	Compound heterozygous	Not relevant

EGAN00001366931	PP0425	Cardiac	F	1	22156041	C	T	HSPG2	missense_variant	Inherited	Compound heterozygous	Not relevant
EGAN00001366931	PP0425	Cardiac	F	16	70902514	C	T	HYDIN	missense_variant	Inherited	Compound heterozygous	Potentially clinically useful
EGAN00001366931	PP0425	Cardiac	F	16	71004560	G	T	HYDIN	missense_variant	Inherited	Compound heterozygous	Potentially clinically useful
EGAN00001366931	PP0425	Cardiac	F	9	131395136	G	A	SPTAN1	missense_variant	de novo	Heterozygous	Not relevant
EGAN00001366484	PP0434	Cardiac	M	14	30103649	A	G	PRKD1	missense_variant	de novo	Heterozygous	Not relevant
EGAN00001366968	PP0443	Large NT>4.0	M	2	152484327	G	A	NEB	missense_variant	Inherited	Compound heterozygous	Not relevant
EGAN00001366968	PP0443	Large NT>4.0	M	2	152524388	T	C	NEB	missense_variant	Inherited	Compound heterozygous	Not relevant
EGAN00001367048	PP0482	Multisystem	M	X	152956861	C	T	SLC6A8	missense_variant	Inherited from unaffected mother (X)	Hemizygous	Not relevant
EGAN00001367098	PP0513	Cardiac	F	7	21938979	C	T	DNAH11	stop_gained	Inherited	Homozygous	Diagnostic
EGAN00001367101	PP0516	Cardiac	M	11	73834139	G	A	C2CD3	missense_variant	Inherited	Compound heterozygous	Not relevant
EGAN00001367101	PP0516	Cardiac	M	11	73879506	A	C	C2CD3	missense_variant	Inherited	Compound heterozygous	Not relevant
EGAN00001367101	PP0516	Cardiac	M	7	158672528	G	C	WDR60	missense_variant	Inherited	Compound heterozygous	Not relevant
EGAN00001367101	PP0516	Cardiac	M	7	158663939	A	G	WDR60	missense_variant	Inherited	Compound heterozygous	Not relevant
EGAN00001367101	PP0516	Cardiac	M	X	128940428	G	A	ZDHHC9	missense_variant	Inherited from unaffected mother (X)	Hemizygous	Not relevant

EGAN00001367110	PP0525	Hydrops	F	19	17088279	T	C	CPAMD8	missense_variant	Inherited	Compound heterozygous	Not relevant
EGAN00001367110	PP0525	Hydrops	F	19	17008607	C	G	CPAMD8	missense_variant	Inherited	Compound heterozygous	Not relevant
EGAN00001367140	PP0555	Multisystem	M	4	5567006	C	CAA	EVC2	frameshift_variant	Inherited	Homozygous	Diagnostic
EGAN00001367140	PP0555	Multisystem	M	3	136002730	C	T	PCCB	missense_variant	Inherited	Homozygous	Not relevant
EGAN00001367146	PP0561	Brain	M	X	23411544	AATG	A	PTCHD1	inframe_deletion	Inherited from unaffected mother (X)	Hemizygous	Not relevant
EGAN00001367158	PP0573	Multisystem	F	17	18058468	G	A	MYO15A	missense_variant	Inherited	Compound heterozygous	Not relevant
EGAN00001367158	PP0573	Multisystem	F	17	18067119	A	G	MYO15A	missense_variant	Inherited	Compound heterozygous	Not relevant
EGAN00001367161	PP0576	Skeletal	F	5	1268700	C	T	TERT	missense_variant	Inherited	Compound heterozygous	Not relevant
EGAN00001367161	PP0576	Skeletal	F	5	1268697	C	T	TERT	missense_variant	Inherited	Compound heterozygous	Not relevant
EGAN00001367167	PP0582	Facial/cleft lip±palate	F	15	99452099	ACAC	A	IGF1R	inframe_deletion	de novo	Heterozygous	Not relevant
EGAN00001367170	PP0585	Large NT>4.0	F	3	78711245	CT	C	ROBO1	frameshift_variant	de novo	Heterozygous	Potentially clinically useful
EGAN00001366460	PP0620	Large NT>4.0	M	1	17312607	G	A	ATP13A2	missense_variant	Inherited	Compound heterozygous	Not relevant
EGAN00001366460	PP0620	Large NT>4.0	M	1	17313007	A	C	ATP13A2	missense_variant	Inherited	Compound heterozygous	Not relevant
EGAN00001366467	PP0623	Multisystem	F	11	125889553	G	A	CDON	missense_variant	de novo	Heterozygous	Not relevant
EGAN0000013664	PP0626	Multisystem	M	20	9459569	C	G	PLCB4	missense_variant	de novo	Heterozygous	Not relevant

73												
EGAN00001366491	PP0635	Brain	M	1	8525998	G	T	RERE	missense_variant	Uncertain (dyad)	Heterozygous	Not relevant
EGAN00001366515	PP0659	Multisystem	M	11	47460282	C	G	RAPSN	splice_donor_variant	Inherited	Homozygous	Diagnostic
EGAN00001366524	PP0668	Large NT>4.0	M	X	53652753	G	GGGGGCC	HUWE1	inframe_insertion	Inherited from unaffected mother (X)	Hemizygous	Not relevant
EGAN00001366452	PP0707	Large NT>4.0	M	X	152991283	G	A	ABCD1	missense_variant	Inherited from unaffected mother (X)	Hemizygous	Not relevant
EGAN00001366458	PP0710	Multisystem	F	1	27094375	T	C	ARID1A	missense_variant	de novo	Heterozygous	Not relevant
EGAN00001366470	PP0716	Cardiac	M	X	39913562	C	T	BCOR	missense_variant	Inherited from unaffected mother (X)	Hemizygous	Not relevant
EGAN00001366482	PP0722	Large NT>4.0	F	12	49420214	G	A	KMT2D	missense_variant	de novo	Heterozygous	Potentially clinically useful
EGAN00001366552	PP0792	Skeletal	F	17	48264464	C	T	COL1A1	missense_variant	de novo	Heterozygous	Diagnostic
EGAN00001402388	PP0969	Abdominal	F	18	24128418	T	C	KCTD1	missense_variant	de novo	Heterozygous	Not relevant
EGAN00001543707	PP0981	Multisystem	F	1	155209416	AG	A	GBA	frameshift_variant	Inherited	Homozygous	Diagnostic
EGAN00001402421	PP0984	Multisystem	M	9	123171414	TC	AT	CDK5RAP2	missense_variant	Inherited	Homozygous	Not relevant
EGAN00001402421	PP0984	Multisystem	M	6	152690195	A	G	SYNE1	missense_variant	Inherited	Homozygous	Not relevant
EGAN00001402433	PP1038	Large NT>4.0	M	5	13719123	G	A	DNAH5	missense_variant	Inherited	Compound heterozygous	Not relevant

EGAN00001402433	PP1038	Large NT>4.0	M	5	13727650	A	G	DNAH5	missense_variant	Inherited	Compound heterozygous	Not relevant
EGAN00001543736	PP1206	Multisystem	F	6	33139522	G	T	COL11A2	missense_variant	Uncertain (dyad)	Heterozygous	Not relevant
EGAN00001543736	PP1206	Multisystem	F	X	153577265	C	G	FLNA	missense_variant	Uncertain (dyad)	Heterozygous	Not relevant
EGAN00001366633	PP1323	Cardiac	M	X	74376080	G	A	ABCB7	missense_variant	Inherited from unaffected mother (X)	Hemizygous	Not relevant
EGAN00001366546	PP1324	Skeletal	M	12	49579680	G	A	TUBA1A	missense_variant	de novo	Heterozygous	Potentially clinically useful
EGAN00001366555	PP1327	Abdominal	M	1	100349757	A	G	AGL	missense_variant	Inherited	Homozygous	Not relevant
EGAN00001366555	PP1327	Abdominal	M	10	73442306	G	A	CDH23	missense_variant	Inherited	Compound heterozygous	Not relevant
EGAN00001366555	PP1327	Abdominal	M	10	73437293	C	T	CDH23	missense_variant	Inherited	Compound heterozygous	Not relevant
EGAN00001366564	PP1336	Large NT>4.0	M	2	238277376	T	C	COL6A3	missense_variant	Inherited	Compound heterozygous	Not relevant
EGAN00001366564	PP1336	Large NT>4.0	M	2	238283241	T	C	COL6A3	missense_variant	Inherited	Compound heterozygous	Not relevant
EGAN00001366564	PP1336	Large NT>4.0	M	2	238296306	G	C	COL6A3	missense_variant	Inherited	Compound heterozygous	Not relevant
EGAN00001402078	PP1339	Large NT>4.0	M	9	396881	A	G	DOCK8	missense_variant	Inherited	Compound heterozygous	Not relevant
EGAN00001402078	PP1339	Large NT>4.0	M	9	406999	C	T	DOCK8	missense_variant	Inherited	Compound heterozygous	Not relevant
EGAN00001366570	PP1342	Skeletal	M	16	70303579	C	T	AARS	missense_variant	Inherited	Homozygous	Not relevant
EGAN000013665	PP1357	Skeletal	M	5	174156248	G	A	MSX2	missense_variant	Inherited from	Heterozygous	Not relevant

91										affected parent		
EGAN000013665 94	PP1 360	Brain	M	9	98220390	G	A	PTCH1	missense_va riant	Uncertain (dyad)	Heterozygous	Not relevant
EGAN000013665 94	PP1 360	Brain	M	10	89720663	C	T	PTEN	missense_va riant	Uncertain (dyad)	Heterozygous	Not relevant
EGAN000013666 03	PP1 369	Large NT>4.0	F	1	11525874 8	C	T	NRAS	missense_va riant	de novo	Heterozygous	Potentially clinically useful
EGAN000013666 36	PP1 396	Brain	M	X	21899039	G	A	MBTPS2	missense_va riant	Inherited from unaffected mother (X)	Hemizygous	Potentially clinically useful
EGAN000014101 55	PP1 408	Multisyste m	M	17	70118955	C	T	SOX9	missense_va riant	de novo	Heterozygous	Diagnostic
EGAN000014281 72	PP1 417	Large NT>4.0	F	7	56082835	G	T	PSPH	missense_va riant	Inherited	Compound heterozygous	Not relevant
EGAN000014281 72	PP1 417	Large NT>4.0	F	7	56088791	C	T	PSPH	missense_va riant	Inherited	Compound heterozygous	Not relevant
EGAN000014024 27	PP1 420	Multisyste m	F	11	6648209	G	A	DCHS1	missense_va riant	Inherited	Compound heterozygous	Not relevant
EGAN000014024 27	PP1 420	Multisyste m	F	11	6651689	G	GC AG CG C	DCHS1	inframe_inse rtion	Inherited	Compound heterozygous	Not relevant
EGAN000014024 27	PP1 420	Multisyste m	F	11	6647509	A	T	DCHS1	missense_va riant	Inherited	Compound heterozygous	Not relevant
EGAN000014101 73	PP1 462	Multisyste m	M	7	14050130 2	T	C	BRAF	missense_va riant	de novo	Heterozygous	Diagnostic
EGAN000014024 30	PP1 471	Multisyste m	M	X	49092100	G	C	CCDC22	missense_va riant	Inherited from unaffected mother (X)	Hemizygous	Not relevant
EGAN000014024 30	PP1 471	Multisyste m	M	19	39071058	G	A	RYR1	missense_va riant	Inherited	Compound heterozygous	Not relevant

EGAN00001402430	PP1471	Multisystem	M	19	38991601	G	A	RYR1	missense_variant	Inherited	Compound heterozygous	Not relevant
EGAN00001402349	PP1474	Chest	F	7	100404994	C	T	EPHB4	missense_variant	de novo	Heterozygous	Not relevant
EGAN00001401997	PP1522	Multisystem	F	8	145738796	G	A	RECQL4	stop_gained	Inherited	Compound heterozygous	Potentially clinically useful
EGAN00001401997	PP1522	Multisystem	F	8	145740360	G	C	RECQL4	missense_variant	Inherited	Compound heterozygous	Potentially clinically useful
EGAN00001402000	PP1525	Abdominal	F	7	146818170	G	C	CNTNAP2	missense_variant	Inherited	Compound heterozygous	Not relevant
EGAN00001402000	PP1525	Abdominal	F	7	146536994	T	G	CNTNAP2	missense_variant	Inherited	Compound heterozygous	Not relevant
EGAN00001402003	PP1528	Large NT>4.0	M	12	112926910	G	T	PTPN11	missense_variant	de novo	Heterozygous	Potentially clinically useful
EGAN00001402018	PP1546	Large NT>4.0	F	19	39068824	G	A	RYR1	missense_variant	Inherited	Compound heterozygous	Not relevant
EGAN00001402018	PP1546	Large NT>4.0	F	19	38958251	TC	T	RYR1	frameshift_variant	Inherited	Compound heterozygous	Not relevant
EGAN00001402021	PP1549	Cardiac	M	21	46898244	G	A	COL18A1	missense_variant	Inherited	Compound heterozygous	Not relevant
EGAN00001402021	PP1549	Cardiac	M	21	46913431	C	T	COL18A1	missense_variant	Inherited	Compound heterozygous	Not relevant
EGAN00001402021	PP1549	Cardiac	M	3	78695341	C	G	ROBO1	splice_acceptor_variant	de novo	Heterozygous	Potentially clinically useful
EGAN00001402030	PP1561	Skeletal	F	3	178936091	G	A	PIK3CA	missense_variant	de novo	Heterozygous	Diagnostic
EGAN00001402042	PP1573	Hydrops	F	12	49443834	GC	G	KMT2D	frameshift_variant	de novo	Heterozygous	Diagnostic
EGAN00001402048	PP1579	Brain	M	6	30691699	C	T	TUBB	missense_variant	de novo	Heterozygous	Diagnostic

EGAN00001402054	PP1588	Skeletal	F	12	2721073	A	G	CACNA1C	missense_variant	de novo	Heterozygous	Not relevant
EGAN00001402066	PP1603	Spinal	F	11	68707139	T	G	IGHMBP2	missense_variant	Inherited	Compound heterozygous	Not relevant
EGAN00001402066	PP1603	Spinal	F	11	68701322	C	T	IGHMBP2	missense_variant	Inherited	Compound heterozygous	Not relevant
EGAN00001402069	PP1606	Spinal	M	X	66766138	C	T	AR	missense_variant	Inherited from unaffected mother (X)	Hemizygous	Not relevant
EGAN00001402442	PP1627	Multisystem	F	16	88804403	A	G	PIEZO1	missense_variant	Inherited	Compound heterozygous	Diagnostic
EGAN00001402442	PP1627	Multisystem	F	16	88798751	C	G	PIEZO1	missense_variant	Inherited	Compound heterozygous	Diagnostic
EGAN00001402442	PP1627	Multisystem	F	16	88786487	C	T	PIEZO1	missense_variant	Inherited	Compound heterozygous	Diagnostic
EGAN00001402146	PP1663	Multisystem	M	6	32037996	G	A	TNXB	missense_variant	Inherited	Compound heterozygous	Not relevant
EGAN00001402146	PP1663	Multisystem	M	6	32057125	C	T	TNXB	missense_variant	Inherited	Compound heterozygous	Not relevant
EGAN00001402164	PP1681	Multisystem	M	13	25484005	AAATT	A	CENPJ	frameshift_variant	Inherited	Compound heterozygous	Not relevant
EGAN00001402164	PP1681	Multisystem	M	13	25473696	T	C	CENPJ	missense_variant	Inherited	Compound heterozygous	Not relevant
EGAN00001402164	PP1681	Multisystem	M	13	25484011	GGAGAC	G	CENPJ	frameshift_variant	Inherited	Compound heterozygous	Not relevant
EGAN00001402352	PP1711	Facial/cleft lip±palate	F	1	149898588	GT	G	SF3B4	frameshift_variant	de novo	Heterozygous	Diagnostic
EGAN00001402361	PP1714	Chest	F	1	7724486	G	A	CAMTA1	missense_variant	de novo	Heterozygous	Not relevant
EGAN000014023	PP1720	Brain	F	8	61757601	C	T	CHD7	stop_gained	de novo	Heterozygous	Potentially clinically

67												useful
EGAN00 0014023 70	PP1 723	Large NT>4.0	M	11	11837314 1	AG	A	KMT2A	frameshift_v ariant	de novo	Heterozygous	Potentially clinically useful
EGAN00 0014023 73	PP1 726	Cardiac	M	6	14970045 3	GTC	G	TAB2	frameshift_v ariant	de novo	Heterozygous	Diagnostic
EGAN00 0014024 09	PP1 738	Multisyste m	F	16	30977381	G	A	SETD1A	missense_va riant	de novo	Heterozygous	Not relevant
EGAN00 0014100 41	PP1 750	Cardiac	M	16	89346991	ATC	A	ANKRD11	frameshift_v ariant	de novo	Heterozygous	Diagnostic
EGAN00 0014100 44	PP1 753	Multisyste m	F	11	2906501	G	GC C	CDKN1C	frameshift_v ariant	Inherited	Heterozygous	Diagnostic
EGAN00 0014100 47	PP1 756	Multisyste m	M	2	21585129 0	T	C	ABCA12	missense_va riant	Inherited	Homozygous	Not relevant
EGAN00 0014100 47	PP1 756	Multisyste m	M	X	15329624 9	G	A	MECP2	missense_va riant	Inherited from unaffected mother (X)	Hemizygous	Not relevant
EGAN00 0014100 65	PP1 774	Facial/clef t lip±palate	F	11	12105869 1	C	A	TECTA	stop_gained	Inherited	Compound heterozygous	Not relevant
EGAN00 0014100 65	PP1 774	Facial	F	11	12099874 7	C	G	TECTA	missense_va riant	Inherited	Compound heterozygous	Not relevant
EGAN00 0014100 68	PP1 777	Cardiac	M	X	70389118	C	T	NLGN3	missense_va riant	Inherited from unaffected mother (X)	Hemizygous	Not relevant
EGAN00 0014100 71	PP1 780	Multisyste m	M	11	824686	C	T	PNPLA2	missense_va riant	Inherited	Homozygous	Not relevant
EGAN00 0014100 71	PP1 780	Multisyste m	M	12	12418424 9	A	G	TCTN2	splice_accep tor_variant	Inherited	Homozygous	Diagnostic
EGAN00 0014100 77	PP1 786	Multisyste m	M	6	33146747	G	A	COL11A2	missense_va riant	Inherited	Compound heterozygous	Not relevant
EGAN00 0014100	PP1 786	Multisyste m	M	6	33146086	G	A	COL11A2	missense_va riant	Inherited	Compound heterozygous	Not relevant

77												
EGAN00 0014100 86	PP1 795	Multisyste m	M	16	57493495	C	T	COQ9	stop_gained	Inherited	Homozygous	Diagnostic
EGAN00 0014100 98	PP1 807	Large NT>4.0	M	X	10442702	G	A	MID1	stop_gained	de novo	Hemizygous	Diagnostic
EGAN00 0014101 01	PP1 810	Cardiac	F	2	74146668	C	T	ACTG2	missense_va riant	de novo	Heterozygous	Not relevant
EGAN00 0014101 04	PP1 813	Multisyste m	F	17	41256947	C	T	BRCA1	missense_va riant	Inherited	Homozygous	Not relevant
EGAN00 0014101 13	PP1 822	Brain	M	X	10993199 3	A	C	CHRDL1	missense_va riant	Inherited from unaffected mother (X)	Hemizygous	Not relevant
EGAN00 0014101 19	PP1 828	Abdomina l	F	6	12963592 0	G	A	LAMA2	missense_va riant	Inherited	Compound heterozygous	Not relevant
EGAN00 0014101 19	PP1 828	Abdomina l	F	6	12947013 6	G	A	LAMA2	missense_va riant	Inherited	Compound heterozygous	Not relevant
EGAN00 0014101 22	PP1 831	Brain	F	10	90707070	G	A	ACTA2	missense_va riant	Uncertain	Heterozygous	Not relevant
EGAN00 0014101 22	PP1 831	Brain	F	12	6691785	A	G	CHD4	missense_va riant	Uncertain	Heterozygous	Not relevant
EGAN00 0014101 22	PP1 831	Brain	F	9	27157912	G	T	TEK	missense_va riant	Uncertain	Heterozygous	Not relevant
EGAN00 0014101 37	PP1 843	Multisyste m	M	6	11644232 6	C	G	COL10A1	missense_va riant	de novo	Heterozygous	Not relevant
EGAN00 0014101 37	PP1 843	Multisyste m	M	12	49435258	G	A	KMT2D	stop_gained	de novo	Heterozygous	Diagnostic
EGAN00 0014101 46	PP1 852	Large NT>4.0	F	1	15137746 2	A	G	POGZ	missense_va riant	de novo	Heterozygous	Not relevant
EGAN00 0014101 49	PP1 855	Hydrops	M	X	99663003	C	T	PCDH19	missense_va riant	Inherited from unaffected	Hemizygous	Not relevant

										mother (X)		
EGAN00001410161	PP1864	Cardiac	M	12	49447760	C	T	KMT2D	splice_donor_variant	de novo	Heterozygous	Diagnostic
EGAN00001428337	PP1882	Brain	M	9	139400248	G	A	NOTCH1	missense_variant	de novo	Heterozygous	Not relevant
EGAN00001428001	PP1892	Cardiac	F	2	39278352	G	GCTT	SOS1	protein_altering_variant	de novo	Heterozygous	Diagnostic
EGAN00001428007	PP1898	Brain	F	11	124747839	G	T	ROBO3	missense_variant	Inherited	Compound heterozygous	Not relevant
EGAN00001428007	PP1898	Brain	F	11	124744752	C	T	ROBO3	missense_variant	Inherited	Compound heterozygous	Not relevant
EGAN00001428043	PP1934	Skeletal	M	17	48266844	C	A	COL1A1	missense_variant	de novo	Heterozygous	Diagnostic
EGAN00001428043	PP1934	Skeletal	M	19	39002913	G	A	RYR1	missense_variant	Inherited	Compound heterozygous	Not relevant
EGAN00001428043	PP1934	Skeletal	M	19	39055825	GCACGGCGGC	GCACGGCGGCCACGGCGGC	RYR1	inframe_insertion	Inherited	Compound heterozygous	Not relevant
EGAN00001428076	PP1967	Multisystem	M	16	88782438	C	T	PIEZO1	missense_variant	de novo	Heterozygous	Not relevant
EGAN00001428076	PP1967	Multisystem	M	12	112888202	C	T	PTPN11	missense_variant	de novo	Heterozygous	Diagnostic
EGAN00001428079	PP1970	Chest	M	X	149680610	G	T	MAMLD1	missense_variant	Inherited from unaffected mother (X)	Hemizygous	Not relevant
EGAN00001428097	PP1988	Skeletal	M	15	89876873	CCGTCGCTGG	C	POLG	inframe_deletion	Inherited	Compound heterozygous	Not relevant

						GGT						
EGAN000014280 97	PP1988	Skeletal	M	15	89864238	T	G	POLG	missense_variant	Inherited	Compound heterozygous	Not relevant
EGAN000014281 09	PP2000	Multisystem	M	19	38993358	C	A	RYR1	stop_gained	Inherited	Compound heterozygous	Diagnostic
EGAN000014281 09	PP2000	Multisystem	M	19	39010008	GCTGCTGGTGCGGGACGAGTTCT	G	RYR1	frameshift_variant	Inherited	Compound heterozygous	Diagnostic
EGAN000014281 12	PP2003	Hydrops	F	1	17312692	G	A	ATP13A2	missense_variant	Inherited	Compound heterozygous	Not relevant
EGAN000014281 12	PP2003	Hydrops	F	1	17312830	G	A	ATP13A2	missense_variant	Inherited	Compound heterozygous	Not relevant
EGAN000014281 18	PP2009	Skeletal	M	11	118452006	G	GT	ARCN1	frameshift_variant	de novo	Heterozygous	Diagnostic
EGAN000014281 24	PP2015	Multisystem	F	3	58120485	G	C	FLNB	missense_variant	de novo	Heterozygous	Diagnostic
EGAN000014281 39	PP2030	Cardiac	M	16	71015315	C	T	HYDIN	missense_variant	Inherited	Compound heterozygous	Not relevant
EGAN000014281 39	PP2030	Cardiac	M	16	70867931	C	T	HYDIN	missense_variant	Inherited	Compound heterozygous	Not relevant
EGAN000014281 39	PP2030	Cardiac	M	6	152652845	CCAA	C	SYNE1	inframe_deletion	Inherited	Compound heterozygous	Not relevant
EGAN000014281 39	PP2030	Cardiac	M	6	152590337	G	T	SYNE1	missense_variant	Inherited	Compound heterozygous	Not relevant
EGAN000014281 42	PP2033	Cardiac	M	8	61654644	A	AG	CHD7	frameshift_variant	de novo	Heterozygous	Diagnostic
EGAN000014281	PP2039	Hydrops	M	5	36976444	C	T	NIPBL	stop_gained	de novo	Heterozygous	Diagnostic

48												
EGAN00001428154	PP2045	Abdominal	F	1	235993614	T	C	LYST	missense_variant	Inherited	Compound heterozygous	Not relevant
EGAN00001428154	PP2045	Abdominal	F	1	235827838	C	T	LYST	missense_variant	Inherited	Compound heterozygous	Not relevant
EGAN00001428157	PP2048	Hydrops	M	X	153593586	C	T	FLNA	missense_variant	Inherited from unaffected mother (X)	Hemizygous	Not relevant
EGAN00001428160	PP2051	Renal	M	X	48933402	C	T	WDR45	splice_acceptor_variant	Inherited from unaffected mother (X)	Hemizygous	Not relevant
EGAN00001471937	PP2093	Large NT>4.0	F	2	220342126	C	T	SPEG	missense_variant	Inherited	Compound heterozygous	Not relevant
EGAN00001471937	PP2093	Large NT>4.0	F	2	220354255	G	T	SPEG	missense_variant	Inherited	Compound heterozygous	Not relevant
EGAN00001543690	PP2096	Hydrops	F	19	8467005	A	T	RAB11B	missense_variant	de novo	Heterozygous	Not relevant
EGAN00001543738	PP2141	Skeletal	M	4	1803564	C	T	FGFR3	missense_variant	de novo	Heterozygous	Diagnostic
EGAN00001471892	PP2378	Multisystem	F	2	227966249	G	A	COL4A4	missense_variant	Inherited	Compound heterozygous	Not relevant
EGAN00001471892	PP2378	Multisystem	F	2	227927312	C	T	COL4A4	missense_variant	Inherited	Compound heterozygous	Not relevant
EGAN00001471925	PP2396	Multisystem	F	3	71821955	G	A	PROK2	missense_variant	de novo	Heterozygous	Not relevant
EGAN00001471958	PP2399	Multisystem	F	5	176709563	A	G	NSD1	missense_variant	de novo	Heterozygous	Potentially clinically useful
EGAN00001471961	PP2402	Multisystem	M	X	149681311	G	A	MAMLD1	missense_variant	Inherited from unaffected mother (X)	Hemizygous	Not relevant

EGAN00001471907	PP2408	Brain	M	14	57114193	T	C	TMEM260	missense_variant	Inherited	Compound heterozygous	Not relevant
EGAN00001471907	PP2408	Brain	M	14	57072337	A	G	TMEM260	missense_variant	Inherited	Compound heterozygous	Not relevant
EGAN00001471910	PP2489	Multisystem	F	5	139493771	C	A	PURA	missense_variant	de novo	Heterozygous	Not relevant
EGAN00001472072	PP2492	Multisystem	M	1	19215893	T	C	ALDH4A1	missense_variant	Inherited	Homozygous	Not relevant
EGAN00001472072	PP2492	Multisystem	M	X	68060265	G	A	EFNB1	missense_variant	Inherited from unaffected mother (X)	Hemizygous	Not relevant
EGAN00001472072	PP2492	Multisystem	M	1	22191394	C	T	HSPG2	missense_variant	Inherited	Homozygous	Not relevant
EGAN00001471940	PP2501	Multisystem	M	16	56368732	G	A	GNAO1	missense_variant	de novo	Heterozygous	Not relevant
EGAN00001471979	PP2534	Hydrops	M	13	110861234	G	T	COL4A1	missense_variant	de novo	Heterozygous	Not relevant
EGAN00001471922	PP2543	Skeletal	M	2	8918823	C	G	KIDINS220	missense_variant	de novo	Heterozygous	Not relevant
EGAN00001472057	PP2564	Hydrops	M	4	140299961	C	G	NAA15	missense_variant	de novo	Heterozygous	Not relevant
EGAN00001472057	PP2564	Hydrops	M	X	122758006	G	A	THOC2	missense_variant	de novo	Hemizygous	Not relevant
EGAN00001472096	PP2609	Hydrops	M	2	216285498	A	T	FN1	missense_variant	de novo	Heterozygous	Not relevant
EGAN00001472066	PP2636	Skeletal	M	X	84520205	G	A	ZNF711	missense_variant	Inherited from unaffected mother (X)	Hemizygous	Not relevant
EGAN00001471952	PP2645	Multisystem	M	6	10402841	G	A	TFAP2A	missense_variant	de novo	Heterozygous	Diagnostic

EGAN000014718 67	PP2690	Brain	M	X	153296177	G	A	MECP2	missense_variant	Inherited from unaffected mother (X)	Hemizygous	Potentially clinically useful
EGAN000014718 85	PP2708	Chest	M	17	38240883	C	T	THRA	missense_variant	de novo	Heterozygous	Not relevant
EGAN000014719 91	PP2718	Multisystem	M	8	61654979	C	T	CHD7	stop_gained	de novo	Heterozygous	Diagnostic
EGAN000014719 97	PP2724	Skeletal	M	19	8668617	A	T	ADAMTS10	missense_variant	Inherited	Compound heterozygous	Not relevant
EGAN000014719 97	PP2724	Skeletal	M	19	8656993	T	TG	ADAMTS10	frameshift_variant	Inherited	Compound heterozygous	Not relevant
EGAN000014720 15	PP2742	Abdominal	F	X	53576285	G	A	HUWE1	missense_variant	de novo	Heterozygous	Not relevant
EGAN000014720 21	PP2748	Skeletal	M	16	9923339	C	G	GRIN2A	missense_variant	de novo	Heterozygous	Not relevant
EGAN000014720 24	PP2751	Multisystem	M	X	13773312	C	T	OFD1	missense_variant	Inherited from unaffected mother (X)	Hemizygous	Not relevant
EGAN000014721 26	PP2784	Brain	M	X	70614084	G	A	TAF1	missense_variant	Inherited from unaffected mother (X)	Hemizygous	Not relevant
EGAN000014720 69	PP2886	Cardiac	M	X	153595827	A	T	FLNA	missense_variant	Inherited from affected parent	Hemizygous	Not relevant
EGAN000014720 69	PP2886	Cardiac	M	3	71823660	T	C	PROK2	splice_acceptor_variant	Inherited from affected parent	Heterozygous	Potentially clinically useful
EGAN000014721 02	PP2895	Abdominal	M	6	152651557	G	A	SYNE1	missense_variant	Inherited	Compound heterozygous	Not relevant
EGAN000014721 02	PP2895	Abdominal	M	6	152804264	C	T	SYNE1	missense_variant	Inherited	Compound heterozygous	Not relevant
EGAN000014764	PP2904	Spinal	M	7	100419941	T	TG	EPHB4	frameshift_variant	de novo	Heterozygous	Diagnostic

53												
EGAN00001476498	PP2949	Cardiac	M	X	21887700	G	A	MBTPS2	missense_variant	Inherited from unaffected mother (X)	Hemizygous	Not relevant
EGAN00001476501	PP2952	Cardiac	M	X	99917257	T	C	SRPX2	missense_variant	Inherited from unaffected mother (X)	Hemizygous	Not relevant
EGAN00001476527	PP2979	Multisystem	M	8	61654746	CG	C	CHD7	frameshift_variant	de novo	Heterozygous	Diagnostic
EGAN00001589013	PP3045	Multisystem	M	2	219525942	A	G	BCS1L	missense_variant	Inherited	Compound heterozygous	Potentially clinically useful
EGAN00001589013	PP3045	Multisystem	M	2	219527701	A	G	BCS1L	missense_variant	Inherited	Compound heterozygous	Potentially clinically useful
EGAN00001589004	PP3111	Renal	M	19	46272041	G	GCCGCCA	SIX5	inframe_insertion	Uncertain (dyad)	Heterozygous	Not relevant
EGAN00001531002	PP3144	Abdominal	M	X	76872090	G	A	ATRX	missense_variant	Inherited from unaffected mother (X)	Hemizygous	Potentially clinically useful
EGAN00001589022	PP3168	Multisystem	F	1	155874266	A	G	RIT1	missense_variant	de novo	Heterozygous	Diagnostic
EGAN00001543676	PP3189	Facial/cleft lip±palate	M	6	30691720	T	C	TUBB	missense_variant	de novo	Heterozygous	Not relevant
EGAN00001588989	PP3204	Cardiac	F	18	6977821	G	A	LAMA1	missense_variant	Inherited	Compound heterozygous	Not relevant
EGAN00001588989	PP3204	Cardiac	F	18	6971976	G	T	LAMA1	missense_variant	Inherited	Compound heterozygous	Not relevant
EGAN00001588980	PP3213	Brain	M	6	152774686	AG	A	SYNE1	frameshift_variant	Inherited	Compound heterozygous	Not relevant
EGAN00001588980	PP3213	Brain	M	6	152697692	G	C	SYNE1	missense_variant	Inherited	Compound heterozygous	Not relevant

EGAN00001588980	PP3213	Brain	M	6	152631565	C	G	SYNE1	missense_variant	Inherited	Compound heterozygous	Not relevant
EGAN00001543685	PP3246	Skeletal	M	3	111298085	A	G	CD96	missense_variant	Uncertain (dyad)	Heterozygous	Not relevant
EGAN00001543685	PP3246	Skeletal	M	13	101844299	T	C	NALCN	missense_variant	de novo (presumed (dyad)	Heterozygous	Diagnostic
EGAN00001543729	PP3255	Multisystem	M	9	139404264	C	T	NOTCH1	missense_variant	Uncertain (dyad)	Heterozygous	Not relevant
EGAN00001543731	PP3258	Multisystem	F	11	64410183	T	TGGCGGC	NRXN2	inframe_insertion	Uncertain (dyad)	Heterozygous	Not relevant
EGAN00001598943	PP3276	Brain	F	1	43912636	T	C	SZT2	missense_variant	Inherited	Homozygous	Not relevant
EGAN00001598937	PP3285	Cardiac	F	7	5568962	G	C	ACTB	missense_variant	de novo	Heterozygous	Potentially clinically useful
EGAN00001530999	PP3297	Cardiac	M	X	39934079	C	T	BCOR	missense_variant	Inherited from unaffected mother (X)	Hemizygous	Not relevant
EGAN00001530999	PP3297	Cardiac	M	X	30326874	C	T	NR0B1	missense_variant	Inherited from unaffected mother (X)	Hemizygous	Not relevant
EGAN00001531035	PP3318	Chest	M	11	65978677	C	T	PACS1	missense_variant	de novo	Heterozygous	Potentially clinically useful
EGAN00001531050	PP3333	Skeletal	F	5	13737379	G	A	DNAH5	missense_variant	Inherited	Compound heterozygous	Not relevant
EGAN00001531050	PP3333	Skeletal	F	5	13794057	C	A	DNAH5	missense_variant	Inherited	Compound heterozygous	Not relevant
EGAN00001588983	PP3384	Multisystem	M	X	20206645	G	T	RPS6KA3	missense_variant	Inherited from unaffected mother (X)	Hemizygous	Not relevant
EGAN00001588974	PP3387	Multisystem	M	6	57059615	A	T	RAB23	stop_gained	Inherited	Homozygous	Diagnostic

EGAN00001598979	PP3411	Skeletal	F	17	48266554	C	A	COL1A1	missense_variant	de novo	Heterozygous	Not relevant
EGAN00001588977	PP3474	Brain	F	20	62045481	C	T	KCNQ2	missense_variant	de novo	Heterozygous	Potentially clinically useful
EGAN00001588965	PP3537	Brain	M	X	153135308	C	A	L1CAM	missense_variant	Inherited from unaffected mother (X)	Hemizygous	Not relevant
EGAN00001588959	PP3540	Skeletal	F	4	1806119	G	A	FGFR3	missense_variant	de novo	Heterozygous	Diagnostic
EGAN00001588962	PP3573	Multisystem	M	X	70510553	TCACCACCAGCAGCAG	T	NONO	inframe_deletion	Inherited from unaffected mother (X)	Hemizygous	Not relevant
EGAN00001598922	PP3597	Multisystem	F	1	22215194	G	A	HSPG2	missense_variant	Inherited	Compound heterozygous	Not relevant
EGAN00001598922	PP3597	Multisystem	F	1	22188289	G	A	HSPG2	missense_variant	Inherited	Compound heterozygous	Not relevant
EGAN00001598931	PP3603	Skeletal	F	X	53586423	G	A	HUWE1	missense_variant	de novo	Heterozygous	Not relevant
EGAN00001598931	PP3603	Skeletal	F	4	88996658	C	T	PKD2	missense_variant	de novo	Heterozygous	Not relevant
EGAN00001366473	PP0626	Multisystem	M	1	155874247	C	G	RIT1	missense_variant	Inherited	Heterozygous	Diagnostic
EGAN00001472087	PP2567	Hydrops	M	12	112915523	A	G	PTPN11	missense_variant	Inherited	Heterozygous	Diagnostic
EGAN00001367088	PP0503	Large NT>4.0	F	12	112915523	A	G	PTPN11	missense_variant	Inherited	Heterozygous	Diagnostic

Supplementary Table 5 – Copy number variants (CNVs) and uniparental disomies (UPDs) reviewed by Clinical Review Panel (hg19 coordinates)

EGA_ID	PP_ID	Phenotypic Class	Fetal sex	Chr	Start	End	Alt	Gene	Inheritance	Zygosity	Review Outcome
EGAN0000136 7161	PP0576	Skeletal	F	16	17564127	18604098	1.0Mb deletion	XYLT1	de novo	Heterozygous	Not relevant
EGAN0000147 1916	PP2444	Facial	M	X	6968281	8095281	1.1Mb duplication	STS	Inherited from unaffected mother (X)	Hemizygous	Not relevant
EGAN0000136 6819	PP0270	Large NT>4.0	M	17	34842342	36234001	1.4Mb deletion	YWHAEP7|ZNHIT3|AATF|MYO19|DUSP14|RNA5SP439|DHRS11|HNF1B|MIR2909|TADA2A|SYNRG|MRM1|GGNBP2|PIGW|C17orf78|DDX52|HMGB1P24|ACACA|LHX1	de novo	Heterozygous	Unknown relevance
EGAN0000136 6413	PP0087	Skeletal	F	12	1192189	2695179	1.5Mb deletion	ERC1|CACNA1C-IT1|CACNA1C-IT3|RPS4XP14|CACNA1C-AS4|LINC00942|CACNA1C|LRTM2|LINC00940|ADIPOR2|MIR3649|WNT5B|CACNA1C-IT2|FBXL14|CACNA2D4|DCP1B	de novo	Heterozygous	Not relevant
EGAN0000154 3670	PP3033	Facial	M	22	41564321	41575064	10.7kb deletion	EP300	de novo	Heterozygous	Not relevant

EGAN0000 1366864	PP0315	Multisystem	F	1	103352183	103484596	132.4kb duplication	COL11A1	Inherited	Compound heterozygous (with SNV)	Not relevant
EGAN0000 1428160	PP2051	Renal	M	16	86429966	86613477	183kb deletion	FOXF1|FOXC2	de novo	Heterozygous	Potentially clinically useful
EGAN0000 1366740	PP0225	Multisystem	M	22	18893860	21414945	2.5Mb duplication	DGCR9|FAM230A|PPP1R26P3|RNU6-225P|DGCR2|SMPD4P1|POM121L4P|ABHD17AP4|SLC9A3P2|GSC2|DGCR6L|TSSK2|DGCR10|THAP7|GP1BB|LZTR1|DGCR11|PI4KAP1|TRMT2A|SLC25A1|AIFM3|TMEM191A|PI4KA|RANBP1|MIR649|TSSK1A|MIR4761|CDC45|.|RNY1P9|CRKL|ARVCF|MIR185|TANGO2|TBX1|DGCR5|SEPT5|HIRA|C22orf39|GGTLC3|GNB1L|RN7SKP131|RN7SL168P|RTN4R|KRT18P62|USP41|TUBA3GP|UFD1L|COMT|CA15P2|CA15P1|RN7SL812P|C22orf29|MIR3618|DGCR6|DGCR14|PPP1R26P2|P2RX6P|MIR1286|KLHL22|MRPL40|SNAP29|MED15|LINC00896|BCRP5|ZDHHC8|SLC7A4|PROD	de novo	Heterozygous	Unknown relevance

								H|THAP7-AS1|DGCR8|CLTCL1|ZNF74|MIR1306|KRT18P5|SCARF2|TXNRD2|TUBA3FP|RIMBP3|CLDN5|SERPIND1|P2RX6			
EGAN0000154 3696	PP3042	Multisystem	M	X	30671604	30877908	206.3kb duplication	GK	Inherited from unaffected mother (X)	Hemizygous	Not relevant
EGAN0000136 6463	PP0713	Multisystem	F	9	1.38E+08	To be removed	25.2kb duplication	COL5A1	de novo	Heterozygous	Not relevant
EGAN0000136 7084	PP0500	Multisystem	M	22	17060170	20722040	3.7Mb deletion	TBX1|TUBA8|PEX26	de novo	Heterozygous	Unknown relevance
EGAN0000136 6772	PP0206	Large NT>4.0	M	X	135115409	135115784	375bp deletion	SLC9A6	de novo	Hemizygous	Potentially clinically useful
EGAN0000136 6512	PP0656	Multisystem	M	16	2120332	2161578	41.2kb deletion	PKD1	de novo	Heterozygous	Diagnostic
EGAN0000147 2126	PP2784	Brain	M	X	38505397	38547108	41.7kb duplication	TSPAN7	Inherited from unaffected mother (X)	Hemizygous	Not relevant
EGAN0000136 6808	PP0261	Large NT>4.0	M	1	154247351	154310314	63.2kb duplication	RNU6-121P|RNU6-239P|UBAP2L|RNU7-57P|ATP8B2|AQP10|HAX1	Inherited	Compound heterozygous (with SNV)	Not relevant

EGAN0000136 7125	PP0540	Multisystem	F	9	139964223	140695623	731.4kb duplication	TPRN|EHMT1	Inherited	Heterozygous	Not relevant
EGAN0000147 1901	PP2387	Abdominal	M	2	60687454	61484556	797.1kb duplication	BCL11A	de novo	Heterozygous	Not relevant
EGAN0000136 6751	PP0231	Cardiac	M	X	76854794	76940204	85.4kb duplication	ATRX	Inherited from unaffected mother (X)	Hemizygous	Not relevant
EGAN0000142 8160	PP2051	Renal	M	9	137732924	137742724	9.8kb duplication	COL5A1	Uncertain	Heterozygous	Not relevant
EGAN0000136 6422	PP0602	Large NT>4.0	M	15	UPD	NA	NA	UPD_chr15	Uniparental disomy	NA	Diagnostic
EGAN0000136 6714	PP0165	Skeletal	M	10	UPD	NA	NA	UPD_chr10	Uniparental disomy	NA	Not relevant

Note: All cases considered for inclusion in PAGE had QF-PCR carried to detect common aneuploidies (13,18,21, X and Y) and CMA carried out if QF-PCR was negative, concurrent with recruitment to PAGE. When a CNV was found which was classified as pathogenic and to likely to be the cause of FSA, WES was not initiated. However if clinical CMA failed or a CNV was detected but not considered unequivocally causative of the FSA then WES was performed.

Supplementary Table 6: Fetal outcomes and phenotypic classes; (NND = neonatal death)

EGAID	PPID	Pheno	Sex	Outcome
EGAN00001367173	PP0588	Abdominal	F	Live birth
EGAN00001366780	PP0210	Abdominal	F	Miscarriage
EGAN00001367079	PP0497	Abdominal	M	Termination
EGAN00001402027	PP1558	Abdominal	F	Termination
EGAN00001472102	PP2895	Abdominal	M	Termination
EGAN00001366699	PP0150	Abdominal	M	Unknown
EGAN00001366770	PP0240	Abdominal	M	Unknown
EGAN00001366781	PP0246	Abdominal	F	Unknown
EGAN00001366802	PP0258	Abdominal	F	Unknown
EGAN00001367039	PP0387	Abdominal	M	Unknown
EGAN00001366955	PP0437	Abdominal	M	Unknown
EGAN00001366989	PP0452	Abdominal	F	Unknown
EGAN00001367073	PP0494	Abdominal	F	Unknown
EGAN00001366530	PP0674	Abdominal	F	Unknown
EGAN00001402388	PP0969	Abdominal	F	Unknown
EGAN00001402400	PP0996	Abdominal	M	Unknown
EGAN00001402403	PP0999	Abdominal	F	Unknown
EGAN00001366555	PP1327	Abdominal	M	Unknown
EGAN00001366561	PP1333	Abdominal	F	Unknown
EGAN00001402000	PP1525	Abdominal	F	Unknown
EGAN00001402006	PP1531	Abdominal	F	Unknown
EGAN00001402101	PP1618	Abdominal	F	Unknown
EGAN00001402107	PP1624	Abdominal	M	Unknown
EGAN00001402158	PP1675	Abdominal	M	Unknown
EGAN00001402167	PP1684	Abdominal	M	Unknown
EGAN00001402176	PP1693	Abdominal	M	Unknown
EGAN00001410119	PP1828	Abdominal	F	Unknown
EGAN00001428040	PP1931	Abdominal	M	Unknown
EGAN00001428055	PP1946	Abdominal	F	Unknown
EGAN00001428154	PP2045	Abdominal	F	Unknown
EGAN00001471901	PP2387	Abdominal	M	Unknown
EGAN00001471928	PP2453	Abdominal	F	Unknown
EGAN00001471964	PP2519	Abdominal	M	Unknown
EGAN00001472075	PP2594	Abdominal	F	Unknown
EGAN00001471955	PP2648	Abdominal	F	Unknown
EGAN00001472120	PP2669	Abdominal	F	Unknown
EGAN00001472015	PP2742	Abdominal	F	Unknown
EGAN00001472030	PP2757	Abdominal	F	Unknown
EGAN00001543682	PP2841	Abdominal	M	Unknown
EGAN00001476465	PP2916	Abdominal	F	Unknown
EGAN00001476474	PP2925	Abdominal	M	Unknown
EGAN00001543699	PP3030	Abdominal	F	Unknown
EGAN00001543712	PP3105	Abdominal	M	Unknown
EGAN00001531002	PP3144	Abdominal	M	Unknown
EGAN00001530985	PP3291	Abdominal	M	Unknown
EGAN00001366730	PP0181	Brain	M	Termination
EGAN00001366736	PP0187	Brain	M	Termination
EGAN00001366785	PP0249	Brain	F	Termination
EGAN00001367015	PP0375	Brain	F	Termination

EGAN00001367021	PP0378	Brain	F	Termination
EGAN00001367033	PP0384	Brain	F	Termination
EGAN00001367007	PP0461	Brain	M	Termination
EGAN00001367044	PP0479	Brain	F	Termination
EGAN00001367134	PP0549	Brain	F	Termination
EGAN00001367146	PP0561	Brain	M	Termination
EGAN00001366448	PP0614	Brain	M	Termination
EGAN00001366497	PP0641	Brain	F	Termination
EGAN00001366542	PP0686	Brain	M	Termination
EGAN00001366429	PP0695	Brain	M	Termination
EGAN00001366476	PP0719	Brain	F	Termination
EGAN00001366549	PP0801	Brain	F	Termination
EGAN00001402355	PP0849	Brain	M	Termination
EGAN00001402397	PP0978	Brain	M	Termination
EGAN00001366618	PP1384	Brain	M	Termination
EGAN00001366621	PP1387	Brain	M	Termination
EGAN00001366636	PP1396	Brain	M	Termination
EGAN00001402048	PP1579	Brain	M	Termination
EGAN00001402098	PP1615	Brain	F	Termination
EGAN00001402367	PP1720	Brain	F	Termination
EGAN00001402382	PP1735	Brain	M	Termination
EGAN00001410050	PP1759	Brain	M	Termination
EGAN00001428337	PP1882	Brain	F	Termination
EGAN00001410107	PP1816	Brain	M	Termination
EGAN00001410170	PP1873	Brain	M	Termination
EGAN00001428007	PP1898	Brain	F	Termination
EGAN00001428025	PP1916	Brain	M	Termination
EGAN00001428046	PP1937	Brain	F	Termination
EGAN00001428049	PP1940	Brain	M	Termination
EGAN00001428073	PP1964	Brain	M	Termination
EGAN00001428130	PP2021	Brain	F	Termination
EGAN00001471907	PP2408	Brain	M	Termination
EGAN00001472090	PP2465	Brain	F	Termination
EGAN00001471967	PP2522	Brain	F	Termination
EGAN00001471970	PP2525	Brain	M	Termination
EGAN00001471867	PP2690	Brain	M	Termination
EGAN00001472000	PP2727	Brain	F	Termination
EGAN00001472012	PP2739	Brain	F	Termination
EGAN00001472126	PP2784	Brain	M	Termination
EGAN00001472081	PP2838	Brain	F	Termination
EGAN00001472084	PP2889	Brain	F	Termination
EGAN00001472108	PP2901	Brain	M	Termination
EGAN00001476456	PP2907	Brain	M	Termination
EGAN00001476459	PP2910	Brain	M	Termination
EGAN00001476480	PP2931	Brain	F	Termination
EGAN00001476492	PP2943	Brain	M	Termination
EGAN00001543667	PP3024	Brain	M	Termination
EGAN00001366491	PP0635	Brain	M	Unknown
EGAN00001366594	PP1360	Brain	M	Unknown
EGAN00001410122	PP1831	Brain	F	Unknown

EGAN00001476773	PP2976	brain	M	Unknown
EGAN00001588980	PP3213	Brain	M	Unknown
EGAN00001589010	PP3216	Brain	M	Unknown
EGAN00001598961	PP3267	Brain	M	Unknown
EGAN00001598943	PP3276	Brain	F	Unknown
EGAN00001598940	PP3288	Brain	F	Unknown
EGAN00001530984	PP3294	Brain	F	Unknown
EGAN00001531053	PP3336	Brain	F	Unknown
EGAN00001588977	PP3474	Brain	F	Unknown
EGAN00001588956	PP3504	Brain	F	Unknown
EGAN00001588965	PP3537	Brain	M	Unknown
EGAN00001589001	PP3561	Brain	F	Unknown
EGAN00001598919	PP3582	Brain	M	Unknown
EGAN00001598955	PP3618	Brain	F	Unknown
EGAN00001410113	PP1822	Brain	M	Unknown
EGAN00001366643	PP0093	Cardiac	M	Termination
EGAN00001366658	PP0108	Cardiac	F	Termination
EGAN00001366682	PP0132	Cardiac	M	Termination
EGAN00001366687	PP0138	Cardiac	F	Termination
EGAN00001366717	PP0168	Cardiac	M	Termination
EGAN00001366726	PP0177	Cardiac	M	Termination
EGAN00001366733	PP0184	Cardiac	F	Termination
EGAN00001366751	PP0231	Cardiac	M	Termination
EGAN00001366852	PP0303	Cardiac	M	Termination
EGAN00001366892	PP0330	Cardiac	M	Termination
EGAN00001366946	PP0339	Cardiac	F	Termination
EGAN00001366959	PP0345	Cardiac	M	Termination
EGAN00001367043	PP0390	Cardiac	M	Termination
EGAN00001366931	PP0425	Cardiac	F	Termination
EGAN00001366484	PP0434	Cardiac	M	Termination
EGAN00001367068	PP0491	Cardiac	M	Termination
EGAN00001367098	PP0513	Cardiac	F	Termination
EGAN00001367101	PP0516	Cardiac	M	Termination
EGAN00001367113	PP0528	Cardiac	M	Termination
EGAN00001367143	PP0558	Cardiac	M	Termination
EGAN00001367176	PP0591	Cardiac	F	Termination
EGAN00001366445	PP0704	Cardiac	F	Termination
EGAN00001366470	PP0716	Cardiac	M	Termination
EGAN00001366582	PP0729	Cardiac	M	Termination
EGAN00001366630	PP1320	Cardiac	M	Termination
EGAN00001366633	PP1323	Cardiac	M	Termination
EGAN00001366558	PP1330	Cardiac	F	Termination
EGAN00001366600	PP1366	Cardiac	M	Termination
EGAN00001402021	PP1549	Cardiac	M	Termination
EGAN00001402033	PP1564	Cardiac	M	Termination
EGAN00001402039	PP1570	Cardiac	M	Termination
EGAN00001402113	PP1630	Cardiac	F	Termination
EGAN00001402149	PP1666	Cardiac	M	Termination
EGAN00001402182	PP1699	Cardiac	M	Termination
EGAN00001402364	PP1717	Cardiac	F	Termination

EGAN00001402379	PP1732	Cardiac	M	Termination
EGAN00001402412	PP1741	Cardiac	F	Termination
EGAN00001410041	PP1750	Cardiac	M	Termination
EGAN00001410068	PP1777	Cardiac	M	Termination
EGAN00001410089	PP1798	Cardiac	M	Termination
EGAN00001410101	PP1810	Cardiac	F	Termination
EGAN00001410161	PP1864	Cardiac	M	Termination
EGAN00001410164	PP1867	Cardiac	M	Termination
EGAN00001428001	PP1892	Cardiac	F	Termination
EGAN00001428082	PP1973	Cardiac	M	Termination
EGAN00001428121	PP2012	Cardiac	M	Termination
EGAN00001428139	PP2030	Cardiac	M	Termination
EGAN00001428142	PP2033	Cardiac	M	Termination
EGAN00001471976	PP2531	Cardiac	M	Termination
EGAN00001472123	PP2672	Cardiac	M	Termination
EGAN00001471876	PP2699	Cardiac	M	Termination
EGAN00001471879	PP2702	Cardiac	M	Termination
EGAN00001471990	PP2717	Cardiac	F	Termination
EGAN00001472003	PP2730	Cardiac	M	Termination
EGAN00001472006	PP2733	Cardiac	M	Termination
EGAN00001472036	PP2763	Cardiac	M	Termination
EGAN00001472045	PP2769	Cardiac	F	Termination
EGAN00001472069	PP2886	Cardiac	M	Termination
EGAN00001476486	PP2937	Cardiac	M	Termination
EGAN00001476498	PP2949	Cardiac	M	Termination
EGAN00001476501	PP2952	Cardiac	M	Termination
EGAN00001476507	PP2958	Cardiac	M	Termination
EGAN00001476519	PP2970	Cardiac	F	Termination
EGAN00001476536	PP2988	Cardiac	F	Termination
EGAN00001543693	PP3027	Cardiac	M	Termination
EGAN00001543715	PP3072	Cardiac	F	Termination
EGAN00001543718	PP3096	Cardiac	F	Termination
EGAN00001543673	PP3114	Cardiac	F	Termination
EGAN00001530994	PP3138	Cardiac	F	Termination
EGAN00001589030	PP3141	Cardiac	F	Termination
EGAN00001543723	PP3159	Cardiac	M	Termination
EGAN00001588995	PP3171	Cardiac	M	Termination
EGAN00001543679	PP3192	Cardiac	F	Termination
EGAN00001588989	PP3204	Cardiac	F	Termination
EGAN00001588992	PP3222	Cardiac	F	Termination
EGAN00001598937	PP3285	Cardiac	F	Termination
EGAN00001530999	PP3297	Cardiac	M	Termination
EGAN00001588986	PP3393	Cardiac	M	Termination
EGAN00001598949	PP3612	Cardiac	M	Termination
EGAN00001366934	PP0333	Cardiac	M	Unknown
EGAN00001402373	PP1726	Cardiac	M	Live birth
EGAN00001366746	PP0228	Chest	M	Stillbirth
EGAN00001366828	PP0279	Chest	M	Stillbirth
EGAN00001366996	PP0366	Chest	M	Stillbirth
EGAN00001366872	PP0413	Chest	F	Stillbirth

EGAN00001367036	PP0476	Chest	F	Stillbirth
EGAN00001402358	PP1441	Chest	M	Stillbirth
EGAN00001402349	PP1474	Chest	F	Stillbirth
EGAN00001402060	PP1597	Chest	F	Stillbirth
EGAN00001402361	PP1714	Chest	F	Stillbirth
EGAN00001410110	PP1819	Chest	F	Stillbirth
EGAN00001428079	PP1970	Chest	M	Stillbirth
EGAN00001428100	PP1991	Chest	M	Stillbirth
EGAN00001428106	PP1997	Chest	M	Stillbirth
EGAN00001428133	PP2024	Chest	M	Stillbirth
EGAN00001471913	PP2441	Chest	F	Stillbirth
EGAN00001471934	PP2459	Chest	M	Termination
EGAN00001471885	PP2708	Chest	M	Termination
EGAN00001471994	PP2721	Chest	M	Termination
EGAN00001588998	PP2844	Chest	M	Termination
EGAN00001531014	PP3075	Chest	M	Termination
EGAN00001530981	PP3147	Chest	M	Termination
EGAN00001531008	PP3150	Chest	F	Termination
EGAN00001531035	PP3318	Chest	M	Termination
EGAN00001366655	PP0105	Facial	M	Miscarriage
EGAN00001366661	PP0111	Facial	M	Miscarriage
EGAN00001366732	PP0183	Facial	M	Miscarriage
EGAN00001366837	PP0288	Facial	M	Miscarriage
EGAN00001366843	PP0294	Facial	M	Miscarriage
EGAN00001366849	PP0300	Facial	M	Miscarriage
EGAN00001366940	PP0336	Facial	F	Miscarriage
EGAN00001366488	PP0464	Facial	M	Miscarriage
EGAN00001367018	PP0467	Facial	M	Miscarriage
EGAN00001367128	PP0543	Facial	M	NND
EGAN00001367167	PP0582	Facial	F	NND
EGAN00001410134	PP1263	Facial	M	NND
EGAN00001402051	PP1582	Facial	F	NND
EGAN00001402057	PP1591	Facial	F	NND
EGAN00001402352	PP1711	Facial	F	NND
EGAN00001410065	PP1774	Facial	F	NND
EGAN00001410080	PP1789	Facial	M	NND
EGAN00001428103	PP1994	Facial	M	NND
EGAN00001428127	PP2018	Facial	M	NND
EGAN00001428163	PP2054	Facial	M	NND
EGAN00001472039	PP2189	Facial	F	NND
EGAN00001471916	PP2444	Facial	M	NND
EGAN00001471931	PP2456	Facial	M	NND
EGAN00001471946	PP2507	Facial	M	Stillbirth
EGAN00001471895	PP2540	Facial	M	Stillbirth
EGAN00001472105	PP2898	Facial	F	Stillbirth
EGAN00001476516	PP2967	Facial	F	Stillbirth
EGAN00001543662	PP3015	Facial	M	Stillbirth
EGAN00001543670	PP3033	Facial	M	Stillbirth
EGAN00001543676	PP3189	Facial	M	Stillbirth
EGAN00001367062	PP0399	Facial	M	Termination

EGAN00001543665	PP3243	Facial	M	Unknown
EGAN00001366708	PP0159	Hydrops	F	Live birth
EGAN00001366711	PP0162	Hydrops	F	Live birth
EGAN00001366731	PP0182	Hydrops	F	Live birth
EGAN00001366735	PP0186	Hydrops	M	Live birth
EGAN00001366763	PP0237	Hydrops	M	Live birth
EGAN00001366878	PP0416	Hydrops	F	Live birth
EGAN00001366889	PP0422	Hydrops	M	Live birth
EGAN00001367104	PP0519	Hydrops	M	Live birth
EGAN00001367110	PP0525	Hydrops	F	Live birth
EGAN00001366506	PP0650	Hydrops	M	Live birth
EGAN00001366539	PP0683	Hydrops	M	Live birth
EGAN00001402391	PP0972	Hydrops	F	Live birth
EGAN00001402042	PP1573	Hydrops	F	Live birth
EGAN00001402075	PP1612	Hydrops	M	Live birth
EGAN00001410149	PP1855	Hydrops	M	Live birth
EGAN00001428112	PP2003	Hydrops	F	Live birth
EGAN00001428148	PP2039	Hydrops	M	Live birth
EGAN00001428157	PP2048	Hydrops	M	Live birth
EGAN00001543690	PP2096	Hydrops	F	Live birth
EGAN00001471943	PP2504	Hydrops	F	Live birth
EGAN00001472093	PP2513	Hydrops	F	Live birth
EGAN00001471979	PP2534	Hydrops	M	Live birth
EGAN00001472057	PP2564	Hydrops	M	Live birth
EGAN00001472087	PP2567	Hydrops	M	Livebirth
EGAN00001472078	PP2600	Hydrops	F	Livebirth
EGAN00001476483	PP2934	Hydrops	F	Miscarriage
EGAN00001588950	PP3039	Hydrops	M	Miscarriage
EGAN00001588953	PP3660	Hydrops	F	Miscarriage
EGAN00001472096	PP2609	Hydrops	M	Unknown
EGAN00001472135	PP2678	Hydrops	F	Unknown
EGAN00001472138	PP2681	Hydrops	M	Unknown
EGAN00001366705	PP0156	Hydrops	M	Unknown
EGAN00001531017	PP3078	Hydrops	F	Unknown
EGAN00001366673	PP0123	Large NT (5.3mm)	F	Live birth
EGAN00001366685	PP0135	Large NT (4mm)	M	Live birth
EGAN00001366690	PP0141	Large NT (7.1mm)	F	Live birth
EGAN00001366693	PP0144	Large NT (5.8mm)	F	Live birth
EGAN00001366696	PP0147	Large NT (5.8mm)	F	Live birth
EGAN00001366729	PP0180	Large NT (5.5mm)	M	Live birth
EGAN00001366734	PP0185	Large NT (4.4mm)	F	Live birth
EGAN00001366769	PP0205	Large NT (5.8mm)	F	Live birth
EGAN00001366772	PP0206	Large NT (11.3mm)	M	Live birth
EGAN00001366778	PP0209	Large NT (4.5mm)	M	Live birth
EGAN00001366757	PP0234	Large NT (4.2mm)	F	Live birth
EGAN00001366775	PP0243	Large NT (5.6mm)	M	Live birth
EGAN00001366808	PP0261	Large NT (5.2mm)	M	Live birth
EGAN00001366813	PP0264	Large NT (6.7mm)	F	Live birth
EGAN00001366819	PP0270	Large NT (4.9mm)	M	Live birth
EGAN00001366822	PP0273	Large NT (6.5mm)	F	Live birth

EGAN00001366825	PP0276	Large NT (4mm)	M	Live birth
EGAN00001366831	PP0282	Large NT (8.2mm)	F	Live birth
EGAN00001366834	PP0285	Large NT (6.5mm)	M	Live birth
EGAN00001366840	PP0291	Large NT (13.7mm)	M	Live birth
EGAN00001366846	PP0297	Large NT (4.1mm)	M	Live birth
EGAN00001366855	PP0306	Large NT (8.8mm)	F	Live birth
EGAN00001366861	PP0312	Large NT (4.8mm)	F	Live birth
EGAN00001366964	PP0348	Large NT (5.1mm)	F	Live birth
EGAN00001366990	PP0363	Large NT (6mm)	M	Live birth
EGAN00001367008	PP0372	Large NT (4.5mm)	F	Live birth
EGAN00001367050	PP0393	Large NT (7.6mm)	M	Live birth
EGAN00001367056	PP0396	Large NT (4.9mm)	M	Live birth
EGAN00001367067	PP0402	Large NT (4.3mm)	M	Live birth
EGAN00001367074	PP0405	Large NT (5.6mm)	F	Live birth
EGAN00001367080	PP0408	Large NT (11.5mm)	M	Live birth
EGAN00001366968	PP0443	Large NT (5.1mm)	M	Live birth
EGAN00001366995	PP0455	Large NT (4.4mm)	F	Live birth
EGAN00001367024	PP0470	Large NT (4.3mm)	F	Live birth
EGAN00001367088	PP0503	Large NT (4.5mm)	F	Live birth
EGAN00001367107	PP0522	Large NT (4.5mm)	F	Live birth
EGAN00001367116	PP0531	Large NT (4.4mm)	F	Live birth
EGAN00001367119	PP0534	Large NT (6.1mm)	M	Live birth
EGAN00001367152	PP0567	Large NT (7mm)	F	Live birth
EGAN00001367164	PP0579	Large NT (7.2mm)	M	Live birth
EGAN00001367170	PP0585	Large NT (4.6mm)	F	Live birth
EGAN00001366418	PP0599	Large NT (4.4mm)	M	Live birth
EGAN00001366422	PP0602	Large NT (4.78mm)	M	Live birth
EGAN00001366428	PP0605	Large NT (4mm)	M	Live birth
EGAN00001366435	PP0608	Large NT (5.3mm)	F	Live birth
EGAN00001366441	PP0611	Large NT (6.2mm)	F	Live birth
EGAN00001366460	PP0620	Large NT (4.2mm)	M	Live birth
EGAN00001366485	PP0632	Large NT (4.5mm)	M	Live birth
EGAN00001366494	PP0638	Large NT (6mm)	F	Live birth
EGAN00001366518	PP0662	Large NT (5mm)	F	Live birth
EGAN00001366521	PP0665	Large NT (4.7mm)	M	Live birth
EGAN00001366524	PP0668	Large NT (4.5mm)	M	Live birth
EGAN00001366545	PP0689	Large NT (4.5mm)	M	Live birth
EGAN00001366452	PP0707	Large NT (5mm)	M	Live birth
EGAN00001366482	PP0722	Large NT (6.7mm)	F	Live birth
EGAN00001402385	PP0966	Large NT (4mm)	F	Live birth
EGAN00001402433	PP1038	Large NT (4.4mm)	M	Live birth
EGAN00001402415	PP1047	Large NT (5.1mm)	M	Live birth
EGAN00001366564	PP1336	Large NT (4.4mm)	M	Live birth
EGAN00001402078	PP1339	Large NT (4.4mm)	M	Live birth
EGAN00001366573	PP1345	Large NT (4.1mm)	F	Live birth
EGAN00001366588	PP1354	Large NT (5.1mm)	F	Live birth
EGAN00001366597	PP1363	Large NT (5.1mm)	M	Live birth
EGAN00001366603	PP1369	Large NT (8.7mm)	F	Live birth
EGAN00001366606	PP1372	Large NT (4.3mm)	M	Live birth
EGAN00001366612	PP1378	Large NT (5mm)	F	Live birth

EGAN00001366615	PP1381	Large NT (4.2mm)	F	Live birth
EGAN00001402406	PP1414	Large NT (6.5mm)	M	Live birth
EGAN00001428172	PP1417	Large NT (4mm)	F	Live birth
EGAN00001402003	PP1528	Large NT (9.3mm)	M	Live birth
EGAN00001402018	PP1546	Large NT (10mm)	F	Live birth
EGAN00001402440	PP1585	Large NT (4mm)	F	Live birth
EGAN00001402441	PP1594	Large NT (4.1mm)	F	Live birth
EGAN00001402063	PP1600	Large NT (5mm)	M	Live birth
EGAN00001402072	PP1609	Large NT (4mm)	F	Live birth
EGAN00001410132	PP1633	Large NT (4.4mm)	F	Live birth
EGAN00001402128	PP1645	Large NT (4.4mm)	M	Live birth
EGAN00001402143	PP1660	Large NT (4.04mm)	M	Live birth
EGAN00001402370	PP1723	Large NT (5mm)	M	Live birth
EGAN00001402376	PP1729	Large NT (4.1mm)	M	Live birth
EGAN00001476542	PP1762	Large NT (5.4mm)	F	Live birth
EGAN00001410098	PP1807	Large NT (4.7mm)	M	Live birth
EGAN00001410140	PP1846	Large NT (4.2mm)	M	Live birth
EGAN00001410143	PP1849	Large NT (4.4mm)	F	Live birth
EGAN00001410146	PP1852	Large NT (6mm)	F	Live birth
EGAN00001410152	PP1858	Large NT (4mm)	M	Live birth
EGAN00001428088	PP1979	Large NT (5.3mm)	F	Live birth
EGAN00001428091	PP1982	Large NT (4.8mm)	M	Live birth
EGAN00001428136	PP2027	Large NT (4.1mm)	M	Live birth
EGAN00001428166	PP2057	Large NT (4.5mm)	M	Live birth
EGAN00001471937	PP2093	Large NT (4.4mm)	F	Live birth
EGAN00001471898	PP2477	Large NT (4.2mm)	F	Live birth
EGAN00001471982	PP2537	Large NT (4.6mm)	M	Live birth
EGAN00001402430	PP1471	Multisystem	M	Live birth
EGAN00001410137	PP1843	Multisystem	M	Live birth
EGAN00001428109	PP2000	Multisystem	M	Live birth
EGAN00001366640	PP0090	Multisystem	M	Live birth
EGAN00001366646	PP0096	Multisystem	F	Live birth
EGAN00001366676	PP0126	Multisystem	M	Live birth
EGAN00001366720	PP0171	Multisystem	F	Live birth
EGAN00001366723	PP0174	Multisystem	F	Live birth
EGAN00001366740	PP0225	Multisystem	M	Live birth
EGAN00001366797	PP0255	Multisystem	M	Live birth
EGAN00001366864	PP0315	Multisystem	F	Live birth
EGAN00001366880	PP0324	Multisystem	F	Live birth
EGAN00001366886	PP0327	Multisystem	M	Live birth
EGAN00001366953	PP0342	Multisystem	F	Live birth
EGAN00001366960	PP0440	Multisystem	F	Live birth
EGAN00001366975	PP0446	Multisystem	F	Live birth
EGAN00001367030	PP0473	Multisystem	M	Live birth
EGAN00001367048	PP0482	Multisystem	M	Live birth
EGAN00001367084	PP0500	Multisystem	M	Live birth
EGAN00001367125	PP0540	Multisystem	F	Live birth
EGAN00001367137	PP0552	Multisystem	F	Live birth
EGAN00001367140	PP0555	Multisystem	M	Live birth
EGAN00001367155	PP0570	Multisystem	F	Live birth

EGAN00001367158	PP0573	Multisystem	F	Live birth
EGAN00001366454	PP0617	Multisystem	F	Live birth
EGAN00001366467	PP0623	Multisystem	F	Live birth
EGAN00001366473	PP0626	Multisystem	M	Live birth
EGAN00001366479	PP0629	Multisystem	F	Live birth
EGAN00001366503	PP0647	Multisystem	F	Live birth
EGAN00001366515	PP0659	Multisystem	M	Live birth
EGAN00001366536	PP0680	Multisystem	M	Live birth
EGAN00001366440	PP0701	Multisystem	M	Live birth
EGAN00001366458	PP0710	Multisystem	F	Live birth
EGAN00001366463	PP0713	Multisystem	F	Live birth
EGAN00001366576	PP0795	Multisystem	F	Live birth
EGAN00001402436	PP0948	Multisystem	F	Live birth
EGAN00001402421	PP0984	Multisystem	M	Live birth
EGAN00001402424	PP1044	Multisystem	M	Live birth
EGAN00001366585	PP1351	Multisystem	F	Live birth
EGAN00001366624	PP1390	Multisystem	M	Live birth
EGAN00001366627	PP1393	Multisystem	F	Live birth
EGAN00001410155	PP1408	Multisystem	M	Live birth
EGAN00001402427	PP1420	Multisystem	F	Live birth
EGAN00001410173	PP1462	Multisystem	M	Live birth
EGAN00001401997	PP1522	Multisystem	F	Live birth
EGAN00001402009	PP1534	Multisystem	F	Live birth
EGAN00001402104	PP1621	Multisystem	F	Live birth
EGAN00001402442	PP1627	Multisystem	F	Live birth
EGAN00001402119	PP1636	Multisystem	M	Live birth
EGAN00001402131	PP1648	Multisystem	M	Live birth
EGAN00001402137	PP1654	Multisystem	M	Live birth
EGAN00001402140	PP1657	Multisystem	F	Live birth
EGAN00001402146	PP1663	Multisystem	M	Live birth
EGAN00001402161	PP1678	Multisystem	M	Live birth
EGAN00001402164	PP1681	Multisystem	M	Live birth
EGAN00001402170	PP1687	Multisystem	M	Live birth
EGAN00001402179	PP1696	Multisystem	F	Live birth
EGAN00001402409	PP1738	Multisystem	F	Live birth
EGAN00001410044	PP1753	Multisystem	F	Live birth
EGAN00001410047	PP1756	Multisystem	M	Live birth
EGAN00001410059	PP1768	Multisystem	F	Live birth
EGAN00001410071	PP1780	Multisystem	M	Live birth
EGAN00001410077	PP1786	Multisystem	M	Live birth
EGAN00001410086	PP1795	Multisystem	M	Live birth
EGAN00001410104	PP1813	Multisystem	F	Live birth
EGAN00001410116	PP1825	Multisystem	F	Live birth
EGAN00001410158	PP1861	Multisystem	F	Live birth
EGAN00001410176	PP1876	Multisystem	F	Live birth
EGAN00001428004	PP1895	Multisystem	M	Live birth
EGAN00001428010	PP1901	Multisystem	M	Live birth
EGAN00001428013	PP1904	Multisystem	M	Live birth
EGAN00001428070	PP1961	Multisystem	M	Live birth
EGAN00001428076	PP1967	Multisystem	M	Live birth

EGAN00001428115	PP2006	Multisystem	M	Live birth
EGAN00001428124	PP2015	Multisystem	F	Live birth
EGAN00001428145	PP2036	Multisystem	M	Live birth
EGAN00001428151	PP2042	Multisystem	M	Live birth
EGAN00001428169	PP2060	Multisystem	M	Live birth
EGAN00001589007	PP2084	Multisystem	F	Live birth
EGAN00001471949	PP2099	Multisystem	F	Live birth
EGAN00001471888	PP2159	Multisystem	F	Live birth
EGAN00001472141	PP2351	Multisystem	M	Live birth
EGAN00001471892	PP2378	Multisystem	F	Live birth
EGAN00001471925	PP2396	Multisystem	F	Live birth
EGAN00001471958	PP2399	Multisystem	F	Live birth
EGAN00001471961	PP2402	Multisystem	M	Live birth
EGAN00001471904	PP2480	Multisystem	F	Live birth
EGAN00001471910	PP2489	Multisystem	F	Live birth
EGAN00001472072	PP2492	Multisystem	M	Live birth
EGAN00001471940	PP2501	Multisystem	M	Live birth
EGAN00001471973	PP2528	Multisystem	M	Live birth
EGAN00001472111	PP2621	Multisystem	F	Live birth
EGAN00001472132	PP2624	Multisystem	M	Live birth
EGAN00001472060	PP2630	Multisystem	F	Live birth
EGAN00001472063	PP2633	Multisystem	M	Live birth
EGAN00001471952	PP2645	Multisystem	M	Live birth
EGAN00001471864	PP2687	Multisystem	F	Live birth
EGAN00001471882	PP2705	Multisystem	M	Live birth
EGAN00001471991	PP2718	Multisystem	M	Live birth
EGAN00001472009	PP2736	Multisystem	M	Live birth
EGAN00001472024	PP2751	Multisystem	M	Live birth
EGAN00001472048	PP2772	Multisystem	F	Live birth
EGAN00001472117	PP2811	Multisystem	F	Live birth
EGAN00001472114	PP2862	Multisystem	F	Live birth
EGAN00001472099	PP2892	Multisystem	M	Live birth
EGAN00001476462	PP2913	Multisystem	F	Live birth
EGAN00001476489	PP2940	Multisystem	F	Live birth
EGAN00001476504	PP2955	Multisystem	M	Live birth
EGAN00001476510	PP2961	Multisystem	F	Live birth
EGAN00001476513	PP2964	Multisystem	F	Live birth
EGAN00001476522	PP2973	Multisystem	M	Live birth
EGAN00001476527	PP2979	Multisystem	M	Live birth
EGAN00001476530	PP2982	Multisystem	F	Live birth
EGAN00001543704	PP3018	Multisystem	M	Live birth
EGAN00001543696	PP3042	Multisystem	M	Live birth
EGAN00001589013	PP3045	Multisystem	M	Live birth
EGAN00001589022	PP3168	Multisystem	F	Live birth
EGAN00001589019	PP3186	Multisystem	M	Live birth
EGAN00001543733	PP3261	Multisystem	F	Live birth
EGAN00001598958	PP3273	Multisystem	M	Live birth
EGAN00001598925	PP3282	Multisystem	M	Live birth
EGAN00001588983	PP3384	Multisystem	M	Live birth
EGAN00001588974	PP3387	Multisystem	M	Live birth

EGAN00001588971	PP3513	Multisystem	M	Live birth
EGAN00001589016	PP3522	Multisystem	M	Live birth
EGAN00001588962	PP3573	Multisystem	M	Live birth
EGAN00001598922	PP3597	Multisystem	F	Live birth
EGAN00001598952	PP3615	Multisystem	M	Live birth
EGAN00001598973	PP3627	Multisystem	M	Live birth
EGAN00001598985	PP3636	Multisystem	M	Live birth
EGAN00001599000	PP3651	Multisystem	M	Live birth
EGAN00001599006	PP3657	Multisystem	M	Live birth
EGAN00001543707	PP0981	Multisystem	F	Unknown
EGAN00001543736	PP1206	Multisystem	F	Unknown
EGAN00001543729	PP3255	Multisystem	M	Unknown
EGAN00001543731	PP3258	Multisystem	F	Unknown
EGAN00001476541	PP1952	Multisystem	M	Unknown
EGAN00001472042	PP2766	Multisystem	F	Unknown
EGAN00001366512	PP0656	Multisystem	M	Unknown
EGAN00001589031	PP3339	Multisystem	F	Unknown
EGAN00001598964	PP3423	Multisystem	F	Unknown
EGAN00001598928	PP3600	Multisystem	M	Unknown
EGAN00001598970	PP3624	Multisystem	M	Unknown
EGAN00001366774	PP0207	Renal	M	Live birth
EGAN00001366791	PP0252	Renal	M	Live birth
EGAN00001366969	PP0351	Renal	M	Live birth
EGAN00001367002	PP0369	Renal	M	Live birth
EGAN00001366486	PP0458	Renal	M	Live birth
EGAN00001367095	PP0510	Renal	F	Live birth
EGAN00001367149	PP0564	Renal	M	Live birth
EGAN00001402079	PP1375	Renal	M	Live birth
EGAN00001402024	PP1552	Renal	M	Live birth
EGAN00001402036	PP1567	Renal	F	Live birth
EGAN00001410056	PP1765	Renal	M	Live birth
EGAN00001428160	PP2051	Renal	M	Live birth
EGAN00001472027	PP2754	Renal	M	Live birth
EGAN00001472129	PP2814	Renal	M	Live birth
EGAN00001589004	PP3111	Renal	M	Live birth
EGAN00001598976	PP3630	Renal	M	Live birth
EGAN00001472054	PP2823	Skeletal	M	Live birth
EGAN00001543709	PP3093	Skeletal	M	Live birth
EGAN00001530991	PP3132	Skeletal	M	Live birth
EGAN00001530988	PP3135	Skeletal	M	Live birth
EGAN00001543687	PP3195	Skeletal	F	Live birth
EGAN00001543726	PP3219	Skeletal	M	Live birth
EGAN00001543741	PP3264	Skeletal	M	Live birth
EGAN00001531047	PP3330	Skeletal	M	Live birth
EGAN00001531050	PP3333	Skeletal	F	Live birth
EGAN00001598979	PP3411	Skeletal	F	Live birth
EGAN00001588959	PP3540	Skeletal	F	Live birth
EGAN00001598931	PP3603	Skeletal	F	Live birth
EGAN00001598946	PP3609	Skeletal	F	Live birth
EGAN00001402152	PP1669	Skeletal	F	Miscarriage

EGAN00001402012	PP1537	Skeletal	F	NND
EGAN00001543721	PP3252	Skeletal	F	Unknown
EGAN00001367179	PP0594	Skeletal	M	Unknown
EGAN00001543685	PP3246	Skeletal	M	Unknown
EGAN00001543702	PP3249	Skeletal	M	Unknown
EGAN00001598967	PP3621	Skeletal	M	Unknown
EGAN00001366413	PP0087	Skeletal	F	Unknown
EGAN00001366664	PP0114	Skeletal	M	Unknown
EGAN00001366667	PP0117	Skeletal	M	Unknown
EGAN00001366670	PP0120	Skeletal	M	Unknown
EGAN00001366679	PP0129	Skeletal	M	Unknown
EGAN00001366714	PP0165	Skeletal	M	Unknown
EGAN00001366768	PP0204	Skeletal	F	Unknown
EGAN00001366776	PP0208	Skeletal	F	Unknown
EGAN00001366858	PP0309	Skeletal	F	Unknown
EGAN00001366867	PP0318	Skeletal	F	Unknown
EGAN00001366985	PP0360	Skeletal	M	Unknown
EGAN00001367027	PP0381	Skeletal	M	Unknown
EGAN00001366883	PP0419	Skeletal	F	Unknown
EGAN00001366490	PP0485	Skeletal	M	Unknown
EGAN00001367122	PP0537	Skeletal	M	Unknown
EGAN00001367131	PP0546	Skeletal	M	Unknown
EGAN00001367161	PP0576	Skeletal	F	Unknown
EGAN00001366500	PP0644	Skeletal	M	Unknown
EGAN00001366552	PP0792	Skeletal	F	Unknown
EGAN00001366546	PP1324	Skeletal	M	Unknown
EGAN00001366570	PP1342	Skeletal	M	Unknown
EGAN00001366579	PP1348	Skeletal	M	Unknown
EGAN00001366591	PP1357	Skeletal	M	Unknown
EGAN00001402394	PP1447	Skeletal	M	Unknown
EGAN00001402015	PP1540	Skeletal	M	Unknown
EGAN00001402030	PP1561	Skeletal	F	Unknown
EGAN00001402045	PP1576	Skeletal	F	Unknown
EGAN00001402054	PP1588	Skeletal	F	Unknown
EGAN00001402122	PP1639	Skeletal	M	Unknown
EGAN00001402125	PP1642	Skeletal	M	Unknown
EGAN00001402134	PP1651	Skeletal	F	Unknown
EGAN00001428043	PP1934	Skeletal	M	Unknown
EGAN00001428058	PP1949	Skeletal	F	Unknown
EGAN00001428085	PP1976	Skeletal	F	Unknown
EGAN00001428094	PP1985	Skeletal	M	Unknown
EGAN00001428097	PP1988	Skeletal	M	Unknown
EGAN00001428118	PP2009	Skeletal	M	Unknown
EGAN00001543738	PP2141	Skeletal	M	Unknown
EGAN00001471922	PP2543	Skeletal	M	Unknown
EGAN00001472051	PP2627	Skeletal	M	Unknown
EGAN00001472066	PP2636	Skeletal	M	Unknown
EGAN00001471891	PP2711	Skeletal	F	Unknown
EGAN00001471997	PP2724	Skeletal	M	Unknown
EGAN00001472021	PP2748	Skeletal	M	Unknown

EGAN00001472033	PP2760	Skeletal	F	Unknown
EGAN00001366649	PP0099	Spinal	M	Unknown
EGAN00001366652	PP0102	Spinal	F	Unknown
EGAN00001366702	PP0153	Spinal	M	Unknown
EGAN00001366873	PP0321	Spinal	F	Unknown
EGAN00001366527	PP0671	Spinal	M	Unknown
EGAN00001402066	PP1603	Spinal	F	Unknown
EGAN00001402069	PP1606	Spinal	M	Unknown
EGAN00001410128	PP1837	Spinal	M	Unknown
EGAN00001476453	PP2904	Spinal	M	Unknown
EGAN00001476468	PP2919	Spinal	F	Unknown

Supplementary Table 7: Details of cases with non-diagnostic but potentially clinically relevant variants

EGA_ID	PP_ID	Phenotypic Class	Gene	Variant type	Inheritance	Zygosity
EGAN00001366679	PP0129	Skeletal	FLNA	missense_variant	inherited from unaffected mother (X)	Homozygous
EGAN00001366482	PP0722	Large NT>4.0	KMT2D	missense_variant	de novo	Heterozygous
EGAN00001366603	PP1369	Large NT>4.0	NRAS	missense_variant	de novo	Heterozygous
EGAN00001366861	PP0312	Large NT>4.0	KIAA0586	missense_variant	inherited	Homozygous
EGAN00001367002	PP0369	Renal	FLNA	missense_variant	inherited from unaffected mother (X)	Homozygous
EGAN00001401997	PP1522	Multisystem	RECQL4	missense/stop gained	inherited	Compound heterozygous
EGAN00001402003	PP1528	Large NT>4.0	PTPN11	missense_variant	de novo	Heterozygous
EGAN00001366931	PP0425	Cardiac	HYDIN	missense variant/missense variant	inherited	Compound heterozygous
EGAN00001402367	PP1720	Brain	CHD7	stop_gained	de novo	Heterozygous
EGAN00001402370	PP1723	Large NT>4.0	KMT2A	frameshift_variant	de novo	Heterozygous
EGAN00001366546	PP1324	Skeletal	TUBA1A	missense_variant	de novo	Heterozygous
EGAN00001366636	PP1396	Brain	MBTPS2	missense_variant	inherited from unaffected mother (X)	Homozygous
EGAN00001471867	PP2690	Brain	MECP2	missense variant	inherited from unaffected mother (X)	Heterozygous

EGAN00001472069	PP2886	Cardiac	PROK2	splice_acceptor_variant	inherited from similarly affected mother	Heterozygous
EGAN00001402021	PP1549	Cardiac	ROBO1	splice_acceptor_variant	de novo	Heterozygous
EGAN00001367170	PP0585	Large NT>4.0	ROBO1	frameshift_variant	de novo	Heterozygous
EGAN00001531002	PP3144	Abdominal	ATRX	missense_variant	inherited from unaffected mother (X)	Hemizygous
EGAN00001598937	PP3285	Cardiac	ACTB	missense_variant	de novo	Heterozygous
EGAN00001588977	PP3474	Brain	KCNQ2	missense_variant	de novo	Heterozygous
EGAN00001589013	PP3045	Multisystem	BCS1L	missense variant/missense variant	inherited	Compound heterozygous
EGAN00001531035	PP3318	Chest	PACS1	missense_variant	de novo	Heterozygous
EGAN00001471958	PP2399	Multisystem	NSD1	missense_variant	de novo	Heterozygous
EGAN00001428160	PP2051	Renal	FOXF1|FOXC2	Deletion (183kb)	de novo	Heterozygous
EGAN00001366772	PP0206	Large NT>4.0	SLC9A6	Deletion (375bp)	de novo	Hemizygous

Supplementary Table 8: Percent of protein coding region covered by >=13X for DDG2P gene panel.

chromosome	start	stop	gene	hgnc_id	Percent of protein coding region covered by >=13X
1	1167629	1170421	B3GALT6	17978	72.42
1	1167629	1170421	B3GALT6	17978	72.42
1	1270656	1284730	DVL1	3084	100.00
1	1447531	1470067	ATAD3A	25567	87.56
1	1447531	1470067	ATAD3A	25567	87.56
1	1716729	1822495	GNB1	4396	100.00
1	2160134	2241558	SKI	10896	100.00
1	2336236	2345236	PEX10	8851	95.94
1	2336236	2345236	PEX10	8851	95.94
1	2336236	2345236	PEX10	8851	95.94
1	3728645	3773778	CEP104	24866	98.92
1	5922871	6052533	NPHP4	19104	98.78
1	6845384	7829766	CAMTA1	18806	100.00
1	8377886	8404227	SLC45A1	17939	100.00
1	8412457	8877702	RERE	9965	96.04
1	10003486	10045559	NMNAT1	17877	99.29
1	10532345	10690815	PEX14	8856	98.28
1	10532345	10690815	PEX14	8856	98.28
1	11166592	11322564	MTOR	3942	100.00
1	11845780	11866977	MTHFR	7436	100.00
1	11994262	12035595	PLOD1	9081	99.12
1	16370272	16383803	CLCNKB	2027	96.03
1	17312453	17338423	ATP13A2	30213	100.00
1	19197926	19229275	ALDH4A1	406	99.16
1	19542158	19578046	EMC1	28957	100.00
1	19542158	19578046	EMC1	28957	100.00
1	20978270	20988000	DDOST	2728	100.00
1	21835858	21904905	ALPL	438	100.00
1	22148738	22263790	HSPG2	5273	96.19
1	22148738	22263790	HSPG2	5273	96.19
1	22443798	22470462	WNT4	12783	92.71
1	22443798	22470462	WNT4	12783	92.71
1	23345941	23410182	KDM1A	29079	100.00
1	24122089	24127271	GALE	4116	100.00
1	24128375	24165110	HMGCL	5005	100.00
1	24171567	24194784	FUCA1	4006	100.00
1	24645812	24690972	GRHL3	25839	100.00
1	26758773	26797785	DHDDS	20603	100.00
1	27022524	27108595	ARID1A	11110	96.43
1	27113963	27124889	PIGV	26031	100.00
1	27860546	27930942	AHDC1	25230	99.94
1	29519385	29557454	MECR	19691	100.00
1	33473585	33546597	AK2	362	93.06
1	40420802	40435638	MFSD2A	25897	100.00
1	40538379	40563375	PPT1	9325	100.00
1	40723779	40759856	ZMPSTE24	12877	99.37

1	40723779	40759856	ZMPSTE24	12877	99.37
1	40766159	40783488	COL9A2	2218	97.52
1	40766159	40783488	COL9A2	2218	97.52
1	43198764	43205925	CLDN19	2040	96.89
1	43212006	43232755	P3H1	19316	100.00
1	43391052	43424530	SLC2A1	11005	100.00
1	43391052	43424530	SLC2A1	11005	100.00
1	43855553	43918321	SZT2	29040	99.81
1	44171495	44396831	ST3GAL3	10866	100.00
1	44457172	44497139	SLC6A9	11056	100.00
1	45805342	45809647	TOE1	15954	100.00
1	45965725	45976739	MMACHC	24525	100.00
1	46654354	46685977	POMGNT1	19139	100.00
1	46654354	46685977	POMGNT1	19139	100.00
1	46654354	46685977	POMGNT1	19139	100.00
1	47715811	47779819	STIL	10879	100.00
1	47881744	47883723	FOXE3	3808	71.25
1	47881744	47883723	FOXE3	3808	71.25
1	52838501	52870131	ORC1	8487	100.00
1	55315306	55352891	DHCR24	2859	100.00
1	55464606	55476556	BSND	16512	100.00
1	62920399	63153969	DOCK7	19190	100.00
1	63833261	63904233	ALG6	23157	92.18
1	64058947	64125916	PGM1	8905	100.00
1	67465015	67519782	SLC35D1	20800	100.00
1	68894505	68915642	RPE65	10294	100.00
1	76190036	76253260	ACADM	89	100.00
1	92711959	92764544	GLMN	14373	95.07
1	100315640	100389579	AGL	321	99.83
1	100652475	100715390	DBT	2698	96.55
1	103342023	103574052	COL11A1	2186	100.00
1	103342023	103574052	COL11A1	2186	100.00
1	108676658	108743471	SLC25A24	20662	100.00
1	109417972	109477167	GPSM2	29501	98.42
1	109605108	109618624	TAF13	11546	100.00
1	110091233	110136975	GNAI3	4387	92.74
1	110158726	110174673	AMPD2	469	98.72
1	110602616	110613322	ALX3	449	89.53
1	110693108	110744824	SLC6A17	31399	100.00
1	111136202	111174096	KCNA2	6220	100.00
1	111136202	111174096	KCNA2	6220	100.00
1	113615831	113674882	LRIG2	20889	100.00
1	114437370	114447823	AP4B1	572	97.21
1	115247090	115259515	NRAS	7989	100.00
1	115572415	115576941	TSHB	12372	100.00
1	119425669	119532179	TBX15	11594	100.00
1	120202421	120286838	PHGDH	8923	99.17
1	120202421	120286838	PHGDH	8923	99.17
1	120290619	120311528	HMGCS2	5008	99.08
1	120454176	120612240	NOTCH2	7882	98.79

1	145507598	145513536	RBM8A	9905	96.00
1	145516252	145523730	PEX11B	8853	100.00
1	147374946	147381393	GJA8	4281	100.00
1	147374946	147381393	GJA8	4281	100.00
1	149895209	149900236	SF3B4	10771	97.80
1	150768684	150780799	CTSK	2536	100.00
1	150980896	151008189	PRUNE1	13420	100.00
1	151375200	151431941	POGZ	18801	98.57
1	153777201	153895451	GATAD2B	30778	100.00
1	154244987	154248351	HAX1	16915	100.00
1	154540257	154552502	CHRNB2	1962	100.00
1	154540257	154552502	CHRNB2	1962	100.00
1	154554538	154600475	ADAR	225	100.00
1	154554538	154600475	ADAR	225	100.00
1	154554538	154600475	ADAR	225	100.00
1	154955814	154965587	FLAD1	24671	100.00
1	155112367	155113071	DPM3	3007	100.00
1	155204243	155214490	GBA	4177	100.00
1	155204243	155214490	GBA	4177	100.00
1	155204243	155214490	GBA	4177	100.00
1	155204243	155214490	GBA	4177	100.00
1	155204243	155214490	GBA	4177	100.00
1	155204243	155214490	GBA	4177	100.00
1	155867599	155881195	RIT1	10023	100.00
1	156052364	156109880	LMNA	6636	90.23
1	156052364	156109880	LMNA	6636	90.23
1	156052364	156109880	LMNA	6636	90.23
1	156052364	156109880	LMNA	6636	90.23
1	156052364	156109880	LMNA	6636	90.23
1	156052364	156109880	LMNA	6636	90.23
1	156052364	156109880	LMNA	6636	90.23
1	156052364	156109880	LMNA	6636	90.23
1	156052364	156109880	LMNA	6636	90.23
1	156052364	156109880	LMNA	6636	90.23
1	156052364	156109880	LMNA	6636	90.23
1	156561554	156564091	NAXE	18453	100.00
1	156785432	156851642	NTRK1	8031	100.00
1	160007257	160040038	KCNJ10	6256	98.51
1	160246602	160256138	PEX19	9713	99.00
1	160246602	160256138	PEX19	9713	99.00
1	161040785	161059389	NECTIN4	19688	99.15
1	162601163	162757190	DDR2	2731	98.85
1	165696032	165796992	TMCO1	18188	100.00
1	170501270	170522587	GORAB	25676	100.00
1	173793641	173827684	DARS2	25538	100.00
1	179519674	179545087	NPHS2	13394	97.48
1	180199421	180249380	LHX4	21734	100.00
1	182350839	182361341	GLUL	4341	97.59
1	184020811	184043346	TSEN15	16791	90.70
1	197053258	197115824	ASPM	19048	97.78

1	197170592	197447585	CRB1	2343	100.00
1	197170592	197447585	CRB1	2343	100.00
1	200593024	200639097	DDX59	25360	100.00
1	202300785	202311108	UBE2T	25009	100.00
1	202696526	202778598	KDM5B	18039	100.00
1	203830731	203839678	SNRPE	11161	89.96
1	205111632	205180727	DSTYK	29043	97.96
1	209959036	209979465	IRF6	6121	95.37
1	209959036	209979465	IRF6	6121	95.37
1	210856555	211307457	KCNH1	6250	99.88
1	213031597	213072705	FLVCR1	24682	98.14
1	214522039	214725792	PTPN14	9647	99.76
1	218519577	218617961	TGFB2	11768	99.92
1	220321635	220445796	RAB3GAP2	17168	94.32
1	224572845	224624735	WDR26	21208	97.03
1	225589204	225616627	LBR	6518	95.13
1	226107578	226111978	PYCR2	30262	98.96
1	227085237	227175246	COQ8A	16812	100.00
1	228337553	228347527	GJC2	17494	90.38
1	228337553	228347527	GJC2	17494	90.38
1	228337553	228347527	GJC2	17494	90.38
1	229566992	229569845	ACTA1	129	98.85
1	231376953	231413719	GNPAT	4416	96.57
1	235530675	235612283	TBCE	11582	100.00
1	235530675	235612283	TBCE	11582	100.00
1	235530675	235612283	TBCE	11582	100.00
1	235613238	235667781	B3GALNT2	28596	97.67
1	235824341	236046940	LYST	1968	98.17
1	236958610	237067281	MTR	7468	100.00
1	240177648	240638489	FMN2	14074	83.92
1	241660903	241683061	FH	3700	86.30
1	243419320	243663394	SDCCAG8	10671	100.00
1	243651535	244014381	AKT3	393	76.74
1	244214585	244220778	ZBTB18	13030	100.00
1	245014468	245027844	HNRNPU	5048	99.11
2	1635659	1748624	PXDN	14966	98.75
2	1792885	2335032	MYT1L	7623	99.64
2	3383446	3488865	TRAPPC12	24284	100.00
2	3642426	3692048	COLEC11	17213	100.00
2	5832799	5841516	SOX11	11191	100.00
2	8865408	8977760	KIDINS220	29508	99.87
2	15307032	15701454	NBAS	15625	100.00
2	16080686	16087129	MYCN	7559	100.00
2	20110021	20189892	WDR35	29250	100.00
2	20110021	20189892	WDR35	29250	100.00
2	20191872	20212455	MATN3	6909	84.46
2	25455845	25565459	DNMT3A	2978	96.09
2	25956622	26101385	ASXL2	23805	100.00
2	26413504	26467594	HADHA	4801	87.46
2	26624784	26679579	DRC1	24245	100.00

2	27440258	27466811	CAD	1424		96.91
2	27532360	27548547	MPV17	7224		100.00
2	27667238	27712656	IFT172	30391		100.00
2	27667238	27712656	IFT172	30391		100.00
2	28974506	29025806	PPP1CB	9282		99.90
2	29283842	29297127	C2orf71	34383		98.42
2	38294116	38337044	CYP1B1	2597		100.00
2	39208537	39351486	SOS1	11187		99.15
2	44113647	44223144	LRPPRC	15714		100.00
2	44543420	44589001	PREPL	30228		100.00
2	45168902	45173216	SIX3	10889		99.70
2	47143296	47303276	TTC7A	19750		98.83
2	55861400	55921045	PNPT1	23166		94.59
2	55861400	55921045	PNPT1	23166		94.59
2	58386378	58468507	FANCL	20748		100.00
2	58386378	58468507	FANCL	20748		100.00
2	60678302	60780702	BCL11A	13221		100.00
2	61244360	61279125	PEX13	8855		100.00
2	61244360	61279125	PEX13	8855		100.00
2	62051989	62081278	FAM161A	25808		98.79
2	63348518	64054977	WDPCP	28027		96.21
2	69240310	69476459	ANTXR1	21014		93.64
2	69622882	69664760	NFU1	16287		86.77
2	71163012	71192536	ATP6V1B1	853		100.00
2	71336814	71357369	MCEE	16732		100.00
2	73114489	73119287	SPR	11257		100.00
2	73612886	73837920	ALMS1	428		97.00
2	74056086	74100786	STAMBP	16950		99.33
2	74362525	74375121	BOLA3	24415		83.02
2	74688184	74692537	MOGS	24862		99.72
2	84650647	84687169	SUCLG1	11449		100.00
2	86066267	86116137	ST3GAL5	10872		91.57
2	86247339	86333278	POLR1A	17264		100.00
2	88856259	88927094	EIF2AK3	3255		83.38
2	99771418	99779620	LIPT1	29569		100.00
2	100162323	100759201	AFF3	6473		100.00
2	108602979	108630450	SLC5A7	14025		100.00
2	110879888	110962643	NPHP1	7905		100.00
2	110879888	110962643	NPHP1	7905		100.00
2	110879888	110962643	NPHP1	7905		100.00
2	113493930	113522254	CKAP2L	26877		90.89
2	113973574	114036527	PAX8	8622		99.78
2	121493199	121750229	GLI2	4318		99.12
2	122288457	122288583	RNU4ATAC	34016	No protein	
2	127805603	127864931	BIN1	1052		100.00
2	128014866	128051752	ERCC3	3435		93.13
2	128014866	128051752	ERCC3	3435		93.13
2	131095814	131099922	CCDC115	28178		84.71
2	131350339	131357123	CFC1	18292		69.64
2	135809835	135933964	RAB3GAP1	17063		100.00

2	136664247	136743670	DARS	2678	100.00
2	145141648	145282147	ZEB2	14881	100.00
2	148687968	148779147	ORC4	8490	89.69
2	149632819	149883273	KIF5C	6325	99.27
2	150426148	150444330	MMADHC	25221	81.26
2	152341850	152591001	NEB	7720	82.11
2	158592958	158732374	ACVR1	171	100.00
2	162272605	162282381	TBR1	11590	100.00
2	163123589	163175213	IFIH1	18873	96.13
2	163123589	163175213	IFIH1	18873	96.13
2	165944032	166060577	SCN3A	10590	99.65
2	166095912	166248818	SCN2A	10588	95.41
2	166095912	166248818	SCN2A	10588	95.41
2	166095912	166248818	SCN2A	10588	95.41
2	166845670	166984523	SCN1A	10585	99.22
2	169779448	169887832	ABCB11	42	98.59
2	169983619	170219195	LRP2	6694	100.00
2	170335688	170382432	BBS5	970	94.05
2	175612320	175629200	CHRNA1	1955	100.00
2	176957619	176960666	HOXD13	5136	100.00
2	176957619	176960666	HOXD13	5136	100.00
2	176957619	176960666	HOXD13	5136	100.00
2	176957619	176960666	HOXD13	5136	100.00
2	176957619	176960666	HOXD13	5136	100.00
2	176957619	176960666	HOXD13	5136	100.00
2	178257372	178408564	AGPS	327	100.00
2	191054461	191208919	HIBCH	4908	78.21
2	191829084	191885686	STAT1	11362	96.40
2	191829084	191885686	STAT1	11362	96.40
2	191829084	191885686	STAT1	11362	96.40
2	197059094	197458416	HECW2	29853	100.00
2	198351305	198381461	HSPD1	5261	89.98
2	200134223	200335989	SATB2	21637	98.90
2	200134223	200335989	SATB2	21637	98.90
2	200134223	200335989	SATB2	21637	98.90
2	202484907	202508293	TMEM237	14432	100.00
2	202565277	202645912	ALS2	443	100.00
2	206979541	207024327	NDUFS1	7707	100.00
2	206979541	207024327	NDUFS1	7707	100.00
2	208627310	208634287	FZD5	4043	100.00
2	208986331	208989225	CRYGD	2411	100.00
2	208986331	208989225	CRYGD	2411	100.00
2	208992861	208994554	CRYGC	2410	98.48
2	210636717	210864024	UNC80	26582	100.00
2	211342406	211543831	CPS1	2323	100.00
2	216176540	216214487	ATIC	794	98.93
2	216225163	216300895	FN1	3778	97.76
2	217277137	217347776	SMARCAL1	11102	99.40
2	219523487	219528166	BCS1L	1020	100.00
2	219919142	219925189	IHH	5956	100.00

2	219919142	219925189	IHH	5956	100.00
2	220299568	220363009	SPEG	16901	100.00
2	220363589	220371710	GMPPA	22923	100.00
2	220415451	220436581	OBSL1	29092	99.12
2	223064607	223163715	PAX3	8617	100.00
2	223064607	223163715	PAX3	8617	100.00
2	227867427	228028829	COL4A4	2206	99.67
2	228029281	228179508	COL4A3	2204	100.00
2	228029281	228179508	COL4A3	2204	100.00
2	228549926	228582728	SLC19A3	16266	100.00
2	230628554	230787955	TRIP12	12306	100.00
2	232063260	232239548	ARMC9	20730	99.41
2	232825955	233209060	DIS3L2	28648	100.00
2	233344537	233352538	ECEL1	3147	100.00
2	233385173	233390422	PRSS56	39433	91.07
2	233404437	233411113	CHRNG	1967	100.00
2	234526291	234681956	UGT1A1	12530	100.00
2	238232646	238323018	COL6A3	2213	99.91
2	238232646	238323018	COL6A3	2213	99.91
2	239756673	239795893	TWIST2	20670	100.00
2	239756673	239795893	TWIST2	20670	100.00
2	239969864	240323348	HDAC4	14063	99.11
2	240831867	240964819	NDUFA10	7684	100.00
2	241653181	241759725	KIF1A	888	100.00
2	241653181	241759725	KIF1A	888	100.00
2	241653181	241759725	KIF1A	888	100.00
2	241807896	241819919	AGXT	341	100.00
3	3742498	4508965	SUMF1	20376	97.96
3	4535032	4889524	ITPR1	6180	100.00
3	4535032	4889524	ITPR1	6180	100.00
3	4535032	4889524	ITPR1	6180	100.00
3	4535032	4889524	ITPR1	6180	100.00
3	9439299	9520924	SETD5	25566	99.31
3	9773413	9789702	BRPF1	14255	100.00
3	9932238	9936033	JAGN1	26926	100.00
3	9975506	9987097	CRELD1	14630	100.00
3	10068098	10143614	FANCD2	3585	100.00
3	11034410	11080933	SLC6A1	11042	100.00
3	12525931	12581122	TSEN2	28422	99.64
3	12625100	12705725	RAF1	9829	100.00
3	13857755	13921618	WNT7A	12786	100.00
3	13857755	13921618	WNT7A	12786	100.00
3	14186647	14220283	XPC	12816	99.89
3	15642848	15687329	BTD	1122	100.00
3	25215823	25639423	RARB	9865	100.00
3	25215823	25639423	RARB	9865	100.00
3	25760435	25831530	NGLY1	17646	100.00
3	30647994	30735634	TGFBR2	11773	100.00
3	30647994	30735634	TGFBR2	11773	100.00
3	33038100	33138722	GLB1	4298	95.92

3	33038100	33138722	GLB1	4298	95.92
3	33038100	33138722	GLB1	4298	95.92
3	33038100	33138722	GLB1	4298	95.92
3	38887260	38992052	SCN11A	10583	95.86
3	39424839	39438842	SLC25A38	26054	95.89
3	41236328	41301587	CTNNB1	2514	100.00
3	42727011	42734036	KLHL40	30372	100.00
3	43120724	43147568	POMGNT2	25902	100.00
3	43731605	43775863	ABHD5	21396	100.00
3	45429998	45590913	LARS2	17095	100.00
3	45959396	46037316	FYCO1	14673	97.32
3	46919236	46945287	PTH1R	9608	100.00
3	46919236	46945287	PTH1R	9608	100.00
3	46919236	46945287	PTH1R	9608	100.00
3	46919236	46945287	PTH1R	9608	100.00
3	47844399	47891685	DHX30	16716	100.00
3	48506445	48509044	TREX1	12269	100.00
3	48894369	48936426	SLC25A20	1421	100.00
3	49067140	49131796	QRICH1	24713	98.88
3	49133365	49142553	QARS	9751	100.00
3	49454211	49460186	AMT	473	100.00
3	49506146	49573048	DAG1	2666	99.93
3	49754277	49761384	GMPPB	22932	100.00
3	49866034	49894007	TRAIP	30764	100.00
3	50337320	50349812	HYAL1	5320	100.00
3	50378541	50384283	ZMYND10	19412	100.00
3	52009066	52023213	ACY1	177	97.23
3	52109269	52188706	POC1A	24488	100.00
3	52109269	52188706	POC1A	24488	100.00
3	53122499	53164478	RFT1	30220	100.00
3	53258723	53290068	TKT	11834	96.47
3	53528683	53847760	CACNA1D	1391	100.00
3	53528683	53847760	CACNA1D	1391	100.00
3	55499743	55523973	WNT5A	12784	100.00
3	57231944	57260549	HESX1	4877	95.34
3	57231944	57260549	HESX1	4877	95.34
3	57994127	58157982	FLNB	3755	99.10
3	57994127	58157982	FLNB	3755	99.10
3	57994127	58157982	FLNB	3755	99.10
3	57994127	58157982	FLNB	3755	99.10
3	57994127	58157982	FLNB	3755	99.10
3	66119285	66438540	SLC25A26	20661	94.55
3	69024365	69063112	EOGT	28526	100.00
3	69788586	70017488	MITF	7105	100.00
3	69788586	70017488	MITF	7105	100.00
3	69788586	70017488	MITF	7105	100.00
3	69788586	70017488	MITF	7105	100.00
3	71003844	71633140	FOXP1	3823	100.00
3	87308554	87325737	POU1F1	9210	96.65
3	97483365	97519953	ARL6	13210	100.00

3	97483365	97519953	ARL6	13210	100.00
3	99979844	100044095	TBC1D23	25622	96.82
3	101280706	101285290	TRMT10C	26022	100.00
3	111011566	111384597	CD96	16892	100.00
3	114056941	114866118	ZBTB20	13503	100.00
3	119013220	119139561	ARHGAP31	29216	93.65
3	122044091	122060819	CSTA	2481	100.00
3	123328896	123603178	MYLK	7590	99.80
3	124449213	124464040	UMPS	12563	99.38
3	126200124	126236616	UROC1	26444	100.00
3	128198270	128212028	GATA2	4171	99.03
3	128598439	128634910	ACAD9	21497	100.00
3	129158968	129239198	IFT122	13556	99.12
3	132276986	132441303	NPHP3	7907	90.32
3	132276986	132441303	NPHP3	7907	90.32
3	132276986	132441303	NPHP3	7907	90.32
3	132373290	132396941	UBA5	23230	84.86
3	133118839	133194066	BFSP2	1041	96.88
3	134204585	134293859	CEP63	25815	99.58
3	135969148	136056738	PCCB	8654	100.00
3	136055077	136471220	STAG1	11354	91.56
3	138663066	138665982	FOXL2	1092	100.00
3	138724648	139076065	MRPS22	14508	98.47
3	142168077	142297668	ATR	882	98.56
3	145787227	145881440	PLOD2	9082	95.11
3	147111209	147228080	ZIC1	12872	100.00
3	155538813	155572218	SLC33A1	95	100.00
3	158362067	158410364	GFM1	13780	99.34
3	159974774	160117668	IFT80	29262	85.44
3	167401086	167452727	PDCD10	8761	100.00
3	168801287	169381406	MECOM	3498	100.00
3	170714137	170744539	SLC2A2	11006	100.00
3	176737143	176915261	TBL1XR1	29529	78.12
3	178865902	178957881	PIK3CA	8975	100.00
3	178865902	178957881	PIK3CA	8975	100.00
3	178865902	178957881	PIK3CA	8975	100.00
3	180320646	180588793	CCDC39	25244	94.32
3	181429714	181432221	SOX2	11195	100.00
3	181429714	181432221	SOX2	11195	100.00
3	182733006	182833863	MCCC1	6936	98.48
3	183873176	183891398	DVL3	3087	100.00
3	183960089	183967336	ALG3	23056	99.78
3	186935942	187009810	MASP1	6901	100.00
3	189349205	189615068	TP63	15979	100.00
3	189349205	189615068	TP63	15979	100.00
3	189349205	189615068	TP63	15979	100.00
3	189349205	189615068	TP63	15979	100.00
3	189349205	189615068	TP63	15979	100.00
3	189349205	189615068	TP63	15979	100.00
3	189349205	189615068	TP63	15979	100.00

3	191857184	192485553	FGF12	3668	97.81
3	195941093	196014828	PCYT1A	8754	100.00
4	492989	533985	PIGG	25985	98.68
4	980785	998316	IDUA	5391	90.32
4	980785	998316	IDUA	5391	90.32
4	980785	998316	IDUA	5391	90.32
4	1341054	1381837	UVSSA	29304	100.00
4	1795034	1810599	FGFR3	3690	100.00
4	1795034	1810599	FGFR3	3690	100.00
4	1795034	1810599	FGFR3	3690	100.00
4	1795034	1810599	FGFR3	3690	100.00
4	1795034	1810599	FGFR3	3690	100.00
4	1795034	1810599	FGFR3	3690	100.00
4	1795034	1810599	FGFR3	3690	100.00
4	1795034	1810599	FGFR3	3690	100.00
4	4861393	4865663	MSX1	7391	100.00
4	5544499	5711275	EVC2	19747	94.57
4	5544499	5711275	EVC2	19747	94.57
4	5712924	5830772	EVC	3497	98.13
4	5712924	5830772	EVC	3497	98.13
4	8847802	8873543	HMX1	5017	72.40
4	13542454	13546674	NKX3-2	951	96.11
4	15471489	15603180	CC2D2A	29253	93.25
4	15471489	15603180	CC2D2A	29253	93.25
4	15471489	15603180	CC2D2A	29253	93.25
4	16162128	16229033	TAPT1	26887	100.00
4	17461884	17513857	QDPR	9752	100.00
4	26165077	26436541	RBPJ	5724	83.50
4	39184024	39287430	WDR19	18340	98.71
4	39184024	39287430	WDR19	18340	98.71
4	39460620	39479273	LIAS	16429	97.67
4	41746099	41750987	PHOX2B	9143	100.00
4	41746099	41750987	PHOX2B	9143	100.00
4	55524085	55606881	KIT	6342	99.78
4	56212276	56239263	SRD5A3	25812	97.70
4	56262124	56319564	TMEM165	30760	100.00
4	56815037	56899529	CEP135	29086	90.30
4	57896939	57976551	IGFBP7	5476	96.00
4	72053003	72437804	SLC4A4	11030	99.22
4	76481258	76491095	ODAPH	26300	100.00
4	77356253	77704406	SHROOM3	30422	100.00
4	78978724	79465423	FRAS1	19185	99.48
4	84182689	84206067	COQ2	25223	99.29
4	88529681	88538062	DSPP	3054	96.16
4	88529681	88538062	DSPP	3054	96.16
4	88529681	88538062	DSPP	3054	96.16
4	88571459	88585513	DMP1	2932	100.00
4	89442724	89442940	PIGY	28213	100.00
4	95679119	96079599	BMPR1B	1077	100.00
4	101944566	102269435	PPP3CA	9314	96.46

4	103172198	103352415	SLC39A8	20862	100.00
4	103552660	103682151	MANBA	6831	98.45
4	103790135	103810399	CISD2	24212	77.94
4	104507188	104640973	TACR3	11528	99.00
4	106290234	106395238	PPA2	28883	90.25
4	106962756	107242652	TBCK	28261	96.50
4	107236701	107270383	AIMP1	10648	95.45
4	108852525	108874613	CYP2U1	20582	96.15
4	108910870	108956331	HADH	4799	100.00
4	109731877	110223813	COL25A1	18603	97.25
4	110769358	110793471	LRIT3	24783	91.91
4	111538579	111563279	PITX2	9005	95.90
4	111538579	111563279	PITX2	9005	95.90
4	111538579	111563279	PITX2	9005	95.90
4	111538579	111563279	PITX2	9005	95.90
4	113558120	113578748	LARP7	24912	72.26
4	119201193	119274158	PRSS12	9477	99.23
4	119643978	119759838	SEC24D	10706	99.13
4	122745595	122791652	BBS7	18758	95.53
4	123073488	123283913	KIAA1109	26953	98.56
4	123653857	123666098	BBS12	26648	100.00
4	123844229	124240605	SPATA5	18119	100.00
4	126237554	126414087	FAT4	23109	100.00
4	128802016	128820350	PLK4	11397	97.08
4	128838960	128887150	MFSD8	28486	100.00
4	140222609	140341187	NAA15	30782	99.48
4	146539415	146581187	MMAA	18871	100.00
4	148402069	148466106	EDNRA	3179	100.00
4	151185594	151936879	LRBA	1742	100.00
4	151503077	151505843	MAB21L2	6758	100.00
4	151503077	151505843	MAB21L2	6758	100.00
4	155548097	155674270	LRAT	6685	100.00
4	159593277	159630775	ETFDH	3483	100.00
4	170314426	170533780	NEK1	7744	100.00
4	170314426	170533780	NEK1	7744	100.00
4	175411328	175444305	HPGD	5154	100.00
4	178351924	178363657	AGA	318	100.00
4	184580420	184634745	TRAPPC11	25751	100.00
4	186064395	186071536	SLC25A4	10990	100.00
5	218356	256815	SDHA	10680	73.73
5	892758	919472	TRIP13	12307	100.00
5	1253262	1295184	TERT	11730	92.84
5	1392909	1445545	SLC6A3	11049	100.00
5	6599352	6633404	NSUN2	25994	98.20
5	7851299	7906138	MTRR	7473	100.00
5	13690440	13944652	DNAH5	2950	100.00
5	14143811	14532235	TRIO	12303	98.07
5	14664773	14699820	OTULIN	25118	96.51
5	14704910	14871887	ANKH	15492	100.00
5	14704910	14871887	ANKH	15492	100.00

5	16473147	16617167	RETREG1	25964	96.10
5	36876861	37066515	NIPBL	28862	99.48
5	37106330	37249530	C5orf42	25801	95.18
5	41730167	41870621	OXCT1	8527	99.06
5	42423879	42721979	GHR	4263	100.00
5	44303646	44389808	FGF10	3666	100.00
5	45259349	45696253	HCN1	4845	99.81
5	52391509	52405893	MOCS2	7193	100.00
5	52856463	52979168	NDUFS4	7711	100.00
5	52856463	52979168	NDUFS4	7711	100.00
5	52856463	52979168	NDUFS4	7711	100.00
5	54526980	54529508	CCNO	18576	98.86
5	56111401	56191979	MAP3K1	6848	100.00
5	58264865	59817947	PDE4D	8783	99.47
5	60169658	60240900	ERCC8	3439	92.95
5	60240956	60448853	NDUFAF2	28086	79.41
5	60628100	60841997	ZSWIM6	29316	96.76
5	61601989	61833076	KIF2A	6318	100.00
5	64064757	64314590	CWC27	10664	99.18
5	67511548	67597649	PIK3R1	8979	97.07
5	67511548	67597649	PIK3R1	8979	97.07
5	70883115	70954531	MCCC2	6937	99.70
5	73935848	74018472	HEXB	4879	99.48
5	74664311	74807963	COL4A3BP	2205	91.88
5	78073032	78281910	ARSB	714	100.00
5	79922047	79950802	DHFR	2861	79.96
5	81569177	81574396	RPS23	10410	73.84
5	82373317	82649606	XRCC4	12831	100.00
5	86563705	86687748	RASA1	9871	96.55
5	86563705	86687748	RASA1	9871	96.55
5	88013975	88199922	MEF2C	6996	100.00
5	92919043	92930321	NR2F1	7975	100.00
5	94799599	94890711	TTC37	23639	100.00
5	118788138	118972894	HSD17B4	5213	98.79
5	118788138	118972894	HSD17B4	5213	98.79
5	125877533	125931110	ALDH7A1	877	89.21
5	126626523	126801429	MEGF10	29634	100.00
5	127593601	127994878	FBN2	3604	99.63
5	130494720	130507428	HINT1	4912	72.18
5	131705444	131731306	SLC22A5	10969	100.00
5	132202252	132203723	UQCRQ	29594	100.00
5	132211071	132299326	AFF4	17869	100.00
5	134363425	134370503	PITX1	9004	94.81
5	134363425	134370503	PITX1	9004	94.81
5	138282409	138629246	SIL1	24624	95.82
5	139487362	139496321	PURA	9701	100.00
5	149340300	149373018	SLC26A2	10994	100.00
5	149340300	149373018	SLC26A2	10994	100.00
5	149340300	149373018	SLC26A2	10994	100.00
5	149340300	149373018	SLC26A2	10994	100.00

5	149493400	149535435	PDGFRB	8804	96.63
5	149493400	149535435	PDGFRB	8804	96.63
5	149599054	149669854	CAMK2A	1460	100.00
5	149737202	149779871	TCOF1	11654	97.80
5	150591711	150650001	GM2A	4367	100.00
5	151040657	151066726	SPARC	11219	100.00
5	160715436	160976050	GABRB2	4082	100.00
5	161274197	161326975	GABRA1	4075	100.00
5	161274197	161326975	GABRA1	4075	100.00
5	161494546	161582542	GABRG2	4087	92.25
5	161494546	161582542	GABRG2	4087	92.25
5	171752185	171881527	SH3PXD2B	29242	100.00
5	172659112	172662360	NKX2-5	2488	100.00
5	172659112	172662360	NKX2-5	2488	100.00
5	172659112	172662360	NKX2-5	2488	100.00
5	174151536	174157896	MSX2	7392	100.00
5	174151536	174157896	MSX2	7392	100.00
5	176560026	176727216	NSD1	14234	99.95
5	176560026	176727216	NSD1	14234	99.95
5	176560026	176727216	NSD1	14234	99.95
5	177027101	177037348	B4GALT7	930	97.26
5	177419236	177423243	PROP1	9455	91.78
5	177576461	177580968	NHP2	14377	97.19
5	178405328	178423207	GRM6	4598	85.48
5	180028506	180076624	FLT4	3767	100.00
6	1610681	1614127	FOXC1	3800	98.92
6	1610681	1614127	FOXC1	3800	98.92
6	1610681	1614127	FOXC1	3800	98.92
6	3153903	3157760	TUBB2A	12412	99.48
6	3224495	3231964	TUBB2B	30829	100.00
6	10393419	10419892	TFAP2A	11742	99.77
6	12290596	12297427	EDN1	3176	100.00
6	12290596	12297427	EDN1	3176	100.00
6	24171984	24358280	DCDC2	18141	100.00
6	24495080	24537435	ALDH5A1	408	100.00
6	24775159	24786327	GMNN	17493	100.00
6	26104104	26104518	HIST1H4C	4787	100.00
6	26156559	26157343	HIST1H1E	4718	100.00
6	29640169	29648887	ZFP57	18791	99.81
6	30687978	30693203	TUBB	20778	93.93
6	30687978	30693203	TUBB	20778	93.93
6	31825436	31830683	NEU1	7758	96.15
6	31926857	31937532	SKIV2L	10898	100.00
6	32808494	32812480	PSMB8	9545	97.35
6	33130458	33160276	COL11A2	2187	99.29
6	33130458	33160276	COL11A2	2187	99.29
6	33130458	33160276	COL11A2	2187	99.29
6	33130458	33160276	COL11A2	2187	99.29
6	33130458	33160276	COL11A2	2187	99.29
6	33387847	33421466	SYNGAP1	11497	97.22

6	33387847	33421466	SYNGAP1	11497	97.22
6	35420138	35434880	FANCE	3586	88.76
6	36210980	36276372	PNPLA1	21246	97.26
6	39867354	39902290	MOCS1	7190	97.58
6	42931608	42946958	PEX6	8859	86.17
6	42931608	42946958	PEX6	8859	86.17
6	42952237	42980080	PPP2R5D	9312	100.00
6	43005355	43021683	CUL7	21024	100.00
6	43477440	43497323	POLR1C	20194	95.00
6	45295894	45632086	RUNX2	10472	100.00
6	49398073	49430904	MUT	7526	100.00
6	50786436	50815326	TFAP2B	11743	96.02
6	51480098	51952423	PKHD1	9016	100.00
6	57053607	57087078	RAB23	14263	100.00
6	70385694	70507003	LMBRD1	23038	80.94
6	70924764	71012786	COL9A1	2217	99.38
6	70924764	71012786	COL9A1	2217	99.38
6	73331520	73908574	KCNQ5	6299	100.00
6	73331520	73908574	KCNQ5	6299	100.00
6	74171301	74218959	MTO1	19261	78.07
6	74303102	74363878	SLC17A5	10933	100.00
6	74303102	74363878	SLC17A5	10933	100.00
6	80624529	80657297	ELOVL4	14415	99.37
6	80816364	81055987	BCKDHB	987	91.18
6	83870869	83903655	PGM3	8907	100.00
6	85397069	85474237	TBX18	11595	98.68
6	86215214	86303874	SNX14	14977	97.60
6	88180341	88222054	SLC35A1	11021	100.00
6	88224096	88299721	RARS2	21406	100.00
6	91223292	91296764	MAP3K7	6859	100.00
6	91223292	91296764	MAP3K7	6859	100.00
6	99316420	99395849	FBXL4	13601	100.00
6	101846664	102517958	GRIK2	4580	100.00
6	105175968	105307794	HACE1	21033	100.00
6	107018903	107077373	RTN4IP1	18647	96.51
6	107473761	107780768	PDSS2	23041	99.17
6	110012499	110146631	FIG4	16873	100.00
6	110012499	110146631	FIG4	16873	100.00
6	116440086	116479910	COL10A1	2185	100.00
6	117198375	117253326	RFX6	21478	97.70
6	117996665	118031803	NUS1	21042	45.35
6	121756838	121770873	GJA1	4274	100.00
6	121756838	121770873	GJA1	4274	100.00
6	121756838	121770873	GJA1	4274	100.00
6	121756838	121770873	GJA1	4274	100.00
6	129204342	129837714	LAMA2	6482	100.00
6	131894284	131905472	ARG1	663	100.00
6	132129156	132216295	ENPP1	3356	90.71
6	132129156	132216295	ENPP1	3356	90.71
6	135604670	135818914	AHI1	21575	97.22

6	137143717	137235075	PEX7	8860	99.08
6	137143717	137235075	PEX7	8860	99.08
6	137143717	137235075	PEX7	8860	99.08
6	142622991	142767403	ADGRG6	13841	100.00
6	143072604	143266338	HIVEP2	4921	100.00
6	143771944	143811147	PEX3	8858	100.00
6	143771944	143811147	PEX3	8858	100.00
6	146348782	146758734	GRM1	4593	99.08
6	149539777	149732749	TAB2	17075	99.81
6	151725989	151773259	RMND1	21176	100.00
6	152442819	152958936	SYNE1	17089	100.00
6	152442819	152958936	SYNE1	17089	100.00
6	157099063	157531913	ARID1B	18040	100.00
6	157099063	157531913	ARID1B	18040	100.00
6	158589384	158620376	GTF2H5	21157	96.76
6	159393903	159421219	RSPH3	21054	99.35
6	165740776	166400091	PDE10A	8772	94.66
6	167342992	167370679	RNASET2	21686	88.46
6	168841831	169073984	SMOC2	20323	99.05
7	192969	300711	FAM20C	22140	100.00
7	766338	829190	DNAAF5	26013	100.00
7	2552163	2568811	LFNG	6560	82.98
7	2577511	2595361	BRAT1	21701	93.49
7	5566782	5603415	ACTB	132	99.29
7	5566782	5603415	ACTB	132	99.29
7	6012870	6048756	PMS2	9122	78.34
7	6414154	6443608	RAC1	9801	93.40
7	16130817	16460947	ISPD	37276	94.99
7	19060614	19157295	TWIST1	12428	100.00
7	19060614	19157295	TWIST1	12428	100.00
7	22980878	23053749	FAM126A	24587	100.00
7	23145353	23217533	KLHL7	15646	100.00
7	27132612	27135615	HOXA1	5099	100.00
7	27132612	27135615	HOXA1	5099	100.00
7	27233122	27239725	HOXA13	5102	83.12
7	30050203	30066300	FKBP14	18625	100.00
7	33053742	33102409	NT5C3A	17820	67.60
7	33168856	33645680	BBS9	30000	100.00
7	33944523	34195484	BMPER	24154	99.27
7	35242042	35293758	TBX20	11598	100.00
7	39989636	40136733	CDK13	1733	91.36
7	40165622	40174258	MPLKIP	16002	100.00
7	42000548	42277469	GLI3	4319	100.00
7	42000548	42277469	GLI3	4319	100.00
7	42000548	42277469	GLI3	4319	100.00
7	42000548	42277469	GLI3	4319	100.00
7	44256749	44374176	CAMK2B	1461	100.00
7	47814250	47988088	PKD1L1	18053	96.74
7	50526134	50633154	DDC	2719	100.00
7	56078744	56119297	PSPH	9577	100.00

7	56078744	56119297	PSPH	9577	100.00
7	65425671	65447301	GUSB	4696	99.24
7	65540785	65558545	ASL	746	100.00
7	66093868	66276446	KCTD7	21957	88.60
7	66093868	66276446	KCTD7	21957	88.60
7	66452664	66460588	SBDS	19440	100.00
7	69063905	70258054	AUTS2	14262	98.02
7	73442119	73484237	ELN	3327	99.36
7	73442119	73484237	ELN	3327	99.36
7	75677369	75696826	MDH2	6971	100.00
7	75956116	75988348	YWHAG	12852	100.00
7	79763271	79848718	GNAI1	4384	88.54
7	91828283	91875480	KRIT1	1573	100.00
7	92116334	92157845	PEX1	8850	100.00
7	92116334	92157845	PEX1	8850	100.00
7	92116334	92157845	PEX1	8850	100.00
7	99699172	99707968	AP4M1	574	97.80
7	103112231	103629963	RELN	9957	99.51
7	106842000	107204959	COG5	14857	98.11
7	107531415	107572175	DLD	2898	100.00
7	107531415	107572175	DLD	2898	100.00
7	107564244	107643700	LAMB1	6486	99.67
7	113726382	114333827	FOXP2	13875	98.63
7	121715701	121784334	AASS	17366	100.00
7	121941448	121950745	FEZF1	22788	100.00
7	128828713	128853386	SMO	11119	94.29
7	130033612	130082274	CEP41	12370	96.43
7	137687070	137802732	AKR1D1	388	93.27
7	139476850	139720125	TBXAS1	11609	100.00
7	140419127	140624564	BRAF	1097	81.08
7	140419127	140624564	BRAF	1097	81.08
7	140419127	140624564	BRAF	1097	81.08
7	141250989	141355044	AGK	21869	98.19
7	145813453	148118090	CNTNAP2	13830	100.00
7	148504475	148581413	EZH2	3527	99.36
7	155592680	155604967	SHH	10848	100.00
7	155592680	155604967	SHH	10848	100.00
7	155592680	155604967	SHH	10848	100.00
7	155592680	155604967	SHH	10848	100.00
7	156786745	156803345	MNX1	4979	67.58
7	158649269	158749438	WDR60	21862	100.00
7	158649269	158749438	WDR60	21862	100.00
8	1703944	1734738	CLN8	2079	100.00
8	1703944	1734738	CLN8	2079	100.00
8	6264113	6501144	MCPH1	6954	99.62
8	11534468	11617511	GATA4	4173	79.08
8	15274724	15624158	TUSC3	30242	100.00
8	17913934	17942494	ASAH1	735	98.87
8	17913934	17942494	ASAH1	735	98.87
8	20054878	20084330	ATP6V1B2	854	98.63

8	21971928	21990897	HR	5172	89.43
8	21971928	21990897	HR	5172	89.43
8	27629466	27670157	ESCO2	27230	96.68
8	27629466	27670157	ESCO2	27230	96.68
8	30435835	30515768	GTF2E2	4651	100.00
8	33330904	33371119	TTI2	26262	100.00
8	37620111	37637283	PLPBP	9457	100.00
8	38001167	38008783	STAR	11359	100.00
8	38082736	38133076	DDHD2	29106	100.00
8	38268656	38326352	FGFR1	3688	100.00
8	38268656	38326352	FGFR1	3688	100.00
8	38268656	38326352	FGFR1	3688	100.00
8	38268656	38326352	FGFR1	3688	100.00
8	38268656	38326352	FGFR1	3688	100.00
8	38268656	38326352	FGFR1	3688	100.00
8	41786997	41909508	KAT6A	13013	100.00
8	42691817	42698468	THAP1	20856	100.00
8	42995556	43057998	HGSNAT	26527	93.82
8	55370495	55373448	SOX17	18122	100.00
8	57870492	57906403	IMPAD1	26019	100.00
8	61099906	61193971	CA8	1382	94.39
8	61591337	61779465	CHD7	20626	97.68
8	61591337	61779465	CHD7	20626	97.68
8	61591337	61779465	CHD7	20626	97.68
8	62413116	62627155	ASPH	757	100.00
8	67974661	68108498	CSPP1	26193	100.00
8	72109668	72274467	EYA1	3519	99.47
8	74884672	74895018	TMEM70	26050	97.83
8	77892494	77913280	PEX2	9717	100.00
8	77892494	77913280	PEX2	9717	100.00
8	77892494	77913280	PEX2	9717	100.00
8	86376081	86393722	CA2	1373	100.00
8	90945564	91015456	NBN	7652	96.50
8	92082424	92099323	OTUD6B	24281	100.00
8	94767072	94831462	TMEM67	28396	100.00
8	94767072	94831462	TMEM67	28396	100.00
8	94767072	94831462	TMEM67	28396	100.00
8	94767072	94831462	TMEM67	28396	100.00
8	96257147	96281429	C8orf37	27232	100.00
8	97154562	97173020	GDF6	4221	100.00
8	97154562	97173020	GDF6	4221	100.00
8	97238148	97247862	UQCRB	12582	100.00
8	97273943	97349223	PTDSS1	9587	100.00
8	100025494	100889808	VPS13B	2183	99.17
8	101170134	101271506	SPAG1	11212	96.44
8	102504660	102681954	GRHL2	2799	100.00
8	103216730	103251346	RRM2B	17296	100.00
8	104310661	104345094	FZD6	4044	100.00
8	116420724	116821899	TRPS1	12340	100.00
8	117858174	117887105	RAD21	9811	94.20

8	118806729	119124092	EXT1	3512	97.61
8	118806729	119124092	EXT1	3512	97.61
8	120007691	120118821	COLEC10	2220	100.00
8	133133108	133493200	KCNQ3	6297	100.00
8	133584320	133687838	LRRC6	16725	100.00
8	140742586	141468678	TRAPPC9	30832	99.19
8	144898514	144912029	PUF60	17042	96.85
8	145137493	145141119	GPAA1	4446	96.03
8	145149930	145152428	CYC1	2579	100.00
8	145736667	145743229	RECQL4	9949	100.00
8	145736667	145743229	RECQL4	9949	100.00
8	145736667	145743229	RECQL4	9949	100.00
9	214854	465259	DOCK8	19191	100.00
9	2015342	2193624	SMARCA2	11098	97.42
9	2015342	2193624	SMARCA2	11098	97.42
9	2621834	2660053	VLDLR	12698	100.00
9	3824127	4348392	GLIS3	28510	99.86
9	6532464	6645650	GLDC	4313	82.24
9	12685439	12710290	TYRP1	12450	100.00
9	14734664	14910993	FREM1	23399	98.27
9	26904081	26947461	PLAA	9043	100.00
9	27109139	27230173	TEK	11724	100.00
9	32972604	33025166	APTX	15984	91.35
9	34638130	34651032	GALT	4135	100.00
9	34650699	34661889	IL11RA	5967	100.00
9	34650699	34661889	IL11RA	5967	100.00
9	35073832	35080013	FANCG	3588	98.17
9	35088685	35096591	PIGO	23215	100.00
9	35657748	35658014	RMRP	10031	No protein
9	35681989	35691017	TPM2	12011	100.00
9	35736863	35749983	GBA2	18986	100.00
9	35792151	35809729	NPR2	7944	99.49
9	37766975	37801434	EXOSC3	17944	85.27
9	80037995	80263223	GNA14	4382	100.00
9	80331003	80646374	GNAQ	4390	70.61
9	80912059	80945009	PSAT1	19129	65.59
9	80912059	80945009	PSAT1	19129	65.59
9	87283466	87638505	NTRK2	8032	100.00
9	91933421	91974557	SECISBP2	30972	97.52
9	93976097	94124195	AUH	890	100.00
9	94325373	94712444	ROR2	10257	99.75
9	94325373	94712444	ROR2	10257	99.75
9	94325373	94712444	ROR2	10257	99.75
9	94972489	95056038	IARS	5330	100.00
9	95473645	95527094	BICD2	17208	98.68
9	97365415	97402531	FBP1	3606	100.00
9	97861336	98079991	FANCC	3584	100.00
9	98205262	98279339	PTCH1	9585	97.31
9	98205262	98279339	PTCH1	9585	97.31
9	98637983	98776842	ERCC6L2	26922	97.26

9	100437191	100459639	XPA	12814	95.99
9	100615536	100618986	FOXE1	3806	100.00
9	100819021	100845357	NANS	19237	100.00
9	101866320	101916474	TGFBR1	11772	93.58
9	101866320	101916474	TGFBR1	11772	93.58
9	101866320	101916474	TGFBR1	11772	93.58
9	101978708	101984238	ALG2	23159	100.00
9	104182860	104198105	ALDOB	417	96.18
9	108320411	108403399	FKTN	3622	88.31
9	108320411	108403399	FKTN	3622	88.31
9	108320411	108403399	FKTN	3622	88.31
9	108320411	108403399	FKTN	3622	88.31
9	109625378	109775915	ZNF462	21684	99.49
9	111892573	111929571	FRRS1L	1362	70.14
9	116148597	116163613	ALAD	395	100.00
9	119449581	119463579	TRIM32	16380	100.00
9	119449581	119463579	TRIM32	16380	100.00
9	123151147	123342448	CDK5RAP2	18672	100.00
9	126118449	126142603	CRB2	18688	97.94
9	127243516	127269709	NR5A1	7983	100.00
9	127243516	127269709	NR5A1	7983	100.00
9	129376722	129463311	LMX1B	6654	97.99
9	130374544	130457460	STXBP1	11444	100.00
9	130374544	130457460	STXBP1	11444	100.00
9	130965658	131017527	DNM1	2972	100.00
9	131084815	131096351	COQ4	19693	100.00
9	131102925	131123749	SLC27A4	10998	100.00
9	131266979	131304567	GLE1	4315	100.00
9	131314866	131395941	SPTAN1	11273	100.00
9	131395940	131419066	WDR34	28296	100.00
9	131395940	131419066	WDR34	28296	100.00
9	131445703	131458679	SET	10760	89.12
9	131707809	131709898	DOLK	23406	100.00
9	133320316	133376661	ASS1	758	88.30
9	133539981	133558368	PRDM12	13997	89.58
9	133589333	133763062	ABL1	76	100.00
9	133884469	133969860	LAMC3	6494	92.98
9	134378289	134399193	POMT1	9202	96.61
9	134378289	134399193	POMT1	9202	96.61
9	134378289	134399193	POMT1	9202	96.61
9	135766735	135820020	TSC1	12362	99.52
9	136218610	136223552	SURF1	11474	88.26
9	136218610	136223552	SURF1	11474	88.26
9	138594031	138684992	KCNT1	18865	98.41
9	138594031	138684992	KCNT1	18865	98.41
9	139088096	139096955	LHX3	6595	100.00
9	139323071	139334274	INPP5E	21474	99.38
9	139323071	139334274	INPP5E	21474	99.38
9	139388896	139440314	NOTCH1	7881	97.84
9	139388896	139440314	NOTCH1	7881	97.84

9	139981379	140003635	MAN1B1	6823	99.29
9	140032842	140063207	GRIN1	4584	100.00
9	140513444	140764468	EHMT1	24650	98.03
10	180405	300577	ZMYND11	16966	98.95
10	12110971	12165224	DHTKD1	23537	100.00
10	15555948	15762124	ITGA8	6144	100.00
10	23481256	23483181	PTF1A	23734	92.00
10	23481256	23483181	PTF1A	23734	92.00
10	26986588	27035727	PDSS1	17759	91.83
10	27280843	27389421	ANKRD26	29186	88.02
10	27793197	27831143	RAB18	14244	95.82
10	28064115	28287977	ARMC4	25583	100.00
10	28821422	28912041	WAC	17327	99.69
10	28821422	28912041	WAC	17327	99.69
10	43572475	43625799	RET	9967	99.22
10	43572475	43625799	RET	9967	99.22
10	50663414	50747584	ERCC6	3438	100.00
10	50663414	50747584	ERCC6	3438	100.00
10	50663414	50747584	ERCC6	3438	100.00
10	50663414	50747584	ERCC6	3438	100.00
10	64571756	64679660	EGR2	3239	100.00
10	69556427	69597924	DNAJC12	28908	100.00
10	70748487	70776738	KIF1BP	23419	100.00
10	71561644	71724031	COL13A1	2190	100.00
10	72192071	72207707	NODAL	7865	100.00
10	72642037	72648541	PCBD1	8646	99.05
10	73576055	73611126	PSAP	9498	100.00
10	73724123	73773322	CHST3	1971	99.86
10	74127098	74385899	MICU1	1530	94.20
10	76585340	76792380	KAT6B	17582	95.34
10	76585340	76792380	KAT6B	17582	95.34
10	79734907	79789303	POLR3A	30074	97.60
10	82031576	82049440	MAT1A	6903	96.80
10	88428206	88495825	LDB3	15710	100.00
10	88428206	88495825	LDB3	15710	100.00
10	88428206	88495825	LDB3	15710	100.00
10	88810243	88854623	GLUD1	4335	84.08
10	89419370	89507462	PAPSS2	8604	100.00
10	89622870	89731687	PTEN	9588	100.00
10	89622870	89731687	PTEN	9588	100.00
10	89622870	89731687	PTEN	9588	100.00
10	89622870	89731687	PTEN	9588	100.00
10	89622870	89731687	PTEN	9588	100.00
10	89622870	89731687	PTEN	9588	100.00
10	90521163	90537999	LIPN	23452	99.83
10	90694831	90751147	ACTA2	130	96.03
10	90694831	90751147	ACTA2	130	96.03
10	94353043	94415150	KIF11	6388	96.15
10	95753746	96092580	PLCE1	17175	98.37
10	97365696	97416463	ALDH18A1	9722	100.00

10	97365696	97416463	ALDH18A1	9722	100.00
10	97365696	97416463	ALDH18A1	9722	100.00
10	97423158	97453900	TCTN3	24519	100.00
10	100175955	100206684	HPS1	5163	100.00
10	100218875	100995619	HPSE2	18374	99.89
10	101471601	101491857	COX15	2263	97.57
10	101471601	101491857	COX15	2263	97.57
10	101948055	101989376	CHUK	1974	100.00
10	102495360	102589698	PAX2	8616	100.00
10	103989943	104001231	PITX3	9006	100.00
10	103989943	104001231	PITX3	9006	100.00
10	103989943	104001231	PITX3	9006	100.00
10	104263744	104393292	SUFU	16466	98.79
10	112327449	112364394	SMC3	2468	84.86
10	112679301	112773425	SHOC2	15454	99.37
10	122610687	122669036	WDR11	13831	92.63
10	123237848	123357972	FGFR2	3689	96.61
10	123237848	123357972	FGFR2	3689	96.61
10	123237848	123357972	FGFR2	3689	96.61
10	123237848	123357972	FGFR2	3689	96.61
10	123237848	123357972	FGFR2	3689	96.61
10	123237848	123357972	FGFR2	3689	96.61
10	123237848	123357972	FGFR2	3689	96.61
10	123237848	123357972	FGFR2	3689	96.61
10	127455022	127464390	MMP21	14357	97.95
10	127477146	127511817	UROS	12592	100.00
10	131633547	131762105	EBF3	19087	99.88
10	134598297	134599556	NKX6-2	19321	90.77
11	298200	299526	IFITM5	16644	94.49
11	532242	537287	HRAS	5173	100.00
11	532242	537287	HRAS	5173	100.00
11	644233	706715	DEAF1	14677	100.00
11	790475	798316	SLC25A22	19954	95.16
11	832843	839831	CD151	1630	100.00
11	1773982	1785222	CTSD	2529	100.00
11	2150342	2170833	IGF2	5466	100.00
11	2150342	2170833	IGF2	5466	100.00
11	2185159	2193107	TH	11782	95.49
11	2465914	2870339	KCNQ1	6294	94.73
11	2904443	2907111	CDKN1C	1786	82.86
11	3818954	3847601	PGAP2	17893	100.00
11	6411655	6416228	SMPD1	11120	100.00
11	6411655	6416228	SMPD1	11120	100.00
11	6634000	6640692	TPP1	2073	100.00
11	6642556	6677085	DCHS1	13681	98.79
11	9160372	9286937	DENND5A	19344	100.00
11	13513602	13517728	PTH	9606	97.41
11	13690217	13753893	FAR1	26222	94.12
11	17407406	17410878	KCNJ11	6257	100.00
11	17407406	17410878	KCNJ11	6257	100.00

11	17407406	17410878	KCNJ11	6257	100.00
11	17756359	17804602	KCNC1	6233	100.00
11	20620946	20680831	SLC6A5	11051	100.00
11	22214722	22304903	ANO5	27337	94.15
11	22214722	22304903	ANO5	27337	94.15
11	22644079	22647387	FANCF	3587	100.00
11	31806340	31839509	PAX6	8620	100.00
11	31806340	31839509	PAX6	8620	100.00
11	31806340	31839509	PAX6	8620	100.00
11	31806340	31839509	PAX6	8620	100.00
11	31806340	31839509	PAX6	8620	100.00
11	31806340	31839509	PAX6	8620	100.00
11	31806340	31839509	PAX6	8620	100.00
11	32409321	32457176	WT1	12796	99.29
11	32409321	32457176	WT1	12796	99.29
11	34937376	35042138	PDHX	21350	100.00
11	35272753	35441610	SLC1A2	10940	100.00
11	44117099	44266979	EXT2	3513	97.26
11	44281994	44331716	ALX4	450	100.00
11	44281994	44331716	ALX4	450	100.00
11	45825623	45834566	SLC35C1	20197	99.36
11	45931220	45940363	PEX16	8857	86.12
11	45931220	45940363	PEX16	8857	86.12
11	45950871	46142985	PHF21A	24156	100.00
11	46878419	46940193	LRP4	6696	100.00
11	47236493	47260767	DDB2	2718	96.08
11	47428683	47438047	SLC39A13	20859	100.00
11	47428683	47438047	SLC39A13	20859	100.00
11	47459308	47470730	RAPSN	9863	92.01
11	47459308	47470730	RAPSN	9863	92.01
11	57416465	57429340	CLP1	16999	100.00
11	57520715	57587018	CTNND1	2515	100.00
11	58910221	58922512	FAM111A	24725	98.47
11	61159159	61166335	TMEM216	25018	94.52
11	64373646	64490660	NRXN2	8009	100.00
11	65292548	65306175	SCYL1	14372	100.00
11	65306276	65326401	LTBP3	6716	100.00
11	65482367	65488418	RNASEH2C	24116	100.00
11	65769550	65771620	BANF1	17397	99.63
11	65837834	66012218	PACS1	30032	89.92
11	66278077	66301098	BBS1	966	100.00
11	66615704	66725847	PC	8636	100.00
11	67374323	67380006	NDUFV1	7716	96.77
11	67798084	67804111	NDUFS8	7715	99.53
11	67922330	67981295	KMT5B	24283	98.68
11	68080077	68216743	LRP5	6697	99.82
11	68080077	68216743	LRP5	6697	99.82
11	68080077	68216743	LRP5	6697	99.82
11	68080077	68216743	LRP5	6697	99.82
11	68080077	68216743	LRP5	6697	99.82

11	68671310	68708070	IGHMBP2	5542	96.32
11	69624992	69633792	FGF3	3681	100.00
11	70313961	70963623	SHANK2	14295	98.64
11	71139239	71163914	DHCR7	2860	100.00
11	71900602	71907345	FOLR1	3791	100.00
11	71934745	71950149	INPPL1	6080	100.00
11	72003469	72145692	CLPB	30664	100.00
11	73723763	73882255	C2CD3	24564	100.00
11	74202757	74204778	LIPT2	37216	92.24
11	76839310	76926284	MYO7A	7606	96.42
11	76839310	76926284	MYO7A	7606	96.42
11	77811982	77850706	ALG8	23161	100.00
11	85339629	85347580	TMEM126B	30883	94.08
11	85955586	85989855	EED	3188	94.27
11	88910620	89028927	TYR	12442	100.00
11	93517393	93547861	MED17	2375	100.00
11	94152895	94227074	MRE11	7230	83.28
11	95523129	95565857	CEP57	30794	93.08
11	101981192	102104154	YAP1	16262	88.43
11	102813724	102826463	MMP13	7159	100.00
11	102813724	102826463	MMP13	7159	100.00
11	102980160	103350591	DYNC2H1	2962	96.70
11	102980160	103350591	DYNC2H1	2962	96.70
11	107992243	108018503	ACAT1	93	98.99
11	108093211	108239829	ATM	795	96.35
11	108376158	108464465	EXPH5	30578	100.00
11	111652919	111742305	ALG9	15672	100.00
11	111895538	111935114	DLAT	2896	100.00
11	112097088	112140678	PTS	9689	92.95
11	118307205	118397539	KMT2A	7132	99.98
11	118443105	118473748	ARCN1	649	100.00
11	118967213	118979041	DPAGT1	2995	100.00
11	118967213	118979041	DPAGT1	2995	100.00
11	119076752	119178859	CBL	1541	99.34
11	119209652	119217383	MFRP	18121	100.00
11	119209652	119217383	MFRP	18121	100.00
11	121163162	121179403	SC5D	10547	97.89
11	122943035	123065989	CLMP	24039	99.02
11	125753509	125770543	HYLS1	26558	100.00
11	125825691	125933230	CDON	17104	98.95
11	126138950	126148026	FOXRED1	26927	99.93
11	130029457	130080271	ST14	11344	100.00
11	133938820	134021896	JAM3	15532	100.00
12	2079952	2802108	CACNA1C	1390	100.00
12	4382938	4414516	CCND2	1583	100.00
12	6679249	6716642	CHD4	1919	100.00
12	7052141	7055166	C12orf57	29521	98.43
12	7072408	7105520	EMG1	16912	100.00
12	7341281	7371170	PEX5	9719	100.00
12	7341281	7371170	PEX5	9719	100.00

12	7341281	7371170	PEX5	9719	100.00
12	13693165	14133053	GRIN2B	4586	98.58
12	13693165	14133053	GRIN2B	4586	98.58
12	13693165	14133053	GRIN2B	4586	98.58
12	14765576	14849519	GUCY2C	4688	100.00
12	14765576	14849519	GUCY2C	4688	100.00
12	15034115	15038860	MGP	7060	100.00
12	15125956	15134799	PDE6H	8790	95.63
12	15125956	15134799	PDE6H	8790	95.63
12	21590549	21623300	PYROXD1	26162	82.17
12	21950335	22094336	ABCC9	60	100.00
12	23682440	24103966	SOX5	11201	97.80
12	25357723	25403870	KRAS	6407	100.00
12	25357723	25403870	KRAS	6407	100.00
12	28111017	28125638	PTHLH	9607	100.00
12	28111017	28125638	PTHLH	9607	100.00
12	31226779	31257725	DDX11	2736	79.56
12	46123448	46301823	ARID2	18037	92.13
12	48235320	48336831	VDR	12679	99.02
12	48366748	48398269	COL2A1	2200	100.00
12	48366748	48398269	COL2A1	2200	100.00
12	48366748	48398269	COL2A1	2200	100.00
12	48366748	48398269	COL2A1	2200	100.00
12	48366748	48398269	COL2A1	2200	100.00
12	48366748	48398269	COL2A1	2200	100.00
12	48366748	48398269	COL2A1	2200	100.00
12	48366748	48398269	COL2A1	2200	100.00
12	49297893	49325623	CCDC65	29937	96.90
12	49359123	49365546	WNT10B	12775	100.00
12	49372398	49375459	WNT1	12774	100.00
12	49412758	49453557	KMT2D	7133	100.00
12	49578579	49583107	TUBA1A	20766	98.38
12	49578579	49583107	TUBA1A	20766	98.38
12	51984050	52206648	SCN8A	10596	99.72
12	51984050	52206648	SCN8A	10596	99.72
12	52959566	52967609	KRT74	28929	100.00
12	53701240	53718648	AAAS	13666	99.45
12	54332535	54340328	HOXC13	5125	100.00
12	56078352	56109827	ITGA7	6143	100.00
12	57403784	57422667	TAC3	11521	100.00
12	64173583	64203338	RXYLT1	13530	97.22
12	65107225	65153227	GNS	4422	93.85
12	65563351	65642107	LEMD3	28887	99.76
12	65563351	65642107	LEMD3	28887	99.76
12	69080514	69136785	NUP107	29914	99.95
12	76738254	76742222	BBS10	26291	99.31
12	80603233	80772870	OTOGL	26901	91.46
12	85673885	85695562	ALX1	1494	94.39
12	88442793	88535993	CEP290	29021	88.43
12	88442793	88535993	CEP290	29021	88.43

12	88442793	88535993	CEP290	29021	88.43
12	88442793	88535993	CEP290	29021	88.43
12	88442793	88535993	CEP290	29021	88.43
12	88536073	88593664	TMTC3	26899	96.25
12	89813495	89919801	POC1B	30836	96.90
12	94071151	94288616	CRADD	2340	91.00
12	94700225	94853764	CEP83	17966	98.27
12	102139275	102224716	GNPTAB	29670	100.00
12	102139275	102224716	GNPTAB	29670	100.00
12	102789645	102874423	IGF1	5464	100.00
12	103230663	103352188	PAH	8582	100.00
12	103230663	103352188	PAH	8582	100.00
12	106751436	106903976	POLR3B	30348	98.23
12	106751436	106903976	POLR3B	30348	98.23
12	109915207	109974507	UBE3B	13478	100.00
12	109991542	110011679	MMAB	19331	100.00
12	110220890	110271212	TRPV4	18083	100.00
12	110220890	110271212	TRPV4	18083	100.00
12	111051832	111087235	TCTN1	26113	97.04
12	112856155	112947717	PTPN11	9644	93.74
12	112856155	112947717	PTPN11	9644	93.74
12	114791736	114846247	TBX5	11604	100.00
12	115108059	115121969	TBX3	11602	98.97
12	116395711	116715143	MED13L	22962	97.91
12	120123595	120315095	CIT	1985	100.00
12	121163538	121177811	ACADS	90	100.00
12	122277433	122301502	HPD	5147	100.00
12	122277433	122301502	HPD	5147	100.00
12	123717463	123742506	C12orf65	26784	100.00
12	124155660	124192948	TCTN2	25774	100.00
13	20712394	20735188	GJA3	4277	100.00
13	20761609	20767037	GJB2	4284	100.00
13	20761609	20767037	GJB2	4284	100.00
13	20761609	20767037	GJB2	4284	100.00
13	20761609	20767037	GJB2	4284	100.00
13	20761609	20767037	GJB2	4284	100.00
13	22245522	22278637	FGF9	3687	100.00
13	23902965	24007841	SACS	10519	100.00
13	25457171	25497018	CENPJ	17272	100.00
13	25457171	25497018	CENPJ	17272	100.00
13	28194903	28241548	POLR1D	20422	100.00
13	32889611	32973805	BRCA2	1101	99.22
13	39261266	39460074	FREM2	25396	100.00
13	41363548	41384247	SLC25A15	10985	93.93
13	51483814	51544592	RNASEH2B	25671	100.00
13	52586534	52603800	ALG11	32456	100.00
13	77564795	77576652	CLN5	2076	100.00
13	78469616	78493903	EDNRB	3180	98.31
13	92000074	92006833	MIR17HG	23564	No protein
13	93879095	95059655	GPC6	4454	100.00

13	95226308	95248511	TGDS	20324	97.56
13	100634026	100639018	ZIC2	12873	94.43
13	100741269	101182686	PCCA	8653	95.38
13	101706130	102068843	NALCN	19082	100.00
13	101706130	102068843	NALCN	19082	100.00
13	101706130	102068843	NALCN	19082	100.00
13	103497194	103528345	ERCC5	3437	99.28
13	108859787	108870716	LIG4	6601	100.00
13	108859787	108870716	LIG4	6601	100.00
13	110801318	110959496	COL4A1	2202	95.48
13	110958159	111165374	COL4A2	2203	98.31
13	111293759	111365950	CARS2	25695	99.64
13	115079988	115092796	CHAMP1	20311	100.00
14	20914570	20923264	OSGEP	18028	98.43
14	21756098	21819460	RPGRIP1	13436	100.00
14	21756098	21819460	RPGRIP1	13436	100.00
14	21853353	21924285	CHD8	20153	99.68
14	23851199	23877486	MYH6	7576	97.46
14	23851199	23877486	MYH6	7576	97.46
14	23851199	23877486	MYH6	7576	97.46
14	24708849	24711880	TINF2	11824	100.00
14	29235050	29238870	FOXG1	3811	86.80
14	30045687	30661104	PRKD1	9407	100.00
14	31494312	31562818	AP4S1	575	100.00
14	31959162	32330430	NUBPL	20278	95.83
14	35179593	35184029	CFL2	1875	99.40
14	36985602	36990354	NKX2-1	11825	100.00
14	36985602	36990354	NKX2-1	11825	100.00
14	37126773	37148920	PAX9	8623	99.61
14	45605143	45670093	FANCM	23168	95.91
14	45605143	45670093	FANCM	23168	95.91
14	50087489	50090198	MGAT2	7045	100.00
14	50704281	50779266	L2HGDH	20499	96.84
14	51324609	51411454	PYGL	9725	100.00
14	53510686	53620000	DDHD1	19714	96.89
14	54416454	54425479	BMP4	1071	100.00
14	54416454	54425479	BMP4	1071	100.00
14	55308726	55369570	GCH1	4193	100.00
14	55308726	55369570	GCH1	4193	100.00
14	56955072	57117324	TMEM260	20185	97.69
14	57267425	57277197	OTX2	8522	98.66
14	58894103	59015216	KIAA0586	19960	95.67
14	61110133	61124977	SIX1	10887	97.66
14	61110133	61124977	SIX1	10887	97.66
14	68194091	68283307	ZFYVE26	20761	98.89
14	70320848	70499083	SMOC1	20318	97.25
14	74706175	74729441	VSX2	1975	99.72
14	74706175	74729441	VSX2	1975	99.72
14	74706175	74729441	VSX2	1975	99.72
14	74752126	74769759	ABCD4	68	98.60

14	74942895	74960880	NPC2	14537	100.00
14	74964873	75079306	LTBP2	6715	96.62
14	74964873	75079306	LTBP2	6715	96.62
14	76044960	76129557	FLVCR2	20105	100.00
14	76368479	76550928	IFT43	29669	100.00
14	76424442	76449334	TGFB3	11769	100.00
14	77741299	77787227	POMT2	19743	94.69
14	77741299	77787227	POMT2	19743	94.69
14	77741299	77787227	POMT2	19743	94.69
14	77893018	77924295	VIPAS39	20347	100.00
14	77972340	78083116	SPTLC2	11278	100.00
14	81421333	81612646	TSHR	12373	99.08
14	81421333	81612646	TSHR	12373	99.08
14	88304164	88460009	GALC	4115	97.23
14	89290497	89344335	TTC8	20087	100.00
14	89290497	89344335	TTC8	20087	100.00
14	91737667	91884188	CCDC88C	19967	98.76
14	92432335	92507240	TRIP11	12305	91.08
14	92788925	92962596	SLC24A4	10978	100.00
14	97263641	97398059	VRK1	12718	100.00
14	100704635	100749129	YY1	12856	100.00
14	102430865	102517129	DYNC1H1	2961	100.00
14	102430865	102517129	DYNC1H1	2961	100.00
14	102829300	102968818	TECPR2	19957	100.00
14	104029299	104073860	APOPT1	20492	100.00
14	105235686	105262088	AKT1	391	100.00
15	23888691	23891175	MAGEL2	6814	100.00
15	23888691	23891175	MAGEL2	6814	100.00
15	25582381	25684128	UBE3A	12496	77.33
15	26788693	27184686	GABRB3	4083	95.04
15	26788693	27184686	GABRB3	4083	95.04
15	31293264	31453476	TRPM1	7146	100.00
15	34525460	34630261	SLC12A6	10914	100.00
15	38544527	38649450	SPRED1	20249	97.53
15	40453224	40513337	BUB1B	1149	100.00
15	40697686	40728146	IVD	6186	100.00
15	40763160	40765353	CHST14	24464	98.05
15	40986972	41024354	RAD51	9817	88.17
15	41221538	41231237	DLL4	2910	98.20
15	43235095	43398311	UBR1	16808	98.10
15	43661419	43699293	TUBGCP4	16691	99.45
15	44854894	44955876	SPG11	11226	100.00
15	45653322	45694525	GATM	4175	100.00
15	45879321	45908197	BLOC1S6	8549	100.00
15	48700503	48938046	FBN1	3603	100.00
15	48700503	48938046	FBN1	3603	100.00
15	48700503	48938046	FBN1	3603	100.00
15	48700503	48938046	FBN1	3603	100.00
15	49005125	49103343	CEP152	29298	97.43
15	49005125	49103343	CEP152	29298	97.43

15	51200869	51298097	AP4E1	573	98.59
15	51633826	51700210	GLDN	29514	100.00
15	52413117	52483566	GNB5	4401	96.38
15	52599480	52821247	MYO5A	7602	98.62
15	52599480	52821247	MYO5A	7602	98.62
15	55702723	55800432	DNAAF4	21493	97.70
15	57210821	57591479	TCF12	11623	100.00
15	64679947	64747502	TRIP4	12310	100.00
15	65369154	65372276	KBTBD13	37227	100.00
15	66018392	66184329	RAB11A	9760	100.00
15	66679155	66784650	MAP2K1	6840	95.01
15	67356101	67487533	SMAD3	6769	98.83
15	68499330	68549549	CLN6	2077	99.15
15	68499330	68549549	CLN6	2077	99.15
15	72635775	72668817	HEXA	4878	99.94
15	72978527	73030817	BBS4	969	100.00
15	74471807	74504608	STRA6	30650	99.63
15	75182346	75191798	MPI	7216	100.00
15	75661720	75748183	SIN3A	19353	95.67
15	76507696	76603813	ETFA	3481	100.00
15	78396948	78423886	CIB2	24579	100.00
15	78396948	78423886	CIB2	24579	100.00
15	80444832	80479288	FAH	3579	100.00
15	83328033	83378666	AP3B2	567	98.86
15	85185999	85197574	WDR73	25928	100.00
15	89346674	89418585	ACAN	319	90.72
15	89346674	89418585	ACAN	319	90.72
15	89787180	89860492	FANCI	25568	100.00
15	89787180	89860492	FANCI	25568	100.00
15	89859534	89878092	POLG	9179	98.39
15	90152020	90198682	KIF7	30497	88.27
15	90152020	90198682	KIF7	30497	88.27
15	90303822	90321982	MESP2	29659	89.28
15	91260558	91358859	BLM	1058	100.00
15	91541646	91565833	VPS33B	12712	100.00
15	93426526	93571237	CHD2	1917	100.00
15	96869167	96883492	NR2F2	7976	100.00
15	99192200	99507759	IGF1R	5465	99.50
15	99192200	99507759	IGF1R	5465	99.50
15	101099574	101143435	LINS1	30922	99.47
15	101417919	101456831	ALDH1A3	409	98.93
15	101715928	101792137	CHSY1	17198	99.17
16	772582	776954	CCDC78	14153	100.00
16	1401924	1413352	GNPTG	23026	90.06
16	1494935	1525581	CLCN7	2025	97.52
16	1543345	1560458	TELO2	29099	100.00
16	1560428	1662111	IFT140	29077	100.00
16	1821891	1823156	MRPS34	16618	100.00
16	2097466	2138716	TSC2	12363	100.00
16	2097466	2138716	TSC2	12363	100.00

16	2525147	2555735	TBC1D24	29203	99.88
16	2525147	2555735	TBC1D24	29203	99.88
16	2525147	2555735	TBC1D24	29203	99.88
16	3074028	3077756	THOC6	28369	100.00
16	3631182	3661599	SLX4	23845	99.48
16	3775055	3930727	CREBBP	2348	93.50
16	3775055	3930727	CREBBP	2348	93.50
16	4364762	4389598	GLIS2	29450	100.00
16	4846969	4852951	ROGDI	29478	100.00
16	5083703	5137380	ALG1	18294	50.39
16	8882680	8943188	PMM2	9115	100.00
16	9852376	10276611	GRIN2A	4585	100.00
16	9852376	10276611	GRIN2A	4585	100.00
16	14014014	14046202	ERCC4	3436	100.00
16	14014014	14046202	ERCC4	3436	100.00
16	14014014	14046202	ERCC4	3436	100.00
16	14014014	14046202	ERCC4	3436	100.00
16	14529558	14726585	PARN	8609	100.00
16	15737124	15820210	NDE1	17619	100.00
16	16242785	16317379	ABCC6	57	91.78
16	17195626	17564738	XYLT1	15516	100.00
16	23399814	23464501	COG7	18622	100.00
16	23614488	23652631	PALB2	26144	100.00
16	28477983	28506896	CLN3	2074	100.00
16	28853732	28857729	TUFM	12420	99.42
16	29802040	29816706	KIF22	6391	100.00
16	29823177	29827201	PRRT2	30500	100.00
16	29823177	29827201	PRRT2	30500	100.00
16	30064411	30081778	ALDOA	414	90.31
16	30709530	30755602	SRCAP	16974	100.00
16	30968615	30996437	SETD1A	29010	100.00
16	30996519	31000473	HSD3B7	18324	96.58
16	31000577	31021949	STX1B	18539	100.00
16	46723555	46732306	ORC6	17151	100.00
16	51169886	51185278	SALL1	10524	99.57
16	53631595	53737850	RPGRIP1L	29168	100.00
16	53631595	53737850	RPGRIP1L	29168	100.00
16	53631595	53737850	RPGRIP1L	29168	100.00
16	54964774	54968397	IRX5	14361	100.00
16	56225302	56391356	GNAO1	4389	100.00
16	56500748	56554195	BBS2	967	100.00
16	57220049	57274387	RSPRY1	29420	100.00
16	57481337	57495187	COQ9	25302	99.59
16	57644564	57698944	ADGRG1	4512	100.00
16	58033450	58055522	USB1	25792	100.00
16	66541906	66586447	TK2	11831	95.86
16	67197288	67203848	HSF4	5227	100.00
16	67197288	67203848	HSF4	5227	100.00
16	67596310	67673086	CTCF	13723	98.40
16	68344877	68392466	PRMT7	25557	100.00

16	68670092	68756519	CDH3	1762	100.00
16	68670092	68756519	CDH3	1762	100.00
16	68771128	68869451	CDH1	1748	100.00
16	69354043	69373570	COG8	18623	99.56
16	70286198	70323446	AARS	20	99.46
16	70514471	70557468	COG4	18620	100.00
16	70841281	71264625	HYDIN	19368	99.74
16	71599563	71611033	TAT	11573	100.00
16	72042487	72058954	DHODH	2867	100.00
16	75661622	75682541	KARS	6215	100.00
16	75661622	75682541	KARS	6215	100.00
16	78133310	79246564	WWOX	12799	100.00
16	78133310	79246564	WWOX	12799	100.00
16	79619740	79634611	MAF	6776	85.64
16	79619740	79634611	MAF	6776	85.64
16	79619740	79634611	MAF	6776	85.64
16	83932731	83949787	MLYCD	7150	98.52
16	86544133	86548076	FOXF1	3809	100.00
16	86600857	86602539	FOXC2	3801	100.00
16	86600857	86602539	FOXC2	3801	100.00
16	87921625	87970135	CA5A	1377	95.64
16	88781751	88851619	PIEZO1	28993	97.71
16	88869621	88875666	CDT1	24576	98.29
16	88880142	88923378	GALNS	4122	100.00
16	89334038	89556969	ANKRD11	21316	95.51
16	89710839	89724253	CHMP1A	8740	100.00
16	89803957	89883065	FANCA	3582	100.00
16	89987800	90005169	TUBB3	20772	100.00
16	89987800	90005169	TUBB3	20772	100.00
16	90086037	90111383	GAS8	4166	99.63
17	1173853	1174754	BHLHA9	35126	66.81
17	1173853	1174754	BHLHA9	35126	66.81
17	1397865	1420182	INPP5K	33882	99.03
17	2496504	2588909	PAFAH1B1	8574	92.15
17	2496504	2588909	PAFAH1B1	8574	92.15
17	3375668	3406713	ASPA	756	89.28
17	3413796	3461289	TRPV3	18084	100.00
17	3539762	3564836	CTNS	2518	100.00
17	3539762	3564836	CTNS	2518	100.00
17	3539762	3564836	CTNS	2518	100.00
17	5336097	5352150	C1QBP	1243	76.98
17	6297013	6338519	AIPL1	359	100.00
17	6588032	6616886	SLC13A5	23089	100.00
17	7093209	7123021	DLG4	2903	100.00
17	7120444	7128592	ACADVL	92	99.71
17	7486847	7496107	MPDU1	7207	100.00
17	7589389	7606820	WRAP53	25522	100.00
17	8076772	8076905	SNORD118	32952	No protein
17	8130191	8151362	CTC1	26169	98.40
17	10293639	10325267	MYH8	7578	98.35

17	10293639	10325267	MYH8	7578	98.35
17	10531843	10560626	MYH3	7573	98.66
17	10531843	10560626	MYH3	7573	98.66
17	10583654	10601692	SCO1	10603	94.59
17	12895708	12921504	ELAC2	14198	100.00
17	13972813	14111994	COX10	2260	99.79
17	13972813	14111994	COX10	2260	99.79
17	15902694	15948329	TTC19	26006	100.00
17	16120505	16252115	PIGL	8966	100.00
17	16832849	16875432	TNFRSF13B	18153	100.00
17	17584787	17714767	RAI1	9834	100.00
17	19240867	19281495	B9D1	24123	100.00
17	19551449	19580911	ALDH3A2	403	99.75
17	26721661	26734215	SLC46A1	30521	98.99
17	26833261	26865914	FOXN1	12765	99.74
17	27052915	27070473	NEK8	13387	100.00
17	27052915	27070473	NEK8	13387	100.00
17	27573881	27581512	CRYBA1	2394	100.00
17	29295803	29326929	RNF135	21158	97.69
17	29421945	29709134	NF1	7765	81.99
17	29421945	29709134	NF1	7765	81.99
17	29421945	29709134	NF1	7765	81.99
17	29421945	29709134	NF1	7765	81.99
17	33901814	33905882	PEX12	8854	100.00
17	33901814	33905882	PEX12	8854	100.00
17	36046435	36105237	HNF1B	11630	96.06
17	36481413	36499730	GPR179	31371	99.93
17	36890150	36906070	PCGF2	12929	98.55
17	37827375	37853050	PGAP3	23719	100.00
17	38214543	38250120	THRA	11796	99.21
17	38443885	38459171	CDC6	1744	100.00
17	38781214	38804760	SMARCE1	11109	69.55
17	40086888	40117648	TTC25	25280	62.53
17	40351186	40428725	STAT5B	11367	98.98
17	40688190	40696467	NAGLU	7632	94.89
17	40713485	40718295	COASY	29932	100.00
17	40761694	40767252	TUBG1	12417	100.00
17	40834631	40851832	CNTNAP1	8011	100.00
17	41196312	41277500	BRCA1	1100	100.00
17	41717756	41739322	MEOX1	7013	100.00
17	42081914	42086431	NAGS	17996	100.00
17	42282401	42298994	UBTF	12511	100.00
17	42325753	42345509	SLC4A1	11027	99.63
17	42325753	42345509	SLC4A1	11027	99.63
17	42927311	42977030	EFTUD2	30858	100.00
17	42976510	42982758	CCDC103	32700	100.00
17	42982376	42994305	GFAP	4235	98.01
17	44107282	44302733	KANSL1	24565	97.81
17	44839872	44910520	WNT3	12782	99.72
17	46605888	46608359	HOXB1	5111	100.00

17	48133332	48167845	ITGA3	6139	100.00
17	48260650	48278993	COL1A1	2197	93.52
17	48260650	48278993	COL1A1	2197	93.52
17	48260650	48278993	COL1A1	2197	93.52
17	48260650	48278993	COL1A1	2197	93.52
17	48260650	48278993	COL1A1	2197	93.52
17	48260650	48278993	COL1A1	2197	93.52
17	48260650	48278993	COL1A1	2197	93.52
17	48260650	48278993	COL1A1	2197	93.52
17	48423453	48440499	XYLT2	15517	98.97
17	54671060	54672951	NOG	7866	100.00
17	54671060	54672951	NOG	7866	100.00
17	54671060	54672951	NOG	7866	100.00
17	54671060	54672951	NOG	7866	100.00
17	54671060	54672951	NOG	7866	100.00
17	56282803	56296966	MKS1	7121	97.98
17	56282803	56296966	MKS1	7121	97.98
17	56769934	56811703	RAD51C	9820	100.00
17	57059999	57184282	TRIM37	7523	100.00
17	57697219	57773671	CLTC	2092	100.00
17	58677544	58741849	PPM1D	9277	99.23
17	59529765	59562471	TBX4	11603	97.86
17	59758627	59940882	BRIP1	20473	98.47
17	61678231	61685725	TACO1	24316	89.93
17	62015914	62050278	SCN4A	10591	100.00
17	62015914	62050278	SCN4A	10591	100.00
17	62015914	62050278	SCN4A	10591	100.00
17	65821640	65980494	BPTF	3581	94.99
17	66507921	66547460	PRKAR1A	9388	68.95
17	66531254	66597530	FAM20A	23015	100.00
17	70117161	70122561	SOX9	11204	100.00
17	70117161	70122561	SOX9	11204	100.00
17	71189129	71204646	COG1	6545	100.00
17	73269073	73285591	SLC25A19	14409	92.90
17	73512141	73520820	TSEN54	27561	93.25
17	73747675	73761792	GALK1	4118	98.98
17	73937588	73975515	ACOX1	119	100.00
17	78010435	78074412	CCDC40	26090	100.00
17	78075355	78093678	GAA	4065	100.00
17	78109013	78120982	EIF4A3	18683	100.00
17	78180515	78194722	SGSH	10818	94.17
17	79476997	79490873	ACTG1	144	100.00
17	79617489	79630142	PDE6G	8789	100.00
17	79801035	79818570	P4HB	8548	100.00
17	79890260	79900288	PYCR1	9721	90.34
17	80709940	80900724	TBCD	11581	97.85
17	80787311	80798454	ZNF750	25843	100.00
18	2655737	2805015	SMCHD1	29090	97.03
18	3411606	3458409	TGIF1	11776	100.00
18	6941743	7117813	LAMA1	6481	97.80

18	6941743	7117813	LAMA1	6481	97.80
18	10666480	11148587	PIEZO2	26270	100.00
18	10666480	11148587	PIEZO2	26270	100.00
18	13882043	13915706	MC2R	6930	95.97
18	19749404	19782491	GATA6	4174	81.88
18	19749404	19782491	GATA6	4174	81.88
18	19749404	19782491	GATA6	4174	81.88
18	21086148	21166862	NPC1	7897	100.00
18	24034874	24237365	KCTD1	18249	100.00
18	28898052	28936992	DSG1	3048	97.59
18	31158579	31331156	ASXL3	29357	99.61
18	32556892	32723434	MAPRE2	6891	98.88
18	42260138	42648475	SETBP1	15573	97.03
18	42260138	42648475	SETBP1	15573	97.03
18	43427574	43547240	EPG5	29331	97.95
18	46570039	46987717	DYM	21317	100.00
18	46570039	46987717	DYM	21317	100.00
18	47349183	47721463	MYO5B	7603	93.01
18	48494410	48611415	SMAD4	6770	100.00
18	48494410	48611415	SMAD4	6770	100.00
18	48494410	48611415	SMAD4	6770	100.00
18	49866542	51057784	DCC	2701	100.00
18	52889562	53332018	TCF4	11634	99.96
18	55313658	55470333	ATP8B1	3706	99.25
18	56934267	56941318	RAX	18662	100.00
18	57098172	57364612	CCBE1	29426	100.00
18	59710800	59854351	PIGN	8967	99.31
18	67671029	67873181	RTTN	18654	100.00
18	77439801	77514510	CTDP1	2498	99.48
18	77732867	77793949	TXNL4A	30551	100.00
19	1103936	1106787	GPX4	4556	85.86
19	1383526	1395583	NDUFS7	7714	98.91
19	1397091	1401569	GAMT	4136	99.14
19	3094408	3124002	GNA11	4379	100.00
19	4090319	4124126	MAP2K2	6842	96.59
19	5691845	5720583	LONP1	9479	100.00
19	6361463	6368919	CLPP	2084	95.92
19	6494330	6502859	TUBB4A	20774	100.00
19	7587512	7598895	MCOLN1	13356	99.52
19	7694623	7696842	PET100	40038	100.00
19	8454865	8469318	RAB11B	9761	100.00
19	11071598	11176071	SMARCA4	11100	100.00
19	11071598	11176071	SMARCA4	11100	100.00
19	11309971	11373157	DOCK6	19189	100.00
19	11531272	11546603	CCDC151	28303	99.44
19	11685475	11689823	ACP5	124	97.24
19	12757325	12777556	MAN2B1	6826	96.48
19	12917394	12924452	RNASEH2A	18518	100.00
19	12995237	12997995	KLF1	6345	100.00
19	13001840	13025021	GCDH	4189	99.58

19	13106422	13209610	NFIX	7788	100.00
19	13106422	13209610	NFIX	7788	100.00
19	13228917	13251955	NACC1	20967	100.00
19	14017014	14041692	CC2D1A	30237	100.00
19	17003758	17137625	CPAMD8	23228	97.55
19	17445729	17453544	GTPBP3	14880	100.00
19	17935589	17958880	JAK3	6193	92.16
19	17982782	18005983	SLC5A5	11040	99.90
19	18263928	18281350	PIK3R2	8980	99.17
19	18893583	18902123	COMP	2227	92.39
19	18893583	18902123	COMP	2227	92.39
19	33877856	34012700	PEPD	8840	98.88
19	35521588	35531352	SCN1B	10586	96.90
19	35521588	35531352	SCN1B	10586	96.90
19	35615417	35633355	LGI4	18712	96.63
19	36139125	36149763	COX6B1	2280	100.00
19	36208921	36229779	KMT2B	15840	92.62
19	36316866	36360189	NPHS1	7908	100.00
19	36486090	36487220	SDHAF1	33867	100.00
19	36545783	36596008	WDR62	24502	100.00
19	38924339	39078204	RYR1	10483	100.00
19	39989535	39999121	DLL3	2909	92.14
19	41807492	41859816	TGFB1	11766	100.00
19	41884215	41930910	BCKDHA	986	99.40
19	42363988	42376994	RPS19	10402	100.00
19	42470734	42501649	ATP1A3	801	100.00
19	42470734	42501649	ATP1A3	801	100.00
19	42751724	42759309	ERF	3444	100.00
19	42751724	42759309	ERF	3444	100.00
19	42829761	42882921	MEGF8	3233	98.88
19	44010871	44031396	ETHE1	23287	100.00
19	44235301	44259142	SMG9	25763	100.00
19	45853095	45874176	ERCC2	3434	100.00
19	45853095	45874176	ERCC2	3434	100.00
19	45853095	45874176	ERCC2	3434	100.00
19	45910591	45982086	ERCC1	3433	100.00
19	45910591	45982086	ERCC1	3433	100.00
19	46268043	46272484	SIX5	10891	100.00
19	46272975	46285810	DMPK	2933	100.00
19	46913629	46916841	CCDC8	25367	100.00
19	47249303	47280245	FKRP	17997	100.00
19	47249303	47280245	FKRP	17997	100.00
19	47249303	47280245	FKRP	17997	100.00
19	47978401	47987525	KPTN	6404	100.00
19	48322703	48346587	CRX	2383	100.00
19	48799714	48825151	CCDC114	26560	100.00
19	48898132	48948188	GRIN2D	4588	64.82
19	49468558	49470135	FTL	3999	93.37
19	50138549	50143458	RRAS	10447	98.78
19	50364461	50371166	PNKP	9154	100.00

19	50364461	50371166	PNKP	9154	100.00
19	50410082	50433020	NUP62	8066	100.00
19	50815194	50836772	KCNC3	6235	73.92
19	50887461	50921273	POLD1	9175	78.87
19	51165084	51222707	SHANK1	15474	100.00
19	51848423	51869672	ETFB	3482	100.00
19	52693292	52730687	PPP2R1A	9302	99.27
19	54641444	54659419	CNOT3	7879	100.00
19	54677107	54693733	MBOAT7	15505	98.17
19	54693789	54697585	TSEN34	15506	89.82
19	55670031	55678090	DNAAF3	30492	100.00
20	416124	443197	TBC1D20	16133	99.91
20	459116	524465	CSNK2A1	2457	99.83
20	740724	749131	SLC52A3	16187	100.00
20	939095	982907	RSPO4	16175	100.00
20	2442280	2451499	SNRPB	11153	97.93
20	3208063	3219836	SLC4A11	16438	100.00
20	6748311	6760927	BMP2	1069	100.00
20	8112824	8949003	PLCB1	15917	100.00
20	9049410	9461889	PLCB4	9059	100.00
20	10199478	10288066	SNAP25	11132	100.00
20	10381657	10414870	MKKS	7108	100.00
20	10381657	10414870	MKKS	7108	100.00
20	10618332	10654694	JAG1	6188	97.82
20	18488137	18542059	SEC23B	10702	100.00
20	19867165	19983101	RIN2	18750	99.49
20	23342787	23353700	GZF1	15808	99.81
20	30946155	31027122	ASXL1	18318	99.01
20	31350191	31397162	DNMT3B	2979	100.00
20	32951041	33099198	ITCH	13890	93.16
20	34021145	34042568	GDF5	4220	100.00
20	34021145	34042568	GDF5	4220	100.00
20	34021145	34042568	GDF5	4220	100.00
20	34021145	34042568	GDF5	4220	100.00
20	34021145	34042568	GDF5	4220	100.00
20	34021145	34042568	GDF5	4220	100.00
20	34021145	34042568	GDF5	4220	100.00
20	34021145	34042568	GDF5	4220	100.00
20	35518632	35580246	SAMHD1	15925	100.00
20	39314488	39317880	MAFB	6408	100.00
20	39314488	39317880	MAFB	6408	100.00
20	42984340	43061485	HNF4A	5024	97.47
20	42984340	43061485	HNF4A	5024	97.47
20	43248163	43280874	ADA	186	100.00
20	44044717	44054884	PIGT	14938	100.00
20	44518783	44527459	CTSA	9251	100.00
20	44994688	45061704	ELMO2	17233	100.00
20	45338126	45364965	SLC2A10	13444	100.00
20	47538427	47653230	ARFGEF2	15853	100.00
20	47980414	48099184	KCNB1	6231	99.57

20	49505585	49547958	ADNP	15766	100.00
20	49551404	49575092	DPM1	3005	96.17
20	50400581	50419059	SALL4	15924	97.41
20	50400581	50419059	SALL4	15924	97.41
20	57414773	57486247	GNAS	4392	100.00
20	57414773	57486247	GNAS	4392	100.00
20	57414773	57486247	GNAS	4392	100.00
20	57414773	57486247	GNAS	4392	100.00
20	61447596	61472511	COL9A3	2219	97.68
20	61975420	62009753	CHRNA4	1958	94.61
20	62037542	62103993	KCNQ2	6296	100.00
20	62037542	62103993	KCNQ2	6296	100.00
20	62119366	62130505	EEF1A2	3192	100.00
20	62289163	62328416	RTEL1	15888	85.21
20	62289163	62328416	RTEL1	15888	85.21
21	33964389	33985176	CFAP298	1301	100.00
21	34914924	34949812	SON	11183	94.93
21	35818988	35884573	KCNE1	6240	100.00
21	38123189	38362536	HLCS	4976	100.00
21	38738092	38889753	DYRK1A	3091	100.00
21	38979678	39288749	KCNJ6	6267	100.00
21	43159529	43187266	RIPK4	496	100.00
21	43892596	43916464	RSPH1	12371	100.00
21	44473301	44497053	CBS	1550	92.24
21	44589118	44592915	CRYAA	2388	100.00
21	44589118	44592915	CRYAA	2388	100.00
21	44834395	44847008	SIK1	11142	100.00
21	45192393	45196326	CSTB	2482	100.00
21	45705721	45718531	AIRE	360	100.00
21	45748827	45759285	C21orf2	1260	98.14
21	46825052	46933634	COL18A1	2195	84.25
21	47401651	47424964	COL6A1	2211	100.00
21	47556176	47575481	FTCD	3974	100.00
21	47744036	47865682	PCNT	16068	96.44
22	18560689	18613905	PEX26	22965	100.00
22	18560689	18613905	PEX26	22965	100.00
22	18560689	18613905	PEX26	22965	100.00
22	18560689	18613905	PEX26	22965	100.00
22	18593097	18629321	TUBA8	12410	99.93
22	18632666	18660164	USP18	12616	95.89
22	19466982	19508135	CDC45	1739	94.64
22	19744226	19771116	TBX1	11592	82.53
22	20004537	20053449	TANGO2	25439	100.00
22	20778874	20792146	SCARF2	19869	98.42
22	21213271	21245506	SNAP29	11133	100.00
22	24129150	24176703	SMARCB1	11103	100.00
22	24129150	24176703	SMARCB1	11103	100.00
22	24666786	24813708	SPECC1L	29022	99.52
22	25595817	25603330	CRYBB3	2400	100.00
22	25615489	25627836	CRYBB2	2398	100.00

	22	25615489	25627836	CRYBB2	2398	100.00
	22	26995242	27014052	CRYBB1	2397	98.55
	22	26995242	27014052	CRYBB1	2397	98.55
	22	27017928	27026636	CRYBA4	2396	100.00
	22	31002825	31023265	TCN2	11653	100.00
	22	32149944	32303012	DEPDC5	18423	100.00
	22	33558212	34318829	LARGE1	6511	98.77
	22	33558212	34318829	LARGE1	6511	98.77
	22	36677327	36784063	MYH9	7579	99.42
	22	36677327	36784063	MYH9	7579	99.42
	22	36677327	36784063	MYH9	7579	99.42
	22	36677327	36784063	MYH9	7579	99.42
	22	36677327	36784063	MYH9	7579	99.42
	22	36677327	36784063	MYH9	7579	99.42
	22	37461476	37505603	TMPRSS6	16517	100.00
	22	38366693	38383429	SOX10	11190	100.00
	22	38366693	38383429	SOX10	11190	100.00
	22	38366693	38383429	SOX10	11190	100.00
	22	38366693	38383429	SOX10	11190	100.00
	22	38366693	38383429	SOX10	11190	100.00
	22	38507502	38601697	PLA2G6	9039	98.64
	22	38507502	38601697	PLA2G6	9039	98.64
	22	40742507	40786467	ADSL	291	100.00
	22	41487790	41576081	EP300	3373	98.30
	22	41865129	41924993	ACO2	118	87.75
	22	42454358	42466846	NAGA	7631	100.00
	22	42454358	42466846	NAGA	7631	100.00
	22	42556019	42739622	TCF20	11631	100.00
	22	43013846	43045574	CYB5R3	2873	100.00
	22	50293877	50312106	ALG12	19358	100.00
	22	50497820	50524331	MLC1	17082	100.00
	22	50656118	50683421	TUBGCP6	18127	100.00
	22	50961997	50964868	SCO2	10604	100.00
	22	51061182	51066607	ARSA	713	100.00
	22	51112843	51171726	SHANK3	14294	93.31
M		15956	16023	MT-TP	7494	No protein
X		585079	620146	SHOX	10853	91.35
X		585079	620146	SHOX	10853	91.35
X		2852699	2886286	ARSE	719	99.18
X		7137497	7272851	STS	11425	94.37
X		10413350	10851773	MID1	7095	99.74
X		11129421	11141198	HCCS	4837	94.67
X		11776278	11793870	MSL3	7370	76.88
X		12156585	12742642	FRMPD4	29007	98.37
X		13730363	13752754	TRAPPC2	23068	68.76
X		13752832	13787480	OFD1	2567	52.67
X		13752832	13787480	OFD1	2567	52.67
X		13752832	13787480	OFD1	2567	52.67
X		14861529	14891191	FANCB	3583	96.86
X		15337573	15353676	PIGA	8957	85.77

X	15843929	15873054	AP1S2	560	68.35
X	17393543	17754114	NHS	7820	94.60
X	17393543	17754114	NHS	7820	94.60
X	18443703	18671749	CDKL5	11411	99.74
X	19362011	19379823	PDHA1	8806	95.12
X	19362011	19379823	PDHA1	8806	95.12
X	19362011	19379823	PDHA1	8806	95.12
X	20168029	20285523	RPS6KA3	10432	97.26
X	20168029	20285523	RPS6KA3	10432	97.26
X	21392536	21672813	CNKSR2	19701	92.32
X	21958691	22025798	SMS	11123	67.85
X	23352133	23422489	PTCHD1	26392	100.00
X	24072833	24096088	EIF2S3	3267	86.08
X	25021811	25034065	ARX	18060	100.00
X	25021811	25034065	ARX	18060	100.00
X	25021811	25034065	ARX	18060	100.00
X	25021811	25034065	ARX	18060	100.00
X	25021811	25034065	ARX	18060	100.00
X	28605516	29974840	IL1RAPL1	5996	97.00
X	30671476	30748725	GK	4289	52.83
X	38211798	38280703	OTC	8512	100.00
X	38420623	38548169	TSPAN7	11854	100.00
X	39909068	40036582	BCOR	20893	97.85
X	40944888	41095832	USP9X	12632	96.39
X	40944888	41095832	USP9X	12632	96.39
X	41192651	41223725	DDX3X	2745	96.22
X	41192651	41223725	DDX3X	2745	96.22
X	41306687	41334963	NYX	8082	96.40
X	41374187	41782716	CASK	1497	95.02
X	41374187	41782716	CASK	1497	95.02
X	41374187	41782716	CASK	1497	95.02
X	43515467	43606068	MAOA	6833	100.00
X	43808022	43832750	NDP	7678	100.00
X	44732757	44971847	KDM6A	12637	79.25
X	47001615	47004903	NDUFB11	20372	92.68
X	47004268	47046212	RBM10	9896	98.50
X	47431303	47479252	SYN1	11494	100.00
X	48334541	48344752	FTSJ1	13254	94.04
X	48367350	48379202	PORCN	17652	100.00
X	48379546	48387104	EBP	3133	94.37
X	48755195	48760420	PQBP1	9330	100.00
X	48760459	48769235	SLC35A2	11022	96.31
X	48929385	48958108	WDR45	28912	91.16
X	49044269	49056718	SYP	11506	99.79
X	49091927	49106987	CCDC22	28909	96.13
X	49106897	49121288	FOXP3	6106	95.37
X	49644470	49647166	USP27X	13486	100.00
X	51486481	51489324	GSPT2	4622	100.00
X	53220503	53254604	KDM5C	11114	98.48
X	53262058	53350522	IQSEC2	29059	95.07

X	53401070	53449677	SMC1A	11111	99.84
X	53458206	53461320	HSD17B10	4800	98.09
X	53458206	53461320	HSD17B10	4800	98.09
X	53559057	53713673	HUWE1	30892	96.42
X	53963109	54075391	PHF8	20672	97.36
X	54471887	54522599	FGD1	3663	95.42
X	63404997	63425624	AMER1	26837	99.65
X	64136250	64254593	ZC4H2	24931	100.00
X	64136250	64254593	ZC4H2	24931	100.00
X	66764465	66950461	AR	644	99.70
X	66764465	66950461	AR	644	99.70
X	67262186	67653755	OPHN1	8148	99.41
X	68048840	68061990	EFNB1	3226	100.00
X	68835911	69259319	EDA	3157	100.00
X	68835911	69259319	EDA	3157	100.00
X	69664711	69725337	DLG3	2902	100.00
X	70338406	70362303	MED12	11957	94.89
X	70338406	70362303	MED12	11957	94.89
X	70503042	70521018	NONO	7871	95.48
X	70586114	70752224	TAF1	11535	95.68
X	71549366	71792953	HDAC8	13315	100.00
X	71549366	71792953	HDAC8	13315	100.00
X	71549366	71792953	HDAC8	13315	100.00
X	73641085	73753752	SLC16A2	10923	92.96
X	73805052	73834452	RLIM	13429	100.00
X	73952684	74145282	NEXMIF	29433	100.00
X	73952684	74145282	NEXMIF	29433	100.00
X	74273115	74376567	ABCB7	48	96.93
X	76760356	77041702	ATRX	886	95.55
X	76760356	77041702	ATRX	886	95.55
X	77154935	77162870	COX7B	2291	16.46
X	77166194	77305892	ATP7A	869	97.60
X	77166194	77305892	ATP7A	869	97.60
X	77166194	77305892	ATP7A	869	97.60
X	77320685	77384793	PGK1	8896	70.65
X	79270255	79287268	TBX22	11600	95.84
X	79926353	80065187	BRWD3	17342	91.36
X	84498997	84528368	ZNF711	13128	98.73
X	99546642	99665271	PCDH19	14270	99.62
X	99899215	99926296	SRPX2	30668	97.44
X	99899215	99926296	SRPX2	30668	97.44
X	100600649	100604184	TIMM8A	11817	76.87
X	100600649	100604184	TIMM8A	11817	76.87
X	100663283	100669121	HNRNPH2	5042	100.00
X	103028647	103047548	PLP1	9086	99.28
X	103028647	103047548	PLP1	9086	99.28
X	106871737	106894256	PRPS1	9462	100.00
X	106871737	106894256	PRPS1	9462	100.00
X	106871737	106894256	PRPS1	9462	100.00
X	106871737	106894256	PRPS1	9462	100.00

X	108867473	108976632	ACSL4	3571	89.50
X	108867473	108976632	ACSL4	3571	89.50
X	109917084	110039286	CHRDL1	29861	100.00
X	110187513	110470589	PAK3	8592	76.84
X	110187513	110470589	PAK3	8592	76.84
X	110537007	110655603	DCX	2714	100.00
X	110537007	110655603	DCX	2714	100.00
X	110909043	111003877	ALG13	30881	93.00
X	110909043	111003877	ALG13	30881	93.00
X	110909043	111003877	ALG13	30881	93.00
X	110909043	111003877	ALG13	30881	93.00
X	118708501	118718381	UBE2A	12472	96.08
X	118967985	118986961	UPF3B	20439	93.38
X	119005450	119010625	NDUFA1	7683	100.00
X	119561682	119603220	LAMP2	6501	88.83
X	119658464	119709649	CUL4B	2555	86.33
X	122318006	122624766	GRIA3	4573	93.01
X	122734412	122866906	THOC2	19073	90.72
X	128673826	128726538	OCRL	8108	100.00
X	128673826	128726538	OCRL	8108	100.00
X	128937264	128977885	ZDHHC9	18475	96.57
X	129263337	129299861	AIFM1	8768	100.00
X	129263337	129299861	AIFM1	8768	100.00
X	130407480	130533677	IGSF1	5948	98.24
X	131211021	131262048	FRMD7	8079	100.00
X	132669773	133119922	GPC3	4451	94.92
X	133507283	133562820	PHF6	18145	78.97
X	133594183	133654543	HPRT1	5157	93.15
X	133594183	133654543	HPRT1	5157	93.15
X	135067598	135129423	SLC9A6	11079	93.40
X	135229559	135293518	FHL1	3702	95.68
X	136648301	136659850	ZIC3	12874	100.00
X	136648301	136659850	ZIC3	12874	100.00
X	139585152	139587225	SOX3	11199	96.79
X	139585152	139587225	SOX3	11199	96.79
X	146993469	147032645	FMR1	3775	94.58
X	146993469	147032645	FMR1	3775	94.58
X	146993469	147032645	FMR1	3775	94.58
X	147582139	148082193	AFF2	3776	99.29
X	148558521	148615470	IDS	5389	98.97
X	149529689	149682448	MAMLD1	2568	100.00
X	149737069	149841795	MTM1	7448	89.74
X	151999511	152038273	NSDHL	13398	100.00
X	151999511	152038273	NSDHL	13398	100.00
X	152760397	152775012	BGN	1044	100.00
X	152760397	152775012	BGN	1044	100.00
X	152853377	152865500	CCNQ	28434	77.19
X	152953554	152962048	SLC6A8	11055	85.95
X	152965947	152990152	BCAP31	16695	83.86
X	152990323	153010216	ABCD1	61	73.01

X	153126969	153174677	L1CAM	6470	99.13
X	153126969	153174677	L1CAM	6470	99.13
X	153126969	153174677	L1CAM	6470	99.13
X	153126969	153174677	L1CAM	6470	99.13
X	153194695	153200676	NAA10	18704	100.00
X	153194695	153200676	NAA10	18704	100.00
X	153194695	153200676	NAA10	18704	100.00
X	153194695	153200676	NAA10	18704	100.00
X	153213004	153237258	HCFC1	4839	96.76
X	153213004	153237258	HCFC1	4839	96.76
X	153287024	153363212	MECP2	6990	100.00
X	153287024	153363212	MECP2	6990	100.00
X	153287024	153363212	MECP2	6990	100.00
X	153287024	153363212	MECP2	6990	100.00
X	153287024	153363212	MECP2	6990	100.00
X	153576892	153603006	FLNA	3754	100.00
X	153576892	153603006	FLNA	3754	100.00
X	153576892	153603006	FLNA	3754	100.00
X	153576892	153603006	FLNA	3754	100.00
X	153576892	153603006	FLNA	3754	100.00
X	153576892	153603006	FLNA	3754	100.00
X	153576892	153603006	FLNA	3754	100.00
X	153576892	153603006	FLNA	3754	100.00
X	153576892	153603006	FLNA	3754	100.00
X	153639854	153650065	TAZ	11577	100.00
X	153665266	153671814	GDI1	4226	100.00
X	153665266	153671814	GDI1	4226	100.00
X	153769414	153796782	IKBKG	5961	78.89
X	153769414	153796782	IKBKG	5961	78.89
X	153769414	153796782	IKBKG	5961	78.89
X	153769414	153796782	IKBKG	5961	78.89
X	153769414	153796782	IKBKG	5961	78.89
X	153991031	154005964	DKC1	2890	100.00
X	153991031	154005964	DKC1	2890	100.00
X	154487526	154493874	RAB39B	16499	100.00
Y	2654896	2655740	SRY	11311	94.96

Supplementary Figure 1: Organisational chart illustrating the role of the clinical review panel in the assessment of genetic variants detected in the PAGE study

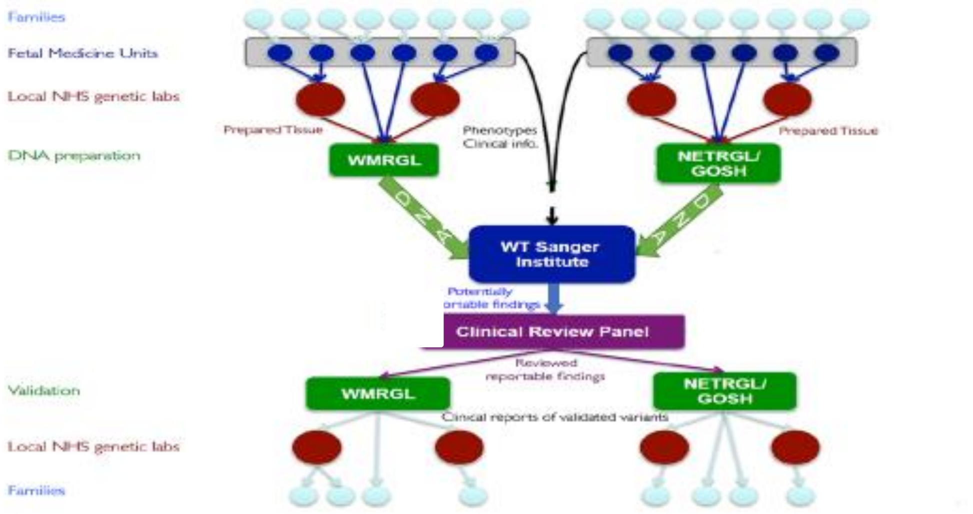

Supplementary Figure 2: Simplified (omitting quality control steps) overview of the bioinformatics pipeline.

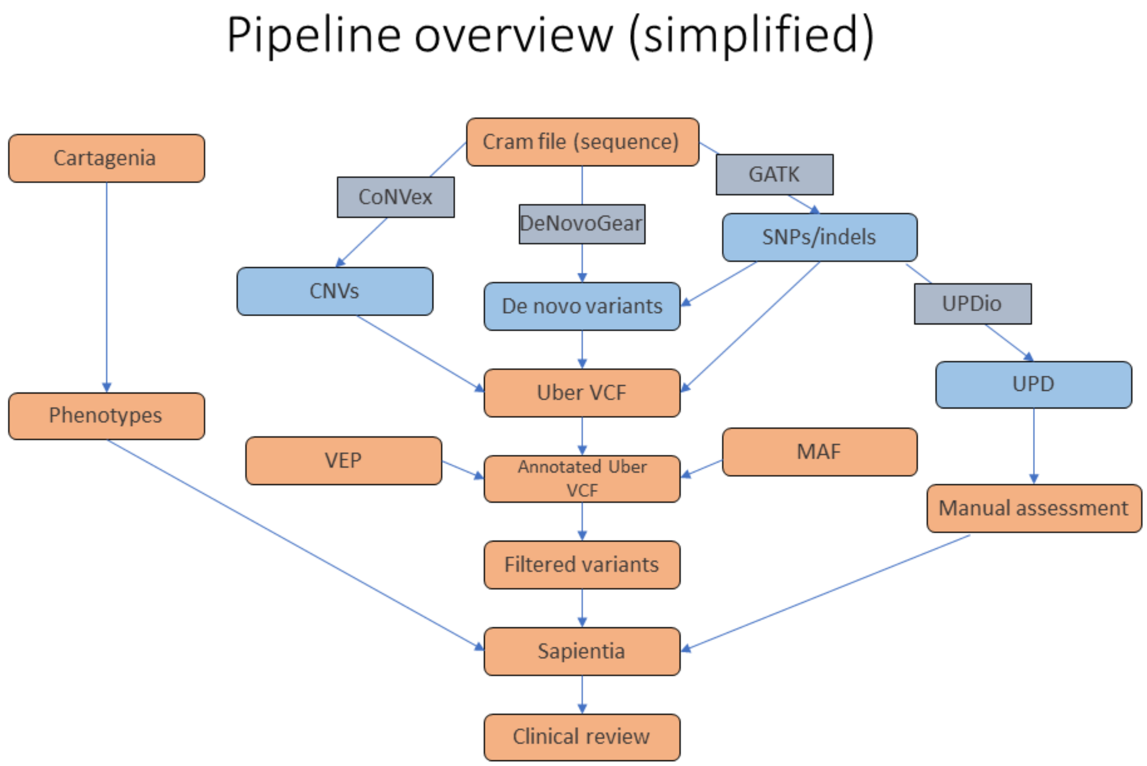

Abbreviations:
CoNVex = copy number variation estimation in exome sequencing data
CNV = copy number variant
GATK = genome analysis toolkit (https://software.broadinstitute.org/gatk/)
MAF = minor allele frequency
UPD = uniparental disomy
UPDio = detecting uniparental disomy from trio genotypes
VEP = variant effect predictor (www.ensembl.org/vep)
VCF = variant call format

Case Study 1 (PP1579/ EGAN00001402048)

Clinical Background: An ultrasound scan at 20 weeks gestation revealed dysgenesis of the corpus callosum and lissencephaly. Quantitative Fluorescence (QF)-PCR to detect common aneuploidies and chromosomal microarray (CMA) did not reveal a pathogenic anomaly and the pregnancy was terminated at 20 weeks gestation.

Whole exome sequencing (WES) variant data: WES revealed an apparently *de novo* heterozygous NM_178014.2:c.860C>T, p.(Pro287Leu) missense variant in the *TUBB* (formerly *TUBB5*) gene (MIM: 191330) in a highly conserved nucleotide (phyloP: 4.97 [-14.1;6.4]) and moderately conserved amino acid residue (considering 12 species) within the highly conserved C-terminus domain of the beta-tubulin protein. *In silico* tools predict a deleterious effect on protein function (SIFT: Deleterious, MutationTaster: disease causing, PolyPhen v2.0: possibly damaging). The variant is not previously published nor recorded in population or clinical databases (ExAC, gnomAD, dbSNP, HGMDPro, ClinVar or DECIPHER) (accessed 27th September 2016).

The β-tubulin gene *TUBB* is highly expressed in the developing cortex and monoallelic missense variants have been relatively recently described in a single publication in association with complex cortical dysplasia with other brain malformations-6 (CDCBM6; 615771), Breuss et al. (2012) in 3 postnatally ascertained children via WES. In addition several *de novo* missense variants are reported both in ClinVar and DECIPHER in association with cortical malformations and classified as pathogenic. A low of rate of benign missense variation has been calculated for *TUBB* in the ExAC database (z-score=6.32).

Clinical Review Panel (CRP) interpretation: The p.(Pro287Leu) *TUBB* variant was classified as Likely Pathogenic using ACMG guidelines (Richards, 2015) based on being a *de novo* finding in a gene consistent with presenting phenotype with confirmation of parental identity (PS2); absence from control data (PM1); a missense variant in a gene with a low rate of benign missense variation in a gene in which missense variation is a reported mechanism of disease (PP2); and multiple lines of computational evidence supporting a deleterious impact (PP3). Bi-directional Sanger sequencing technically confirmed a *de novo* finding and the variant was reported likely to be pathogenic, fitting the fetal phenotype and with low recurrence risk. This case in particular illustrates the utility of open-access data sharing to assist interpretation and decision making in the clinical review process of emerging and rare genotype-phenotype associations.

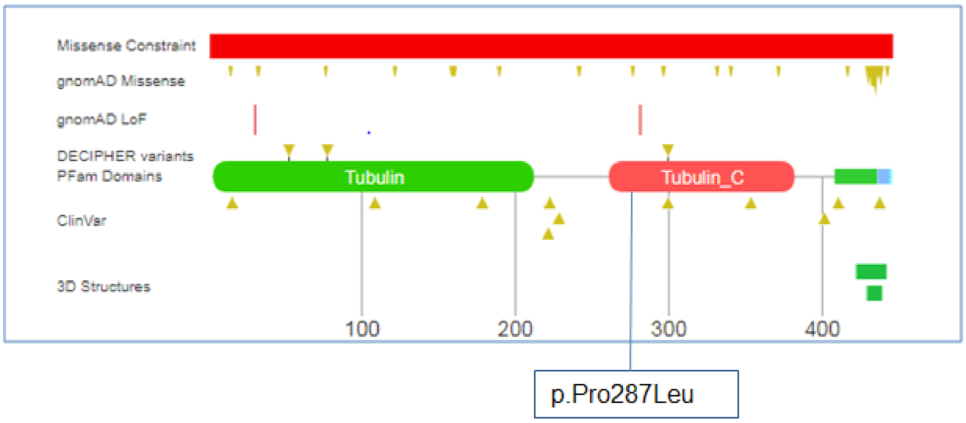

Protein schematic of TUBB gene taken from DECIPHER highlighting variants deposited in ExAC, gnomAD, ClinVar and DECIPHER and location of p.(Pro287Leu) (date 27 Sep 2016)

Case Study 2 (PP0722/ EGAN00001366482)

Clinical Background: Raised nuchal translucency (6.7mm) was detected by USS at 13+2 in a 32 yr old G0P0 woman. QF-PCR and CMA were both normal. Subsequent scans from 17 weeks examining heart, kidneys and spine were all normal. An apparently healthy baby was delivered at 38 weeks weighing 3.1kg.

Whole exome sequencing (WES) variant data: an apparently de novo heterozygous NM_003482.3:c.15535C>T (p.Arg5179Cys) missense variant in the *KMT2D* gene (MIM:602113) was detected in DNA extracted from chorionic villus sampling (CVS). The variant altered a moderately conserved nucleotide (phyloP: 3.92 [-14.1;6.4]) and highly conserved amino acid (to *C. elegans*) towards the –COOH terminal of the protein in exon 48 (NM_003482.3). *In silico* tools predicted a deleterious effect on protein function (SIFT: Deleterious, MutationTaster: disease causing). The variant is not recorded in population databases (ExAC and gnomAD) (accessed 23rd September 2016).

Variants in the *KMT2D* (formerly *MLL2*) gene account for 55 to 80% of patients with Kabuki syndrome, an autosomal dominant, typically *de novo*, condition characterised by a distinct facial phenotype, developmental delay and mild to moderate intellectual disability and heterogeneous internal malformations involving heart, kidneys, gastrointestinal system, skeletal system and eyes (Banka et al. 2012). A diverse mutational spectrum is observed and notably de novo missense variants have been reported to cluster in exon 48 (Banka et al. 2012).
The c.15535C>T variant has been reported previously in association with Kabuki syndrome (Dentici et al. 2015; Bogershausen et al. 2016) in a single postnatal patient (without establishment of inheritance) and a further 2 cases are recorded in ClinVar with conflicting interpretations of pathogenicity. Multiple patients with two different missense changes, most of which are reported to be de novo, have been reported in the same amino acid (c.15536G>A p.(Arg5179His), (Ng et al. 2010; Hannibal et al. 2011; Miyake et al. 2013; Morgan et al. 2015; Bogershausen et al., 2016) and c.15536G>T p.(Arg5179Leu) (Bogershausen et al. 2016).

Clinical Review Panel (CRP) interpretation: The p.Arg5179Cys *KMT2D* variant was initially classified as a variant of uncertain significance (VUS) on account of insufficient or conflicting literature evidence and insufficient clinical evidence of features of Kabuki syndrome on ultrasound investigation. The CRP concluded however that technical validation, clinical reporting with a recommendation to follow-up postnatally were warranted. Following the further reports of the c.15535C>T in ClinVar, the variant was reassessed and considered to be likely pathogenic.

Postnatal follow-up: Clinical genetics review at 18 months following reporting of the PAGE-detected *KMT2D* variant revealed features consistent with a diagnosis of Kabuki syndrome: fetal pads, sacral dimple and arched eyebrows.

Figure 2: Protein schematic of part of KMT2D taken from DECIPHER highlighting variants deposited in ExAC, gnomAD, ClinVar and DECIPHER and location of p.(Arg5179Cys) variant

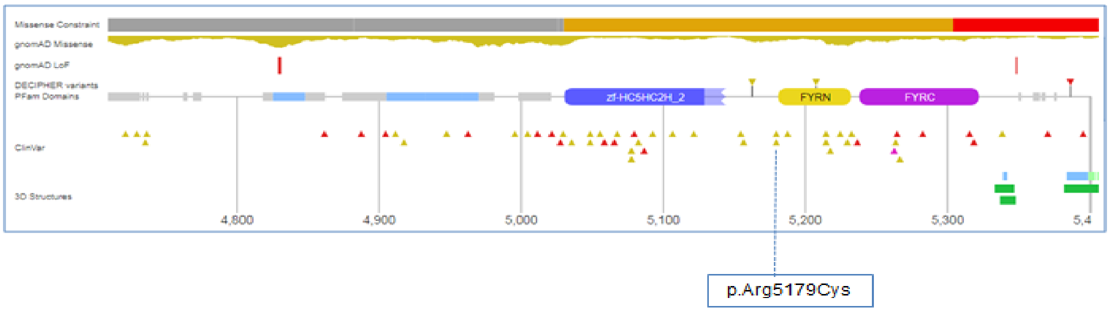

Case Study 3 (PP0258/EGAN00001366802)

Clinical Background: Duodenal atresia (double bubble evident) was detected at 33+3 weeks. Amniocentesis and amniodrainage were carried out at 34+2 weesk. Dilatation of the distal oesophagus to the level of the diaphragm was noted at 35+1 weeks. A female infant weighing 2.8kg was devlivered at 38+1 weeks and admitted to NICU before discharge for on-going care. Both mother and maternal uncle had duodenal atresia.

Whole exome sequencing (WES) variant data: a maternally inherited heterozygous NM_005378.4:c.347T>C (p.Met116Thr) missense variant was detected in DNA extracted from amniocentesis sampling. The variant altered a highly conserved nucleotide (phyloP: 4.32 [-14.1;6.4]) and highly conserved amino acid (to Zebrafish) towards the N-terminal of the protein in exon 2 (NM_005378.4). *In silico* tools predicted a deleterious effect on protein function (SIFT: Deleterious, MutationTaster: disease causing). The variant is not recorded in population databases (ExAC and gnomAD) (February 2017).

Variants in the *MYCN* gene account for over 65% of patients with Feingold Syndrome 1 (Oculodigitoesophagoduodenal Syndrome, ODED Syndrome), an autosomal dominant condition characterised by digital anomalies (shortening of the 2nd and 5th middle phalanx of the hand, clinodactyly of the 5th finger, syndactyly of toes 2-3 and/or 4-5, thumb hypoplasia), microcephaly, facial dysmorphism (short palpebral fissures and micrognathia), gastrointestinal atresias (primarily esophageal and/or duodenal), and mild to moderate learning disability. Approximately 60% of cases have an affected parent (Marcelis, 2008). All pathogenic variants currently identified are present in either exon 2 or 3 of *MYCN* and include protein truncating variants, deletions and missense variants in the DNA binding domain (Marcelis, 2008), as opposed to the Myc amino-terminal region observed in the current case.

Clinical Review Panel (CRP) interpretation: The p.Met116Thr variant was considered to be likely relevant to the fetal anomaly considering the *in silico* predictions of pathogenicity, allelic segregation and the clear relationship between the fetal and family history of duodenal atresia and pathogenic variants in *MYCN*. Confirmation of segregation in the affected maternal uncle was recommended. This case illustrated the importance of the availability of family history for variant interpretation and correct bioinformatics filtering.

Figure 3: Protein schematic of MYCN taken from DECIPHER highlighting variants deposited in ExAC, gnomAD, ClinVar and DECIPHER and location of p.(Met116Thr) variant

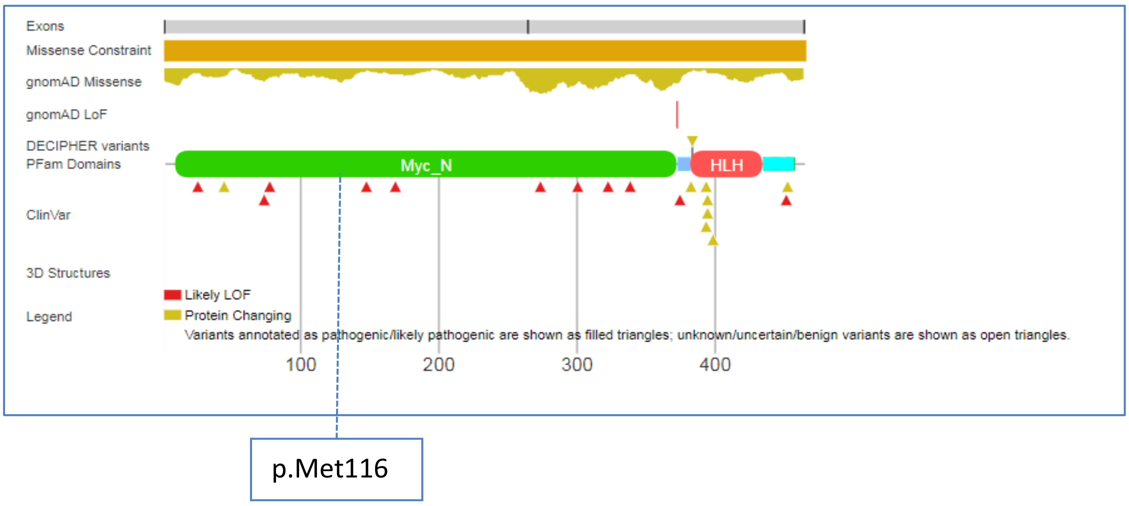

References

1000 Genomes Project Consortium, Auton A, Brooks LD, Durbin RM, Garrison EP, Kang HM, Korbel JO, Marchini JL, McCarthy S, McVean GA, Abecasis GR. A global reference for human genetic variation. Nature. 2015;526:68-74

Alamillo CL, Powis Z, Farwell K, Shahmirzadi L, Weltmer EC, Turocy J, Lowe T, Kobelka C, Chen E, Basel D, Ashkinadze E, D'Augelli L, Chao E, Tang S. Exome sequencing positively identified relevant alterations in more than half of cases with an indication of prenatal ultrasound anomalies. Prenat Diagn. 2015 Nov;35:1073-8

Banka S, Veeramachaneni R, Reardon W, Howard E, Bunstone S, Ragge N, Parker MJ, Crow YJ, Kerr B, Kingston H, Metcalfe K, Chandler K, Magee A, Stewart F, McConnell VP, Donnelly DE, Berland S, Houge G, Morton JE, Oley C, Revencu N, Park SM, Davies SJ, Fry AE, Lynch SA, Gill H, Schweiger S, Lam WW, Tolmie J, Mohammed SN, Hobson E, Smith A, Blyth M, Bennett C, Vasudevan PC, García-Miñaúr S, Henderson A, Goodship J, Wright MJ, Fisher R, Gibbons R, Price SM, C de Silva D, Temple IK, Collins AL, Lachlan K, Elmslie F, McEntagart M, Castle B, Clayton-Smith J, Black GC, Donnai D. How genetically heterogeneous is Kabuki syndrome?: MLL2 testing in 116 patients, review and analyses of mutation and phenotypic spectrum. Eur J Hum Genet. 2012;20:381-8

Bögershausen N, Gatinois V, Riehmer V, Kayserili H, Becker J, Thoenes M, Simsek-Kiper PÖ, Barat-Houari M, Elcioglu NH, Wieczorek D, Tinschert S, Sarrabay G, Strom TM, Fabre A, Baynam G, Sanchez E, Nürnberg G, Altunoglu U, Capri Y, Isidor B, Lacombe D, Corsini C, Cormier-Daire V, Sanlaville D, Giuliano F, Le Quan Sang KH, Kayirangwa H, Nürnberg P, Meitinger T, Boduroglu K, Zoll B, Lyonnet S, Tzschach A, Verloes A, Di Donato N, Touitou I, Netzer C, Li Y, Geneviève D, Yigit G, Wollnik B. Mutation Update for Kabuki Syndrome Genes KMT2D and KDM6A and Further Delineation of X-Linked Kabuki Syndrome Subtype 2. Hum Mutat. 2016;37:847-64

Breuss M, Heng JI, Poirier K, Tian G, Jaglin XH, Qu Z, Braun A, Gstrein T, Ngo L, Haas M, Bahi-Buisson N, Moutard ML, Passemard S, Verloes A, Gressens P, Xie Y, Robson KJ, Rani DS, Thangaraj K, Clausen T, Chelly J, Cowan NJ, Keays DA. Mutations in the β-tubulin gene TUBB5 cause microcephaly with structural brain abnormalities. Cell Rep. 2012;2:1554-62

Carss KJ, Hillman SC, Parthiban V, McMullan DJ, Maher ER, Kilby MD, Hurles ME. Exome sequencing improves genetic diagnosis of structural fetal abnormalities revealed by ultrasound. Hum Mol Genet. 2014 Jun 15;23:3269-77

Conomos MP, Reiner AP, Weir BS, Thornton TA. Model-free Estimation of Recent Genetic Relatedness. Am J Hum Genet. 2016;98:127-48

Dentici ML, Di Pede A, Lepri FR, Gnazzo M, Lombardi MH, Auriti C, Petrocchi S, Pisaneschi E, Bellacchio E, Capolino R, Braguglia A, Angioni A, Dotta A, Digilio MC, Dallapiccola B. Kabuki syndrome: clinical and molecular diagnosis in the first year of life. Arch Dis Child. 2015;100:158-64

Drury S, Williams H, Trump N, Boustred C; GOSGene, Lench N, Scott RH, Chitty LS. Exome sequencing for prenatal diagnosis of fetuses with sonographic abnormalities. Prenat Diagn. 2015;35:1010-7

Hannibal MC, Buckingham KJ, Ng SB, Ming JE, Beck AE, McMillin MJ, Gildersleeve HI, Bigham AW, Tabor HK, Mefford HC, Cook J, Yoshiura K, Matsumoto T, Matsumoto N, Miyake N, Tonoki H, Naritomi K, Kaname T, Nagai T, Ohashi H, Kurosawa K, Hou JW, Ohta T, Liang D, Sudo A, Morris CA, Banka S, Black GC, Clayton-Smith J, Nickerson DA, Zackai EH, Shaikh TH, Donnai D, Niikawa N, Shendure J, Bamshad MJ. Spectrum of MLL2 (ALR) mutations in 110 cases of Kabuki syndrome. Am J Med Genet A. 2011;155A:1511-6

King DA, Fitzgerald TW, Miller R, Canham N, Clayton-Smith J, Johnson D, Mansour S, Stewart F, Vasudevan P, Hurles ME; DDD Study. A novel method for detecting uniparental disomy from trio genotypes identifies a significant excess in children with developmental disorders. Genome Res. 2014;24:673-87

Lek M, Karczewski KJ, Minikel EV, Samocha KE, Banks E, Fennell T, O'Donnell-Luria AH, Ware JS, Hill AJ, Cummings BB, Tukiainen T, Birnbaum DP, Kosmicki JA, Duncan LE, Estrada K, Zhao F, Zou J, Pierce-Hoffman E, Berghout J, Cooper DN, Deflaux N, DePristo M, Do R, Flannick J, Fromer M, Gauthier L, Goldstein J, Gupta N, Howrigan D, Kiezun A, Kurki MI, Moonshine AL, Natarajan P, Orozco L, Peloso GM, Poplin R, Rivas MA, Ruano-Rubio V, Rose SA, Ruderfer DM, Shakir K, Stenson PD, Stevens C, Thomas BP, Tiao G, Tusie-Luna MT, Weisburd B, Won HH, Yu D, Altshuler DM, Ardissino D, Boehnke M, Danesh J, Donnelly S, Elosua R, Florez JC, Gabriel SB, Getz G, Glatt SJ, Hultman CM, Kathiresan S, Laakso M, McCarroll S, McCarthy MI, McGovern D, McPherson R, Neale BM, Palotie A, Purcell SM, Saleheen D, Scharf JM, Sklar P, Sullivan PF, Tuomilehto J, Tsuang MT, Watkins HC, Wilson JG, Daly MJ, MacArthur DG; Exome Aggregation Consortium. Analysis of protein-coding genetic variation in 60,706 humans. Nature. 2016;536:285-91

Manichaikul A, Mychaleckyj JC, Rich SS, Daly K, Sale M, Chen WM. Robust relationship inference in genome-wide association studies. Bioinformatics. 2010;26:2867-73

Marcelis CL, Hol FA, Graham GE, Rieu PN, Kellermayer R, Meijer RP, Lugtenberg D, Scheffer H, van Bokhoven H, Brunner HG, de Brouwer AP. Genotype-phenotype correlations in MYCN-related Feingold syndrome. Hum Mutat. 2008;29(9):1125-32.

McLaren W, Gil L, Hunt SE, Riat HS, Ritchie GR, Thormann A, Flicek P, Cunningham F. The Ensembl Variant Effect Predictor. *Genome Biology* 2016;17:122

Miyake N, Koshimizu E, Okamoto N, Mizuno S, Ogata T, Nagai T, Kosho T, Ohashi H, Kato M, Sasaki G, Mabe H, Watanabe Y, Yoshino M, Matsuishi T, Takanashi J, Shotelersuk V, Tekin M, Ochi N, Kubota M, Ito N, Ihara K, Hara T, Tonoki H, Ohta T, Saito K, Matsuo M, Urano M, Enokizono T, Sato A, Tanaka H, Ogawa A, Fujita T, Hiraki Y, Kitanaka S, Matsubara Y, Makita T, Taguri M, Nakashima M, Tsurusaki Y, Saitsu H, Yoshiura K, Matsumoto N, Niikawa N. MLL2 and KDM6A mutations in patients with Kabuki syndrome. Am J Med Genet A. 2013;161A:2234-43

Morgan AT, Mei C, Da Costa A, Fifer J, Lederer D, Benoit V, McMillin MJ, Buckingham KJ, Bamshad MJ, Pope K, White SM. Speech and language in a genotyped cohort of individuals with Kabuki syndrome. Am J Med Genet A. 2015;167:1483-92

Narasimhan V, Danecek P, Scally A, Xue Y, Tyler-Smith C, Durbin R. BCFtools/RoH: a hidden Markov model approach for detecting autozygosity from next-generation sequencing data. Bioinformatics. 2016;32:1749-51

Ng SB, Bigham AW, Buckingham KJ, Hannibal MC, McMillin MJ, Gildersleeve HI, Beck AE, Tabor HK, Cooper GM, Mefford HC, Lee C, Turner EH, Smith JD, Rieder MJ, Yoshiura K, Matsumoto N, Ohta T, Niikawa N, Nickerson DA, Bamshad MJ, Shendure J. Exome sequencing identifies MLL2 mutations as a cause of Kabuki syndrome. Nat Genet. 2010;42:790-3

Pangalos C, Hagnefelt B, Lilakos K, Konialis C. First applications of a targeted exome sequencing approach in fetuses with ultrasound abnormalities reveals an important fraction of cases with associated gene defects. Peer J 2016;4:e1955

Ramu A, Noordam MJ, Schwartz RS, Wuster A, Hurles ME, Cartwright RA, Conrad DF. DeNovoGear: de novo indel and point mutation discovery and phasing. Nat Methods. 2013;10:985-7

UK10K Consortium, Walter K, Min JL, Huang J, Crooks L, Memari Y, McCarthy S, Perry JR, Xu C, Futema M, Lawson D, Iotchkova V, Schiffels S, Hendricks AE, Danecek P, Li R, Floyd J, Wain

LV, Barroso I, Humphries SE, Hurles ME, Zeggini E, Barrett JC, Plagnol V, Richards JB, Greenwood CM, Timpson NJ, Durbin R, Soranzo N. The UK10K project identifies rare variants in health and disease. Nature. 2015;526:82-90

Van der Auwera GA, Carneiro MO, Hartl C, Poplin R, Del Angel G, Levy-Moonshine A, Jordan T, Shakir K, Roazen D, Thibault J, Banks E, Garimella KV, Altshuler D, Gabriel S, DePristo MA. From FastQ data to high confidence variant calls: the Genome Analysis Toolkit best practices pipeline. Curr Protoc Bioinformatics 2013;43:11.10.1-33

Vora NL, Powell B, Brandt A, Strande N, Hardisty E, Gilmore K, Foreman AKM, Wilhelmsen K, Bizon C, Reilly J, Owen P, Powell CM, Skinner D, Rini C, Lyerly AD, Boggess KA, Weck K, Berg JS, Evans JP. Prenatal exome sequencing in anomalous fetuses: new opportunities and challenges. Genet Med. 2017 May 18. Doi: 10.1038/gim.2017.33. [Epub ahead of print] PubMed PMID: 28518170.

Westerfield LE, Stover SR, Mathur VS, Nassef SA, Carter TG, Yang Y, Eng CM, Van den Veyver IB. Reproductive genetic counseling challenges associated with diagnostic exome sequencing in a large academic private reproductive genetic counseling practice. Prenat Diagn. 2015;35:1022-9

Wright CF, Fitzgerald TW, Jones WD, Clayton S, McRae JF, van Kogelenberg M, King DA, Ambridge K, Barrett DM, Bayzetinova T, Bevan AP, Bragin E, Chatzimichali EA, Gribble S, Jones P, Krishnappa N, Mason LE, Miller R, Morley KI, Parthiban V, Prigmore E, Rajan D, Sifrim A, Swaminathan GJ, Tivey AR, Middleton A, Parker M, Carter NP, Barrett JC, Hurles ME, FitzPatrick DR, Firth HV; DDD study. Genetic diagnosis of developmental disorders in the DDD study: a scalable analysis of genome-wide research data. Lancet. 2015;385:1305-14

Yang Y, Muzny DM, Reid JG, Bainbridge MN, Willis A, Ward PA, Braxton A, Beuten J, Xia F, Niu Z, Hardison M, Person R, Bekheirnia MR, Leduc MS, Kirby A, Pham P, Scull J, Wang M, Ding Y, Plon SE, Lupski JR, Beaudet AL, Gibbs RA, Eng CM. Clinical whole-exome sequencing for the diagnosis of mendelian disorders. N Engl J Med. 2013;369:1502-11

Yang Y, Muzny DM, Xia F, et al. Molecular findings among patients referred for clinical whole-exome sequencing. JAMA 2014;312:1870–1879.

Yates CL, Monaghan KG, Copenheaver D, Retterer K, Scuffins J, Kucera CR, Friedman B, Richard G, Juusola J. Whole-exome sequencing on deceased fetuses with ultrasound anomalies: expanding our knowledge of genetic disease during fetal development. Genet Med. 2017 Apr 20. doi: 10.1038/gim.2017.31. [Epub ahead of print] PubMed PMID: 28425981.
